# (Not) one of us: The overrepresentation of elites in politics erodes political trust

**DOI:** 10.1111/bjso.12885

**Published:** 2025-04-05

**Authors:** Rebekka Kesberg, Matthew J. Easterbrook

**Affiliations:** ^1^ University of Sussex Brighton UK; ^2^ University of Amsterdam Amsterdam

**Keywords:** meritocracy, political trust, private education, representation, social class, stereotypes

## Abstract

Citizens in democracies are increasingly dissatisfied with democratic governance, distrustful of elected officials and view politicians as aloof and detached. We argue that this is, in part, due to the overrepresentation of elites in political office. We conducted four studies (*N* = 2009) in the U.K. focusing on the education sector. That is, we explore the impact of the overrepresentation of privately educated individuals—who represent >7% of the population but 30%–70% of the political cabinet—on political trust. Studies 1a and 1b measured perceptions and stereotypes of politicians, and Studies 2–4 manipulated the proportion of privately educated politicians in political institutions. Results show that trust in political institutions is eroded when there is overrepresentation of those educated in the private sector. We explore boundary conditions showing that effects are stronger for those who question meritocratic principles in the educational sector and that the effect is mediated through perceptions of deservingness.

## INTRODUCTION

Democracies across the globe are increasingly characterized by high levels of political dissatisfaction and distrust among their citizens (Noury & Roland, 2020). Many citizens have come to view politicians as detached from the worries of ordinary people and parliaments as elitist institutions. This is understandable, perhaps, if we consider that, in many democracies, politicians are often from starkly different socioeconomic backgrounds than most of the people they represent. Indeed, political offices tend to be dominated by the higher educated, the rich and/or people from the upper class. For example, the majority of the U.S. Congress are millionaires, yet millionaires represent just 9% of the U.S. population. Similarly, in the Netherlands, 40% of politicians have an upper‐class background, compared with 20% in the population, and just 10% are from working‐class backgrounds in contrast to 35% of the population (Bovens & Wille, 2017). These mismatches conflict with the central ideas of representative democracy, which state that elected representatives should exemplify the people they represent, and that the composition of parliaments should broadly reflect the demographic characteristics of the population (Pettit, 2009; Rawls, 2016; Skinner, 2005).

In the U.K., the context of the investigation we report here, one attribute that is indicative of being higher class is vastly overrepresented in politics: having been educated in the private, fee‐paying sector.^1^ Under seven percent of the population are privately educated, reflecting the vast costs involved: an average of £39,006 a year for full boarding, which is well over the median post‐tax salary in the U.K. (£27,583). Private education is therefore reserved for the wealthy elite, far beyond the means of most (Sibietaa, 2023). Yet, those who have been privately educated are more than five times as likely to occupy prestigious social positions—including high‐level business leaders, public servants and media professionals—as those who were state educated (Sutton Trust & Social Mobility Commission, 2019), with many elite sectors almost exclusively recruiting individuals who have been privately educated (Milburn, 2009, 2012). In British politics, the percentage of members of parliament who were privately educated is around 35%. In the central cabinet, the figure tends to vary between 30% and 70%. Private education, then, is the reserve of the elite and vastly overrepresented in the upper echelons of British society.

What is the impact of this overrepresentation? We focus here on the political domain and address the following questions: To what extent do citizens feel represented by a parliament that is dominated by the wealthy elite? How does the overrepresentation of privately educated individuals in political institutions impact citizens' trust in those institutions? How are privately educated politicians perceived in terms of their deservedness and merit?

In this paper, we report four studies that explore the extent to which individuals feel that they can trust and are represented by politicians from elite backgrounds. We test whether and how the proportional overrepresentation of privileged individuals in political institutions impacts citizens' feelings of being represented by those institutions and, more importantly, the extent to which they feel they can trust them. We contribute to the existing literature in two main ways. First, we expand the psychological literature on elite representation in politics to empirically investigate how educational sector—that is, whether one attended a state‐ or privately‐funded school—is related to political trust. This is an important addition to the current literature that demonstrates *education level*, primarily whether one is university‐educated or not, impacts identity processes and political trust (Bovens & Wille, 2017), but which has not investigated the role of education sector. We argue that education sector—unlike level of education—is a robust and valid measure of social class in the U.K., one that captures access to elite institutions (Milburn, 2009; 2012) as well as important aspects of class socialization (Easterbrook et al., 2023).

Second, we explore a potential boundary condition for the relation between education sector and political trust: the belief that schooling is meritocratic. This belief refers to the idea that individuals' academic opportunities and success is solely determined by their abilities and efforts, instead of factors like social class, wealth or family background (Darnon et al., 2018). This belief can be used to justify educational inequalities claiming that equal opportunities exit and that everyone can succeed in school. Yet, the U.K. education sector is separated into a free state education and a costly private education, which challenges the assumption of merit and instead highlights the roles of family background and wealth. Currently, the extent to which merit beliefs are used to justify inequalities based on education sector is unclear. For those who consider schooling meritocratic, the advantages associated with private education, as well as having the necessary resources to pay for it, may be considered legitimate reflections of merit. For those who do not consider schooling as meritocratic, by contrast, the benefits associated with being privately educated are likely to be considered unjust because they violate the principles of meritocracy, and privately educated individuals are likely to be evaluated negatively. We investigate the role of belief in school meritocracy in social perceptions and argue that political trust varies as a function of politician's social class and individual's belief in school meritocracy.

### Background

Research has found that people tend to show a social preference for individuals and groups with higher socioeconomic status (SES; Grigoryan et al., 2023), while stigmatizing those with low SES (Kuppens et al., 2018). This has been explained in terms of social stereotypes, with people inferring a group's competence from their social status and by the widespread endorsement of a meritocratic ideology, which legitimizes and justifies status positions (ibid.). These social perceptions translate into political attitudes, with those displaying a stronger preference for the higher educated also showing more political trust, being more satisfied with democracy and being less likely to be populist (van Noord et al., 2023). Thus, *education level* is related to perceptions of political trust, primarily through social stereotypes and meritocratic ideology.

Yet, and by contrast, perceiving markers of high status among politicians increases less‐educated citizens support for aggression against the Government (Noordzij et al., 2023), and lower‐educated citizens tend to perceive politicians negatively, controlling, and as culturally distant *others* (Fiske & Durante, 2014; Noordzij et al., 2021). Furthermore, perceptions that there is a closed‐ranked elite who are against ‘the people’ are key tenets of political populism (Spruyt et al., 2025), implying that the highly educated elite are perceived negatively and associated with lower political trust among citizens.

We suggest that these contrasting findings can be explained by the different conceptualizations of social class that the two perspectives offer. The former—which finds higher‐educated individuals are perceived positively—argues that status‐based attributes, such as education or income, are individualized (Easterbrook et al., 2020, 2023) and equated with competence (Cuddy et al., 2008) so that status is distributed according to the principles of a meritocracy (Kuppens et al., 2018). This justifies and legitimizes status positions (Batruch et al., 2023). The latter perspective—which finds elites are perceived negatively—takes a more group‐based approach to class, where the elite are perceived as a restricted, entitative and biased group that wields illegitimate power to further their ingroup interests (Spruyt et al., 2025).

Privately educated politicians are what many in the UK consider to define and represent the establishment or the elite (Jones, 2015), a group that is often positioned as directly opposed to the general public (Spruyt et al., 2025). Furthermore, the education sector relates to a time of children's socialization, where cultural orientations that define social‐class groups are embedded and imbibed (Bourdieu & Passeron, 1977). Therefore, building on the group‐based approach, we argue that the education sector—much more so than the education level—is likely to be perceived as a group‐based rather than individualized status characteristic.

We contribute to the literature by exploring differences in group perception based on education sector and the diverging consequences of these perceptions compared to those associated with education level. We postulate that the privately educated are perceived as a highly visible, distinct, impermeable and elite outgroup for most citizens. These attributes lead to the group being perceived as highly entitative, that is, ‘bonded together in a coherent unit’ (Lickel et al., 2000, p. 224), in line with a group‐based approach of social class. By contrast, we assume that the higher educated are perceived as a less cohesive group with permeable group boundaries which allows for status characteristics to be individualized (Van Doesum et al., 2017). We therefore expect that privately educated politicians, while potentially perceived as competent due to their elite status, will be perceived as an outgroup and therefore negatively. Thus, we expect that the vast overrepresentation of the privately educated in politics is likely to erode political trust and feelings of representation.

### The role of meritocracy

The extent to which privately educated politicians are negatively perceived is likely to depend on how meritocratic people believe the education system is. Political, economic and education systems tend to be perceived as legitimate and fair if they are perceived as meritocratic, such that rewards are distributed based on ability, effort and talent, rather than wealth or social class (Echebarria Echabe, 2014; Ledgerwood et al., 2011; Zhai et al., 2023). Those who believe that the education system, with its separation of state and private sectors, is meritocratic and therefore just—despite its inequalities (Darnon et al., 2018)—are also likely to perceive that those who go to elite, private schools deserve their privilege and the success it brings them.

By contrast, those who eschew the notion that the school system is meritocratic are likely to perceive privately educated politicians as unworthy of their privileged positions, and as symbols of a broken and unjust system. Indeed, the belief in school meritocracy has been shown to legitimize inequality (Batruch et al., 2019), implying that perceiving school as a meritocracy will reduce negative perceptions towards the privately educated. Thus far, however, the belief in school meritocracy has been primarily implicated in legitimizing the low status of low SES groups (Wiederkehr et al., 2015). We advance this literature by investigating the role of meritocratic beliefs in the legitimization of the high status of elites in politics. We expect, therefore, that whether an overrepresentation of privately educated individuals in political offices reduces perceptions that politicians and political institutions are legitimate and trustworthy will depend on an individual's belief in school meritocracy.

## THE CURRENT RESEARCH

The current research explores the impact of the education sector as a proxy for social class on citizens' trust of politicians and governments. In addition to political trust, we also explore the perception of (political) representation, which refers to the perception that citizens' opinions, voices and perspectives are salient in the political decision‐making process. Previous work focuses on how representatives aim to represent their constituents or on what makes them representative (Disch, 2011; Dovi, 2018; Mansbridge & Macedo, 2019), but little work has investigated *how* the represented perceive representatives (Lavi et al., 2021). This is important, however, because recent work shows that the extent to which citizens feel represented strongly impacts voter turnout, trust in institutions and satisfaction with democracy (Blais et al., 2014; Dunn, 2015; Wessels, 2011).

We draw on the social perception and stereotype content literatures to assess whether politicians are perceived differently depending on whether they were educated in the private or state sector. According to the stereotype content model (Cuddy et al., 2008), high‐status groups tend to be perceived as agentic and competent, and competitor groups as cold and lacking communion. This implies that privately educated politicians, if perceived as elites in conflict with the general public (Spruyt et al., 2025), may be perceived as more competent yet colder than state‐educated politicians. Indeed, social‐class stereotypes tend to be ambivalent in nature, with high‐class groups tending to be perceived as agentic, competent and cold, while low‐status groups are perceived as incompetent, not agentic and warm. These ambivalent stereotypes act to justify the status quo of status relations (Durante & Fiske, 2017). However, the negative perceptions of high‐status politicians found in previous research (Noordzij et al., 2023) suggest that it is possible that privately educated politicians will be perceived negatively on all social dimensions. We explore these and other key dimensions of social perceptions across multiple studies.

The research is driven by three key research questions (RQs)

RQ 1: Do perceptions of politicians and social groups vary depending on whether they were privately or state educated?
RQ 2: How does the composition of political institutions in terms of the proportion of privately and state‐educated individuals affect trust in those institutions and feelings of representation?
RQ 3: Does belief in school meritocracy act as a hierarchy legitimizing ideology that justifies the position of privately educated politicians?

We conducted four studies to explore these research questions. Study 1a investigates whether perceptions of politicians vary depending on whether those politicians were state or privately educated. Study 1b explores individuals' perceptions of the privately educated and the state educated as social groups and compares them to various other high‐ and low‐status groups. Studies 2, 3 and 4 experimentally tested the impact of overrepresentation in fictional governments. Across these studies, we manipulate the extent to which privately educated politicians were overrepresented in political office. Study 2 manipulated who was overrepresented (i.e. state educated or privately educated), while Study 3 included information about the proportion of the different groups within a fictional society to serve as an anchor for the judgment of overrepresentation. Across Studies 1–3, we measure and analyze meritocratic beliefs as a moderator. Finally, in Study 4, we manipulated merit perception to explore its impact directly. We show that privately educated politicians, and political institutions in which they are overrepresented, are perceived as less trustworthy and representative, but not less competent. Individuals who endorse meritocratic beliefs perceive fewer differences between privately educated politicians and state‐educated politicians.

### Transparency and openness

All studies were preregistered and designed to have 80% power to detect small to medium effects. Preregistration, data and syntax are available on OSF (https://osf.io/etsxj/?view_only=e465cbbdae584eab9cfea762f4749ca8). Detailed descriptions of each sample are displayed in Table 1, including information on data exclusions, and additional analyses are in Appendix S1. In the main manuscript, we report all central measures, and in Appendix S1, we report all additional measures. Data were analysed using R, version 4.0.0 (R Core Team, 2024). Further, in the SOM, we report Study 1b in detail and report additional analyses. First, we report all analyses that only included state‐educated participants. The key results remain the same (Tables S25–S41). We also report results using the education sector of participants as a moderator (Tables S42–S48).

**TABLE 1:** Sample description.

	Study 1a	Study 1b	Study 2	Study 3	Study 4
Sample size	209	201	639	391	622
Excluded did not consent	0	1	17	2	0
Excluded failed attention check	4	4	10	5	8
Final sample size	205	196	612	384	614
Age	46.24 (14.70)	44.27 (14.24)	43.38 (14.17)	41.67 (13.91)	41.80 (13.85)
Gender	Female = 125 Male = 80	Female = 103 Male = 89 Other = 4	Female = 290 Male = 312 Other = 10	Female = 198 Male = 180 Other = 6	Female = 290 Male = 321 Other = 3
Children	Yes = 118 No = 90	Yes = 111 No = 89	Yes = 279 No = 343	Yes = 173 No = 215	Yes = 284 No = 334
Occupation	Paid Work = 150 In Education = 5 Unemployment = 10 Permanently sick or disabled = 7 Retired = 23 Housework = 14	Paid Work = 142 In Education = 7 Unemployment = 11 Permanently sick or disabled = 3 Retired = 21 Housework = 16	Paid Work = 423 In Education = 28 Unemployment = 53 Permanently sick or disabled = 16 Retired = 63 Housework = 16	Paid Work = 279 In Education = 21 Unemployment = 25 Permanently sick or disabled = 15 Retired = 26 Housework = 22	Paid Work = 438 In Education = 32 Unemployment = 51 Permanently sick or disabled = 13 Retired = 50 Housework = 38
Highest level of education	<Bachelor = 91 ≥Bachelor = 118	<Bachelor = 86 ≥Bachelor = 114	<Bachelor = 269 ≥Bachelor = 353	<Bachelor = 218 ≥Bachelor = 170	<Bachelor = 263 ≥Bachelor = 386
% Of sample attending private school	14%	14%	15%	16%	17%
% Of sample's children attending private school	7%	9%	7%	11%	12%
Income (1 = not comfortable; 5 = very comfortable)	2.96 (1.03)	3.00 (0.96)	3.10 (1.02)	2.93 (0.72)	2.91 (1.02)
Urbanization	Open countryside = 7 Village/small town = 67 Medium to large town = 66 Suburbs = 41 Big city = 28	Open countryside = 8 Village/small town = 65 Medium to large town = 63 Suburbs = 40 Big city = 24	Open countryside = 27 Village/small town = 180 Medium to large town = 176 Suburbs = 146 Big city = 93	Open countryside = 11 Village/small town = 124 Medium to large town = 114 Suburbs = 86 Big city = 53	Open countryside = 15 Village/small town = 198 Medium to large town = 199 Suburbs = 135 Big city = 75
% Of white British ethnicity in the sample	89%	89%	88%	88%	90%
Political orientation (0–10)	4.10 (2.44)	4.09 (2.64)	4.03 (2.48)	3.95 (2.50)	4.00 (2.26)
Belief in school meritocracy	2.73 (0.91)	2.94 (0.94)	2.98 (0.93)	2.97 (0.92)	2.95 (0.95)

## STUDY 1

Study 1a focuses on exploring variations in the perception of politicians with respect to key dimensions of social perception (agency and communion), trust and representation depending on the education sector (private school versus state school). Study 1b, reported in Appendix S1, additionally explores how different social groups—including the privately educated, the state educated and politicians—are perceived by U.K. society.

### Method

#### Participants and experimental design

The final sample consisted of *N* = 205 U.K.‐based participants (125 women, 80 men; *M*_Age_ = 46.24, SD_Age_ = 14.70) who were recruited via Prolific Academics. Participants read six vignettes of fictional politicians in randomized order. Each participant was randomly shown one of two versions of each vignette, one in which the politician attended a state school or another in which the politician attended a private school (counter‐balanced design). After each vignette, participants indicated their perception of the politician regarding agency, communion, toxicity and trust. After rating all politicians, participants filled out multiple questionnaires about social and political attitudes, provided socio‐demographic information, were fully debriefed and compensated.

#### Measures

##### Communion, agency and toxicity

We measured perception of the politicians with 17 items on a scale from 1 (Not at all) to 7 (Extremely): *[Name of fictional politician] is (1) decisive, (2) confident, (3) capable, (4) intelligent, (5) cooperative, (6) out of touch with the ordinary people, (7) moral, (8) honest, (9) domineering, (10) corrupt*, *(11) eloquent, (12) has integrity, (13) reliable, (14) opportunistic, (15) manipulative, (16) friendly and (17) well‐connected*. Factor analysis revealed three factors. First, cooperative, moral, honest, reliable, has integrity and friendly formed the communion factor. Second, decisive, confident, capable and intelligent formed the agency factor. Third, the items out of touch, domineering, corrupt, opportunistic and manipulative loaded on another factor, which we labelled toxicity due to its resemblance to toxic leadership. Toxic leadership and influence refer to the detrimental behaviours and practices of individuals or groups in positions of authority (Ahmed et al., 2024; Gandolfi & Stone, 2022). These behaviours include being disconnected from the needs and realities of others (out of touch), exerting excessive control (domineering), engaging in unethical or illegal activities (corrupt), taking advantage of situations for personal gain (opportunistic) and manipulating others for personal benefit (manipulative). The items were inspired by Abele et al. (2016), Cuddy et al. (2008), as well as research on political leadership skills (for an overview, see Aaldering & Vliegenthart, 2016).

##### Trust, representation and respect

Participants indicated the extent to which they trust, respect and feel represented by a council—which the politician is a member of—on a scale from 1 (Not at all) to 11 (Completely). The item for trust reads ‘I would trust that council’; the item for respect reads ‘I would respect that council’; and the item for representation reads ‘I would feel represented by that council’. Cronbach's alphas > .78.

##### Belief in school meritocracy

Belief in school meritocracy was measured with four items on a scale from 1 (Not at all) to 5 (Very much) (Wiederkehr et al., 2015). An example item reads ‘Everyone has the same chances to succeed at school’. Internal consistency was high (Cronbach's alpha = .86).

##### Political orientation

Individuals indicated their political orientation using two items (i.e. concerning economic issues and concerning moral issues) on a scale from 0 (Left) to 10 (Right). The two items were highly correlated (*r* = .79) and combined into one scale.

##### Institutional trust

Participants indicated trust in three U.K. institutions (i.e. parliament, politicians and the legal system) on a scale from 0 (No trust at all) to 10 (Complete trust). Internal consistency was high (Cronbach's alpha = .86), and items were combined into one trust scale.

##### Demographics

Participants indicated their age, gender, level of education, income, urbanization, main occupation, ethnicity, whether they have children and whether they or their children attended private school.

### Results

**FIGURE 1:**
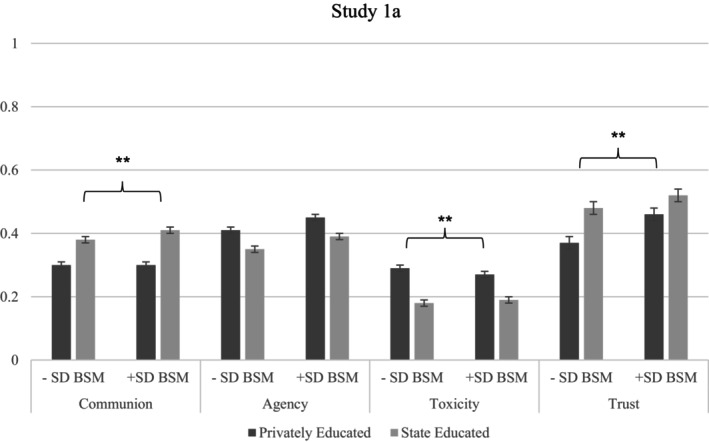
DV's by BSM and politician's Education in Study 1a. **p < .01

Running multilevel regressions to account for the nested structure of the data (i.e. treatment nested within participants), we found privately educated politicians were perceived as less communal, less trustworthy, more toxic and more agentic compared with state‐educated politicians. We again investigated whether BSM moderated these perceptions. Individuals with high (compared with low) BSM perceived privately educated and state‐educated politicians as more similar on communion, toxicity and trust, but there was no significant interaction for agency (see Figure 1, Tables S1–S3).

### Discussion Study 1a and 1b

Study 1a shows that perception of politicians depends on the education sector. Individual politicians who attended private school are seen as less communal, more toxic and less trustworthy. Study 1b demonstrates that these perceptions transcend to the whole social group, that is, to people who are privately educated in general as well as to politicians in general. We also found that privately educated individuals were perceived as a highly entitative group. See Appendix S1 for all results of Study 1b (see Figure S1, Tables S4–S10). Prior research shows that groups that are perceived as highly entitative are also perceived to be more agentic, threatening and malevolent (Abelson et al., 1982; Dasgupta et al., 1999). Furthermore, in highly entitative groups, impressions of one group member are more likely to be generalized to other group members (Crawford et al., 2002). This implies that the perception of whole political institutions could be tarnished by the negative perceptions of one (or a few) privately educated politicians.

## STUDY 2

This study has two parts. Part I builds on the above findings by exploring the extent to which perceptions of political institutions are shaped by the proportion of members who were privately educated. We test whether the ratio of privately and state‐educated individuals within an institution impacts trust and feelings of representation. In part II, we investigate whether applicants for an internship with the institution are rated differently depending on whether they were privately or state‐educated. Part II examines peripheral effects of the composition of institutions. We explore whether participants assume that applicants will be less welcomed in elite‐dominated institutions. If that is indeed the case, it would speak against the notion of merit and instead highlight the role of social connections and privilege in moving ahead in political offices. It would further indicate that people do not believe in fair or equal treatment, again speaking against the notion of meritocracy.

### Method

#### Participants and experimental design

The final sample consisted of 612 U.K. participants (290 women, 312 men, 10 other/not specified; *M*_Age_ = 43.38, SD_Age_ = 14.17) who were recruited via Prolific Academics. In part I, participants were randomly allocated to one of three conditions. In each condition, participants saw a fictional town council consisting of five members. Participants read information about each council member, including the education sector. In the *state‐majority condition* (*n* = 206), four council members were state‐educated, and one was privately educated. In the *private‐majority condition* (*n* = 202), four members were privately educated, and one member was state‐educated. In the *control condition* (*n* = 204), no information about the education sector was given. Participants stayed on the council page for at least 90 s and indicated afterwards which information was presented. In the two experimental conditions, more than 95% indicated that education information was provided; in the control condition, ~90% indicated that no education information was provided, and around 7% were not sure. Participants then indicated their perception of the council as a whole on multiple dimensions, including trust and representation.

In part II, participants were asked to read four CVs from applicants applying for an internship with the council and indicated their perception of each candidate. Each participant saw four different CVs in a randomized order, which were identical across conditions except for whether the applicant was *privately educated* or *state‐educated*, randomized for each CV (i.e. within participants). This allowed us to investigate (a) whether applicants were perceived differently depending on whether they were privately or state‐educated, (b) whether participants expected privately or state‐educated applicants to be more or less successful in their application and (c) whether these varied by the experimental condition in part I (i.e. whether the council was majority privately or state‐educated, or there was no information about school type). After rating all CVs, participants filled out multiple questionnaires about social and political attitudes, provided socio‐demographic information, were fully debriefed and compensated.

#### Measures

##### Perception of town council

We measured perception of the council with ten items on a scale from 1 (Strongly disagree) to 7 (Strongly agree): *The council is (1) trustworthy, (2) competent, (3) intelligent, (4) in touch with ordinary people, (5) responsible, (6) warm, (7) honest, (8) kind, (9) sociable and (10) down to earth*. The items were inspired by Abele et al. (2016) and Cuddy et al. (2008).

##### Representation by town council

We measured representation with four items on a scale from 1 (Strongly disagree) to 7 (Strongly agree). The items are adapted from Lavi et al. (2021) and capture four types of representation: (1) Descriptive representation (‘The council has representatives that are similar to me in terms of my personal characteristics and background’.), (2) substantive representation (‘The council represents my views’.), (3) symbolic representation (‘The council gives me a sense of belonging and pride in my identity’.), (4) formalistic representation (‘The council will use the authority given to them in a responsible manner for the citizens’.). While the items were designed to capture different facets, they formed a reliable scale (Cronbach's alpha = .85), and therefore, we aggregated them into one representation scale.

##### Perception of internship candidate

Participants' perceptions of the candidates were measured with six items. Participants indicated an (1) overall perception, (2) likelihood of succeeding in the internship, (3) fit with the council, (4) likelihood of getting the internship, (5) suitability to work in politics and (6) appropriate hourly wage (see Appendix S1 for exact phrasing).

##### Individual differences

Belief in school meritocracy (Cronbach's alpha = .87), political orientation (r = .77), institutional trust (Cronbach's alpha = .87) and demographics were measured with the same items as in Study Ia.

### Results

#### Part I

**FIGURE 2:**
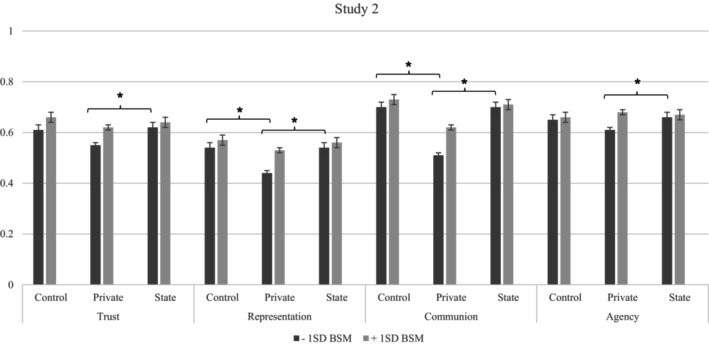
DVs by condition and BSM in Study 2 Part I. *p < .05

We found significant main effects on trust, communion and representation, but not agency. Specifically, in the private‐majority condition, the council was perceived as less trustworthy, less communal and less representative compared with the state‐majority and control (Table S12). There was no significant difference between state‐majority and control. We also tested for moderation by BSM. In the private‐majority condition, individuals with lower BSM perceived the council as less trustworthy (marginally significant), communal and representative than individuals with higher BSM. In the other conditions, BSM did not significantly impact the outcomes (see Figure 2, Tables S13–S16).

#### Part II

Privately educated applicants were overall perceived less positively, but as more likely to receive the internship offer. Further, *success in the internship* and *likelihood to receive the offer* depended on the composition of the council: State‐educated applicants were seen as less likely to receive the offer in the private condition compared with the control and state conditions, whereas the likelihood of privately educated applicants receiving the offer did not differ between the council conditions. State‐educated candidates were perceived to be more likely to be successful in the internship in the council compared with the state. This is in line with the findings of Study 1a and 1b, which show that private education is associated with less group permeability (Table S17). We also found significant three‐way interactions with BSM for all outcomes. The results indicate once again that the difference between private and the two other conditions was reduced for individuals with higher BSM, as compared to individuals with lower BSM (Table S18).

### Discussion

Study 2 shows that the majority representation of privately educated politicians has multiple consequences. First, it decreases trust, communion and feelings of representation. Interestingly, if no information about the education sector is available, trust, representation and communion are higher compared with explicitly mentioning private education. Second, whether a candidate is privately or state‐educated impacts perceptions of how likely it is that they will be offered an internship, even though the education sector was unrelated to the candidate's perceived competence or fit. The results show that perceptions of the chances of a state‐educated applicant receiving a job offer are especially low when the majority of powerful positions are occupied by the privately educated. This implies that, in line with the previous studies, individuals perceive that privileged groups prefer members of their own group (i.e. ingroup bias). Finally, and again in line with our prior studies, these effects are more pronounced among individuals who do not believe that schooling is meritocratic.

## STUDY 3

Study 2 found that political institutions in which the privately educated form the majority are perceived as less trustworthy than institutions in which the state educated form the majority. Study 3 extends these findings by exploring whether the (dis)proportional representation of privately educated individuals in political institutions affects trust in those institutions, and how much those institutions are perceived as representing society. We expected trust and representation to be lower when the proportion of privately educated individuals was higher in the political institution than in the general population (overrepresented) compared with when the proportions were matched, or when the privately educated were underrepresented in the institution (H1a & H2a). We expected trust and representation to be higher when the privately educated were underrepresented compared with matched or overrepresented (H1b & H2b). We also explored whether perceived deservingness—a key aspect of meritocratic ideology—could explain the effects.

### Method

#### Participants and experimental design

The final sample consisted of 382 U.K. participants (197 women, 179 men, 5 other/not specified; *M*_Age_ = 41.74, SD_Age_ = 13.90) who were recruited via Prolific Academics. Participants were randomly allocated to one of three conditions. In each condition, participants learned about a fictional society called Bimboola and its education sector, that is, a separation between costly privately‐funded schools and free state‐funded schools (Sprong et al., 2019). Additionally, participants learned that 10% of children in Bimboola are privately educated. Next, participants learned about the proportion of privately educated politicians in Bimboola's parliament. In the underrepresentation condition (i.e. UC; *n* = 127), *0 out of 100* parliament members were privately educated. In the matched condition (i.e. MC; *n* = 129), *10 out of 100* parliament members were privately educated. In the overrepresentation condition (OC; *n* = 126*), 35 out of 100* parliament members were privately educated. Participants indicated their perception of the politicians and of Bimboola's parliament on multiple dimensions, including trust and representation.

#### Measures

##### Representation by Bimboola's parliament

Participants indicated the extent to which they perceive Bimboola's parliament representative of Bimboola's society using three items on a scale from 1 (Not at all) to 9 (Very much). An example item reads: *To what extent do you think the parliament is representative of Bimboola's society?* Internal consistency was high (Cronbach's alpha = .90).

##### Trust in Bimboola's government

Participants indicated the extent to which they trust Bimboola's parliament using four items on a scale from 1 (Not at all) to 9 (Very much). An example item reads: *To what extent do you think the Bimboola's parliament is trustworthy?* Internal consistency was high (Cronbach's alpha = .92).

##### Deservingness of political position

Participants indicated the extent to which politicians in Bimboola's parliament deserve their position on a scale from 1 (Not at all) to 9 (Very much). The item reads: *To what extent do you think politicians in Bimboola's parliament deserve their position?*

##### Individual differences

Belief in school meritocracy (Cronbach's alpha = .87), political orientation (*r* = .70), institutional trust (Cronbach's alpha = .87) and demographics were measured with the same items as in Study 1a.

### Results

We ran two ANOVAs with trust and representation, respectively, as dependent variables and included age, gender, own school type and political orientation as control variables. We used two planned contrasts to compare (1) OC against UC and MC; and (2) UC against the MC. The results indicate that, in the OC, trust and representation were significantly lower compared with UC and MC (supporting H1a and H2a), but, in UC, trust and feelings of representation were not significantly higher compared with MC (rejecting H1b and H2b) (Table S20). We again investigated whether meritocratic beliefs moderated the effects, but contrary to other studies, BMS did not moderate the effects (Table S21).

We also explored whether deservingness serves as a mediator. We found partial mediation for both trust and representation. Focusing first on the association between condition and deservingness, we found that perceived deservingness was higher in the MC and UC compared with the OC. In turn, deservingness positively predicted trust and representation. After accounting for deservingness, the dummy for OC vs. UC became non‐significant for trust and was reduced for representation, while the dummy for OC vs. MC was significantly reduced for both trust and representation. This indicates that deservingness does indeed mediate the relationship, albeit only partially (Table 2).

**TABLE 2:** Deservingness as mediator of trust and representation.

Model 1: Deservingness as meditator of trust		
	Deservingness	Trust
Over versus matched (a1 path)	0.95 (0.20)***	
Over versus under (a2 path)	1.50 (0.20)***	
Deservingness (b path)		0.74 (0.03)***
Over versus matched (c1 path)		0.95 (0.19)***
Over versus under (c2 path)		1.22 (0.19)***
Over versus matched (c’1 path)		0.25 (0.18)*
Over versus under (c’2 path)		0.11 (0.13)

Model 2: Deservingness as meditator of representation		
	Deservingness	Representation
Over versus matched (a1 path)	0.95 (0.20)***	
Over versus under (a2 path)	1.50 (0.20)***	
Deservingness (b path)		0.74 (0.05)***
Over versus matched (c1 path)		2.30 (0.23)***
Over versus under (c2 path)		2.21 (0.23)***
Over versus matched (c’1 path)		1.60 (0.18)***
Over versus under (c’2 path)		1.10 (0.19)***

*Note*: *p < .05, **p < .001.

### Discussion

The findings in Study 3 demonstrate that the overrepresentation of elite (i.e. privately educated) individuals in the political system of a fictional society negatively impacts the perception of the government. The government is perceived as more trustworthy and representative if the proportion of privately educated parliamentarians matches or is lower than the proportion of privately educated individuals in the general population. Additionally, when the privately educated are overrepresented, politicians are perceived as less deserving of their position compared with when the privately educated are underrepresented. The results further indicate that perceived deservingness is one potential process underlying distrust and perceived lack of representation. That is, when elite groups are overrepresented in powerful positions, members of that group are perceived as less deserving of their position and are therefore less trusted.

## STUDY 4

Our previous results indicate that holding meritocratic beliefs about school tends to reduce the differences in perception between privately and state‐educated politicians. We did not find this moderation only in Study 3, when the items assessing merit were keyed to U.K. society rather than to Bimboolean society, which was the focus of the manipulation. Study 4 builds on these findings by directly manipulating merit. Participants read a description of either a state‐educated politician, a privately educated politician or a privately educated politician who is explicitly described as working hard throughout school to increase their perceived merit. We expect that political trust (H1), representation (H2) and perceived deservingness (H3) will be lower in the *private school condition* than in the *state‐school condition*, unless private school is associated with a high merit (*merit condition*).

### Method

#### Participants and experimental design

The final sample consisted of 614 U.K. participants (290 women, 321 man, 3 other; Age_M_ = 41.8 years, Age_SD_ = 13.85). Participants were randomly assigned to one of three conditions. In each condition, participants read a vignette about a fictional politician. In the state‐school condition (SC; *n* = 205), the politician attended a state school. In the private school condition (PC; *n* = 210), the politician attended a private school. In the merit condition (PMC; *n* = 207), the politician attended a private school and worked hard throughout school (to increase their merit and deservingness). All other information was held stable across conditions. After reading the vignette, participants indicated their perceptions of the politician regarding trust and deservingness, as well as how representative of their personal views, of U.K. society and of U.K. politicians they were.

#### Measures

##### Perception of merit/luck

Participants indicated their perception of the extent to which the politician obtained his political position due to merit or luck with eight items on a scale from 1 (not at all) to 5 (extremely). The items were constructed for this research and are displayed in Appendix S1. Reliability was high (i.e. Cronbach's alpha = .84), and the items were aggregated into a mean score.

##### Deservingness

Participants indicated their perception of the extent to which the politician deserves his political position with one item on a scale from 1 (Not at all) to 5 (Extremely). The item reads: *David Smith deserves his political position*.

##### Communion and agency

Participants' perceptions of the politician were assessed with eight items on a scale from 1 (Not at all) to 5 (Extremely). The items read: David Smith is (1) *out of touch with ordinary people*, (2) *capable*, (3) *moral*, (4) *honest*, (5) *well‐connected*, (6) *has integrity*, (7) *corrupt* and (8) *opportunistic*.

##### Trust

Participants indicated the extent to which they trust David Smith using three items on a scale from 1 (Not at all) to 11 (Completely). An example item reads: ‘To what extent do you trust your local councilor David Smith?’ Internal consistency was high (Cronbach's alpha = .96), and the items were aggregated to a composite score.

##### Representation

Participants indicated the extent to which they feel represented by David Smith using three items on a scale from 1 (Not at all) to 5 (Completely). The items measured the extent to which David Smith's views are representative of (1) them personally, (2) English society and (3) English politicians. Personal and societal representation correlated highly (*r* = .67), but personal representation was unrelated to representation of politicians (*r* = .02) and only weakly related to societal representation (*r* = .15).

##### Individual differences

Belief in school meritocracy (Cronbach's alpha = .86), political orientation (*r* = .75), institutional trust (Cronbach's alpha = .86) and demographics were measured with the same items as in Study 1a.

### Results

**FIGURE 3:**
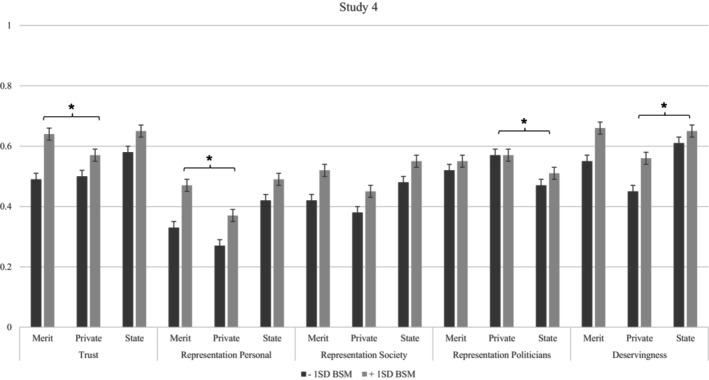
DV's by condition and BSM in Study 4. *p < .05

We found that the politician was perceived as more trustworthy, more deserving and more representative of personal views and society, but less representative of politicians in general, in the SC compared with the PMC and PC. The PMC and PC only differed in perceived deservingness, with perceived deservingness higher in the PMC condition (Table S23). We also found significant interactions with BSM for all outcomes. The results indicate that individuals with low BSM perceived the politician as less trustworthy, less deserving and less representative of themselves and society than individuals with high BSM in the PMC and in the PC, but not in the SC (see Figure 3, Table S24).

We explored whether deservingness serves as a mediator between condition and trust/representation. We found partial mediation for both trust and representation. That is, perceived deservingness was higher in the PMC and SC, compared with the PC, and in turn, deservingness positively predicts trust and representation. After accounting for deservingness, the direct effect in the dummy for private vs. merit became non‐significant for representation, but there was neither a direct nor an indirect effect on trust. The direct effect for the dummy for private vs. state was significantly reduced for both trust and representation. This indicates that deservingness does indeed mediate the relationship, albeit only partially (see Table 3).

**TABLE 3:** Deservingness as mediator of trust and personal representation.

Model 1: Deservingness as meditator of trust		
	Deservingness	Trust
Private versus merit (a1 path)	0.34 (0.08)***	
Private versus state (a2 path)	0.50 (0.08)***	
Deservingness (b path)		1.43 (0.08)***
Private versus merit (c1 path)		0.32 (0.20)
Private versus state (c2 path)		1.14 (0.19)***
Private versus merit (c’1 path)		−0.17 (0.17)
Private versus state (c’2 path)		0.43 (0.17)*

Model 2: Deservingness as meditator of personal representation		
	Deservingness	Representation
Private versus merit (a1 path)	0.34 (0.08)***	
Private versus state (a2 path)	0.50 (0.08)***	
Deservingness (b path)		0.56 (0.04)***
Private versus merit (c1 path)		0.21 (0.09)*
Private versus state (c2 path)		0.59 (0.09)***
Private versus merit (c’1 path)		0.01 (0.08)
Private versus state (c’2 path)		0.29 (0.08)***

*Note*: *p < .05, **p < .001.

### Discussion

The results of Study 3 show that state‐educated politicians are perceived as more trustworthy and representative than privately educated politicians, even when merit is made explicitly salient. However, merit does increase trust and representation to some extent compared with no explicit mention of merit. Further, the perception depends on individual differences in BSM. For those with higher BSM, perceptions of the politician did not vary by condition, whereas for those with lower BSM, condition did influence their perceptions. Mediation analysis showed that perceived deservingness is one process underlying the relationship between school type, merit information and trust/representation.

## GENERAL DISCUSSION

In 2022, trust in politicians in the U.K. was at an all‐time low, dropping 9 percent within just 18 months (Quilter‐Pinner, 2022). Falls of similar scales have previously taken 7 and 42 years. This steady decline of political trust in many democracies is often accompanied by a growing sense of political alienation and rising populist forces. This poses a threat as citizens trust in their elected representatives, and the system is essential to uphold democratic governance. To combat these developments and regain trust, it is first crucial to understand which factors contribute to the erosion of political trust. Our research examined one potential factor: the overrepresentation of elites in politics. We found that political trust is corroded when politicians and political institutions are perceived as being dominated by an elite group. Conceptualizing the education sector as a proxy for social class, our results show that the overrepresentation of privileged politicians—especially when such privilege is considered to be undeserved or to reflect an unmeritorious system—can undermine political trust.

Our results demonstrate that trust in politicians is eroded when politicians are perceived to be privileged through having received private rather than state education, and that trust in political institutions is eroded when they have a preponderance of privately educated members. We found similar effects for feeling represented by politicians and political institutions. The effects were consistently stronger for individuals who tended to believe that school is an unmeritocratic system, a finding that persisted when political orientation and education section was accounted for, suggesting that belief in school meritocracy does not simply capture a more conservative worldview.

The current research is set in the U.K. context in which the separation between private and state schools has a long‐standing tradition and is indeed a highly debated topic. For example, the U.K. Labour government recently passed a bill abolishing the tax exemption of private schools. Attending expensive private schools is more strongly associated with class boundaries, and private schools remain a space almost exclusively reserved for privileged groups. Traditionally high‐status professions like law, politics and journalism recruit individuals almost entirely from privileged class backgrounds who attend private schools (Milburn, 2009, 2012). Furthermore, U.K. media outlets frequently report on the proportion of private‐ and state‐school educated politicians among the cabinet members after an election.^2^,^3^ These examples highlight the societal importance of the topic in the U.K. context, yet also pose the question of the relevance outside the U.K. In other contexts, the differences between different education sectors are less stark, and thus, the education sector might be less important for the evaluation of politicians and political institutions. However, while the definitional characteristics of elite groups might vary across contexts, the consequence of their overrepresentation and the processes that such overrepresentations ignite might well be similar. This is especially likely to be the case if the indicators of high class relate to a time of socialization, strongly evoke associations of wealth and involve what are perceived as tight‐knit and closed‐ranks elite groups, as does the education sector in the U.K. While different and context‐specific indicators of higher class may be needed in different contexts, we expect the processes and consequences of such elite overrepresentation to be the same in other contexts.

This poses the question of how best to measure social class. Manstead (2018) described social class as a qualitative group identity that is rooted in shared history and collective consciousness among groups, while Ostrove and Cole (2003) highlighted that social class comprises economic components and social components. Others have argued that social class can be distinguished from socioeconomic status by its group‐based nature and its relevance to socialization processes (Easterbrook et al., 2023). Yet, most investigations into social class rely on objective economic measures like income, education level or occupation (see Antonoplis, 2023; Easterbrook et al., 2023; Rubin, 2012 for reviews), which mostly ignore socialization and social resources. We responded to calls for more theoretically informed measures of social class (Antonoplis, 2023) and suggest that the education sector is an excellent measure of elite social class within the U.K. context that directly relates to a period of socialization, to membership of a tight‐knit group with closed ranks, and wealth. In other contexts, different measures may be more appropriate (such as, for example, sorority‐membership in the Netherlands and Germany, or elite college attendance in the U.S.). Measures could be extended to capture access to tangible and intangible resources during childhood and adolescence, including aspects like the education sector, health care sector (i.e. access to private health care) and social networks.

The consequences of elite groups being proportionally overrepresented in the political sphere can be severe and potentially impact classes differently. Firstly, for high‐status individuals and among the elite, such overrepresentation can ignite identity processes that in turn uphold the status quo and entrench privilege and inequality through biased political decision making. For example, in the Netherlands, where around 40% of politicians are from upper‐class backgrounds, ingroup biases among elites may be at play: Schakel (2021) found that political outcomes such as tax policies are biased towards the rich (Bartels, 2016; Gilens, 2012). Second, for low‐status and non‐elite members of society, elite overrepresentation might (unconsciously) communicate that politics is not a space for them. Thus, it may systematically alienate certain groups from politics by reducing the perceived relevance of politics to their identities and thus their political engagement (Noordzij et al., 2021). Our work further demonstrates that the overrepresentation of elites in politics corrodes trust and feelings of representation and adds to a body of work suggesting that proportional representation may help to engage people with politics and foster political trust. Future research could build on our findings and explore directly how representation can impact political engagement and the aspirations of individuals to go into politics.

Our work highlights that the education sector impacts trust on an individual level and a group level. Study 1a and Study 4 show that privately educated politicians are perceived as less trustworthy. These findings are in line with social‐class stereotype literature showing that high SES professions like lawyers and politicians can evoke distrust due to fear of exploitation (Durante & Fiske, 2017; Fiske & Durante, 2014). In the political area, this might indicate that people perceive these politicians as less likely to advocate for their concerns. In Study 2 and Study 3, we find that groups which include privately educated politicians are perceived as less trustworthy, even when privately educated politicians are numerically in the minority. This implies that the lower number does not signify less power of the group. This might be rooted in social‐class stereotypes. We do find that privately educated politicians are seen as more manipulative, more well‐spoken and influential; thus, participants might attribute more power to them in the political area. Further, research on superordinate identities and attitude generalization shows that people generalize individual's characteristics onto the group, especially when social categories are salient (Brown & Hewstone, 2005). The ingroup project model (Wenzel et al., 2007) postulates that group members project their own positive ingroup characteristics onto the superordinate identity. In the current studies, it seems like people generalize the negative associations evoked by the privately educated to the whole group of politicians. Potentially, the privately educated are seen as more prototypical of politicians. Indeed, in Study 1b, privately educated politicians belong to the same cluster, and in Study 4, participants indicated that privately educated politicians are most representative of politicians in general. Future research could investigate these findings in more detail.

Prior research focusing on politicians' education level found that people tend to prefer higher‐educated politicians (van Noord et al., 2023) compared with lower‐educated politicians. Our findings show that not only the level of education but also the education sector impact perceptions of politicians. Unlike the findings of van Noord et al. (2023), we do not find that those who get the ‘best’ education (i.e. in private schools, which have more resources per pupil) are preferred. Importantly, education level and education sector are not equivalent. Our cluster analysis in Study 1b demonstrates this empirically. That is, higher‐educated and privately educated belong to different clusters. Higher‐educated individuals were characterized by high agency and high communion, while privately educated individuals were characterized by high agency and low communion. Importantly, lower‐educated and state‐educated individuals also fell into different clusters. Further, participants level of education and education sector were unrelated. Combined, these findings show that education level and education sector are different aspects. Education level is often interpreted as a deserved status obtained through meritocratic means. By contrast, as our findings suggest, private education violates meritocratic principles and is perceived as an undeserved privilege. This indicates that higher‐educated individuals might be preferred because they are perceived as deserving of their status. This interpretation is in line with our findings that belief in school meritocracy reduces the perceived distinction between privately and state‐educated politicians. As long as people believe that status and success depend on merit, inequalities are perceived as justified, and unequal representation is accepted (Fisher et al., 2017). Recognizing structural barriers faced by those of lower SES, which violate the principles of meritocracy, may therefore be a potential avenue through which to increase support for proportional representation.

The research focuses on one specific moderator: belief in school meritocracy. The belief assumes that schools provide a level playing field for everyone, regardless of external factors like socioeconomic background, and that everyone has equal chances to succeed. As we manipulated the education sector, a belief associated with the schooling system seemed like the sensible choice. However, we acknowledge that merit beliefs might be embedded within a larger belief system characterized by system‐justifying attitudes, including political orientation and neoliberal mindsets. Our results in the SOM show that merit beliefs impact perception above political orientation, indicating that it is not just an effect of political mindset. Further, we did not measure participants identification with the education sector. While additional analyses using the education sector of participants as a moderator to test for potential in‐ versus outgroup effects were not significant, the role of identity processes in our studies remains unclear. Nonetheless, future studies should further investigate other moderators and test group processes by manipulating identity salience.

## CONCLUSION

Our research highlights the detrimental effects that the overrepresentation of privilege in politics can have on political trust. Trust is an essential ingredient to successful democratic governance. Only if citizens believe that those elected act in their best interest, do democracies have a chance to combat anti‐democratic and populist forces.

## AUTHOR CONTRIBUTIONS

**Rebekka Kesberg:** Conceptualization; investigation; writing – original draft; methodology; formal analysis; writing – review and editing. **Matthew J. Easterbrook:** Conceptualization; investigation; writing – original draft; methodology; formal analysis; writing – review and editing; conceptualization; funding acquisition; writing – review and editing; supervision; validation.

## FUNDING INFORMATION

This work is financially supported by the NORFACE Joint Research Programme on Democratic Governance in a Turbulent Age and co‐funded by the Economic and Social Research Council, U.K. and the European Commission through Horizon 2020 under grant agreement No 822166.

## CONFLICT OF INTEREST STATEMENT

We do not have any potential competing interests.

## Supporting information


Appendix S1.

## Data Availability

Preregistration, data and syntax are available on OSF (https://osf.io/etsxj/?view_only=e465cbbdae584eab9cfea762f4749ca8).

## Footnotes

^1^
Fee‐paying schools are commonly known as ‘public schools’ in the United Kingdom. As the attribute ‘public’ is confusing, we here use the term fee‐paying schools or private school instead.

^2^

https://www.theguardian.com/education/2019/jul/25/two‐thirds‐of‐boris‐johnsons‐cabinet‐went‐to‐private‐schools.

^3^

https://www.theguardian.com/politics/2016/jul/14/theresa‐mays‐cabinet‐state‐educated‐but‐mostly‐male.

## References

[bjso12885-bib-0001] Aaldering, L. , & Vliegenthart, R. (2016). Political leaders and the media. Can we measure political leadership images in newspapers using computer‐assisted content analysis? Quality and Quantity, 50(5), 1871–1905. 10.1007/s11135-015-0242-9 27563155 PMC4981631

[bjso12885-bib-0002] Abele, A. E. , Hauke, N. , Peters, K. , Louvet, E. , Szymkow, A. , & Duan, Y. (2016). Facets of the fundamental content dimensions: Agency with competence and assertiveness‐communion with warmth and morality. Frontiers in Psychology, 7, 1810. 10.3389/fpsyg.2016.01810 27920737 PMC5118442

[bjso12885-bib-0003] Abelson, R. P. , Kinder, D. R. , Peters, M. D. , & Fiske, S. T. (1982). Affective and semantic components in political person perception. Journal of Personality and Social Psychology, 42(4), 619–630. 10.1037/0022-3514.42.4.619

[bjso12885-bib-0004] Ahmed, M. A. O. , Zhang, J. , Fouad, A. S. , Mousa, K. , & Nour, H. M. (2024). The dark side of leadership: How toxic leadership fuels counterproductive work behaviors through organizational cynicism and injustice. Sustainability, 17(1), 105. 10.3390/su17010105

[bjso12885-bib-0005] Antonoplis, S. (2023). Studying socioeconomic status: Conceptual problems and an alternative path forward. Perspectives on Psychological Science, 18(2), 275–292. 10.1177/17456916221093615 35981108 PMC10018062

[bjso12885-bib-0006] Bartels, L. M. (2016). Unequal democracy: The political economy of the new gilded age. Russell Sage Foundation.

[bjso12885-bib-0007] Batruch, A. , Autin, F. , & Butera, F. (2019). The paradoxical role of meritocratic selection in the perpetuation of social inequalities at school. In J. Jetten & K. Peters (Eds.), The social psychology of inequality. Springer.

[bjso12885-bib-0008] Batruch, A. , Jetten, J. , Van de Werfhorst, H. , Darnon, C. , & Butera, F. (2023). Belief in school meritocracy and the legitimization of social and income inequality. Social Psychological and Personality Science, 14(5), 621–635. 10.1177/19485506221111017 37223669 PMC10201081

[bjso12885-bib-0009] Blais, A. , Singh, S. , & Dumitrescu, D. (2014). Elections and democracy (Thomassen, J., Ed.). Oxford University Press. 10.1093/acprof:oso/9780198716334.001.0001

[bjso12885-bib-0010] Bourdieu, P. , & Passeron, J. C. (1977). Reproduction in education, society, and culture. Sage in association with Theory, Culture & Society, Dept. of Administrative and Social Studies, Teesside Polytechnic.

[bjso12885-bib-0011] Bovens, M. , & Wille, A. (2017). Diploma democracy: The rise of political meritocracy. Oxford University Press.

[bjso12885-bib-0012] Brown, R. , & Hewstone, M. (2005). An integrative theory of intergroup contact. Advances in Experimental Social Psychology, 37, 255–343. 10.1016/s0065-2601(05)37005-5

[bjso12885-bib-0013] Crawford, M. T. , Sherman, S. J. , & Hamilton, D. L. (2002). Perceived entitativity, stereotype formation, and the interchangeability of group members. Journal of Personality and Social Psychology, 83(5), 1076–1094. 10.1037/0022-3514.83.5.1076 12416913

[bjso12885-bib-0014] Cuddy, A. J. C. , Fiske, S. T. , & Glick, P. (2008). Warmth and competence as universal dimensions of social perception: The stereotype content model and the BIAS map. In Advances in experimental social psychology (Vol. 40, pp. 61–149). Academic Press. 10.1016/S0065-2601(07)00002-0

[bjso12885-bib-0015] Darnon, C. , Wiederkehr, V. , Dompnier, B. , & Martinot, D. (2018). ‘Where there is a will, there is a way’: Belief in school meritocracy and the social‐class achievement gap. British Journal of Social Psychology, 57(1), 250–262. 10.1111/bjso.12214 28892168

[bjso12885-bib-0016] Dasgupta, N. , Banaji, M. R. , & Abelson, R. P. (1999). Group Entitativity and group perception: Associations between physical features and psychological judgement. Journal of Personality and Social Psychology, 77(5), 991–1003. 10573876 10.1037//0022-3514.77.5.991

[bjso12885-bib-0017] Disch, L. (2011). Toward a mobilization conception of democratic representation. American Political Science Review, 105(1), 100–114. 10.1017/S0003055410000602

[bjso12885-bib-0018] Dovi, S. (2018). Political Representation. In E. N. Zalta (Ed.), The Stanford encyclopedia of philosophy. Metaphysics Research Lab, Stanford University.

[bjso12885-bib-0019] Dunn, K. (2015). Voice, representation and trust in parliament. Acta Politica, 50(2), 171–192. 10.1057/ap.2014.15

[bjso12885-bib-0020] Durante, F. , & Fiske, S. T. (2017). How social‐class stereotypes maintain inequality. In Current opinion in psychology (Vol. 18, pp. 43–48). Elsevier B.V. 10.1016/j.copsyc.2017.07.033 29221511 PMC6020691

[bjso12885-bib-0021] Easterbrook, M. J. , Kuppens, T. , & Grigoryan, L. (2023). Introduction to the special issue: nuances of social class and socioeconomic status (Introducción a este monográfico: los matices del concepto de clase social y del nivel socioeconómico). International Journal of Social Psychology, 38(3), 493–507. 10.1080/02134748.2023.2239577

[bjso12885-bib-0022] Easterbrook, M. J. , Kuppens, T. , & Manstead, A. S. R. (2020). Socioeconomic status and the structure of the self‐concept. British Journal of Social Psychology, 59(1), 66–86. 10.1111/bjso.12334 31175690

[bjso12885-bib-0023] Echebarria Echabe, A. (2014). System‐justifying beliefs and political disaffection. Journal of Applied Social Psychology, 44(3), 234–240. 10.1111/jasp.12218

[bjso12885-bib-0024] Fisher, O. , O'Donnell, S. C. , & Oyserman, D. (2017). Social class and identity‐based motivation. Current Opinion in Psychology, 18, 61–66. 10.1016/j.copsyc.2017.07.035 28826006

[bjso12885-bib-0025] Fiske, S. T. , & Durante, F. (2014). Never trust a politician? Collective distrust, relational accountability, and voter response. In J.‐W. Van Prooijen & P. A. M. Van Lange (Eds.), Power, politics, and paranoia: Why people are suspicious of their leaders (1st ed., pp. 91–105). Cambridge University Press.

[bjso12885-bib-0026] Gandolfi, F. , & Stone, S. (2022). Toxic leadership: Behaviors, characteristics, and consequences. Journal of Management Research, 22(1), 19–27.

[bjso12885-bib-0027] Gilens, M. (2012). Affluence and influence. Princeton University Press. 10.1515/9781400844821

[bjso12885-bib-0028] Grigoryan, L. , Jones, B. H. , Cohrs, J. C. , Boehnke, K. , & Easterbrook, M. J. (2023). Differentiating between belief‐indicative and status‐indicative groups improves predictions of intergroup attitudes. Personality and Social Psychology Bulletin, 49(7), 1097–1112. 10.1177/01461672221092852 35596556

[bjso12885-bib-0029] Jones, O. (2015). The establishment: And how they get away with it. Melville House.

[bjso12885-bib-0030] Kuppens, T. , Spears, R. , Manstead, A. S. R. , Spruyt, B. , & Easterbrook, M. J. (2018). Educationism and the irony of meritocracy: Negative attitudes of higher educated people towards the less educated. Journal of Experimental Social Psychology, 76, 429–447. 10.1016/j.jesp.2017.11.001

[bjso12885-bib-0031] Lavi, L. , Treger, C. , Rivlin ‐Angert, N. , Sheafer, T. , Waismel Manor, I. , Shenhav, S. , Harsgor, L. , & Shamir, M. (2021). The Pitkinian public: Representation in the eyes of citizens . https://ssrn.com/abstract=3837497

[bjso12885-bib-0032] Ledgerwood, A. , Mandisodza, A. N. , Jost, J. T. , & Pohl, M. J. (2011). Working for the system: Motivated defense of meritocratic beliefs. Social Cognition, 29(3), 322–340. 10.1521/soco.2011.29.3.322

[bjso12885-bib-0033] Lickel, B. , Hamilton, D. L. , Lewis, A. , Sherman, S. J. , Wieczorkowska, G. , & Uhles, A. N. (2000). Varieties of groups and the perception of group entitativity. Journal of Personality and Social Psychology, 78(2), 223–245. 10.1037/0022-3514.78.2.223 10707331

[bjso12885-bib-0034] Mansbridge, J. , & Macedo, S. (2019). Populism and democratic theory. Annual Review of Law and Social Science, 15(1), 59–77. 10.1146/annurev-lawsocsci-101518-042843

[bjso12885-bib-0035] Manstead, A. S. R. (2018). The psychology of social class: How socioeconomic status impacts thought, feelings, and behaviour. British Journal of Social Psychology, 57(2), 267–291. 10.1111/bjso.12251 29492984 PMC5901394

[bjso12885-bib-0036] Milburn, A. (2009). Unleashing aspiration: the final report of the panel on fair access to the professions . Cabinet Office.

[bjso12885-bib-0037] Milburn, A. (2012). Fair access to professional careers. The independent reviewer on social mobility and child poverty . Cabinet Office. https://hdl.voced.edu.au/10707/326959

[bjso12885-bib-0038] Noordzij, K. , de Koster, W. , & van der Waal, J. (2021). “They don't know what it's like to be at the bottom”: Exploring the role of perceived cultural distance in less‐educated citizens' discontent with politicians. British Journal of Sociology, 72(3), 566–579. 10.1111/1468-4446.12800 33368242 PMC8359444

[bjso12885-bib-0039] Noordzij, K. , de Koster, W. , & van der Waal, J. (2023). Explaining the educational gradient in trust in politicians: A video‐vignette survey experiment. West European Politics, 47(4), 784–812. 10.1080/01402382.2023.2250163

[bjso12885-bib-0040] Noury, A. , & Roland, G. (2020). Identity politics and populism in Europe. Annual Review of Political Science, 2020(23), 421–439. 10.1146/annurev-polisci-050718

[bjso12885-bib-0041] Ostrove, J. M. , & Cole, E. R. (2003). Privileging class: Toward a critical psychology of social class in the context of education. Journal of Social Issues, 59(4), 677–692. 10.1046/j.0022-4537.2003.00084.x

[bjso12885-bib-0042] Pettit, P. (2009). Varieties of public representation. In I. Shapiro , S. C. Stokes , E. W. Wood , & A. S. Kirshner (Eds.), Political representation (pp. 61–89). Cambridge University Press.

[bjso12885-bib-0043] Quilter‐Pinner, H. (2022). Freefall: How a year of chaos has undermined trust in politics . https://www.ippr.org/articles/freefall‐how‐a‐year‐of‐chaos‐has‐undermined‐trust‐in‐politics

[bjso12885-bib-0058] R Core Team (2024). R: A Language and Environment for Statistical Computing. R Foundation for Statistical Computing, Vienna, Austria.

[bjso12885-bib-0044] Rawls, J. (2016). A theory of justice. In L. May (Ed.), Applied ethics (6th ed., pp. 21–29). Routledge.

[bjso12885-bib-0045] Rubin, M. (2012). Social class differences in social integration among students in higher education: A meta‐analysis and recommendations for future research. Journal of Diversity in Higher Education, 5(1), 22–38. 10.1037/a0026162

[bjso12885-bib-0046] Schakel, W. (2021). Unequal policy responsiveness in The Netherlands. Socio‐Economic Review, 19(1), 37–57. 10.1093/ser/mwz018

[bjso12885-bib-0047] Sibietaa, L. (2023). Tax, private school fees and state school spending . www.nuffieldfoundation.org

[bjso12885-bib-0048] Skinner, Q. (2005). Hobbes on representation. European Journal of Philosophy, 13(2), 155–184. 10.1111/j.0966-8373.2005.00226.x

[bjso12885-bib-0049] Sprong, S. , Jetten, J. , Wang, Z. , Peters, K. , Mols, F. , Verkuyten, M. , Bastian, B. , Ariyanto, A. , Autin, F. , Ayub, N. , Badea, C. , Besta, T. , Butera, F. , Costa‐Lopes, R. , Cui, L. , Fantini, C. , Finchilescu, G. , Gaertner, L. , Gollwitzer, M. , … Wohl, M. J. A. (2019). “Our country needs a strong leader right now”: Economic inequality enhances the wish for a strong leader. Psychological Science, 30(11), 1625–1637. 10.1177/0956797619875472 31566081

[bjso12885-bib-0050] Spruyt, B. , Caluwaerts, D. , Darnon, C. , Easterbrook, M. , Kavadias, L. , Kesberg, R. , Kuppens, T. , Manstead, A. , Smets, L. , & Van Noord, J. (2025). Backlash among the dominant? Assessing support for elitism in four European countries. Political Psychology, 1–20. 10.1111/pops.70010

[bjso12885-bib-0051] Sutton Trust , & Social Mobility Commission . (2019). Elitist Britain 2019. The educational backgrounds of Britain's leading people . Sutton Trust.

[bjso12885-bib-0052] Van Doesum, N. J. , Tybur, J. M. , & Van Lange, P. A. M. (2017). Class impressions: Higher social class elicits lower prosociality. Journal of Experimental Social Psychology, 68, 11–20. 10.1016/j.jesp.2016.06.001

[bjso12885-bib-0053] van Noord, J. , Kuppens, T. , Spruyt, B. , & Spears, R. (2023). When and why people prefer higher educated politicians: Ingroup bias, deference, and resistance. Personality and Social Psychology Bulletin, 49(4), 585–599. 10.1177/01461672221077794 35191783 PMC9989228

[bjso12885-bib-0054] Wenzel, M. , Mummendey, A. , & Waldzus, S. (2007). Superordinate identities and intergroup conflict: The ingroup projection model. European Review of Social Psychology, 18(1), 331–372. 10.1080/10463280701728302

[bjso12885-bib-0055] Wessels, B. (2011). Performance and deficits of present‐day representation (Alonso, S., Keane, J., & Merkel, W., Eds.). Cambridge University Press. 10.1017/CBO9780511770883

[bjso12885-bib-0056] Wiederkehr, V. , Bonnot, V. , Krauth‐Gruber, S. , & Darnon, C. (2015). Belief in school meritocracy as a system‐justifying tool for low status students. Frontiers in Psychology, 6, 1053. 10.3389/fpsyg.2015.01053 26283991 PMC4519673

[bjso12885-bib-0057] Zhai, Y. , Wu, Q. , & Lu, Y. (2023). Cultural values, system justification, meritocratic beliefs, and evaluations of governments' performance in handling the COVID‐19 crisis. Asian Journal of Social Psychology, 26(3), 374–384. 10.1111/ajsp.12565

