# Supplementary material for: (Not) one of us: The overrepresentation of elites in politics erodes political trust
Source: Br J Soc Psychol. 2025 Apr 5;64(2):e12885. doi: 10.1111/bjso.12885 (PMC11971726; doi:10.1111/bjso.12885)
Supplement: Supplementary file 1 — Appendix S1. [file BJSO-64-0-s001.docx]

Contents

[Material 4](#_Toc188353904)

[Study 1a 4](#_Toc188353905)

[Study 1b 6](#_Toc188353906)

[Study 2 6](#_Toc188353907)

[Study 3 11](#_Toc188353908)

[Study 4 12](#_Toc188353909)

[Individual Differences Measures in all Studies 14](#_Toc188353910)

[Additional Analysis 20](#_Toc188353911)

[Study 1a 20](#_Toc188353912)

[Table S 1. Correlation Study 1a. 20](#_Toc188353913)

[Table S 2. Main effect condition or Communion, Agency, Toxicity, and Trust. 21](#_Toc188353914)

[Table S 3*.* IA Condition and Belief in School Meritocracy for Communion, Agency, Toxicity, and Trust. 22](#_Toc188353915)

[Study 1b 23](#_Toc188353916)

[Table S 4. Correlation Study 1b*.* 25](#_Toc188353917)

[Figure S2. DV’s by BSM and Social Group (Private Educated vs. State Educated) in Study 1b. 26](#_Toc188353918)

[Table S 5. Communion and Agency (privately educated as reference group) 27](#_Toc188353919)

[Table S 6. Group Entitativity, Permeability and Status (privately educated as reference group). 28](#_Toc188353920)

[Table S 7. Interdependence, Influence and Conflict (privately educated as reference group). 29](#_Toc188353921)

[Table S 8. Communion and Agency by group and BSM (privately educated as reference group). 30](#_Toc188353922)

[Table S 9. Communion and Agency by group and BSM (privately educated as reference group). 32](#_Toc188353923)

[Table S 10. Interdependence, influence, and conflict by group and BSM (privately educated as reference group). 34](#_Toc188353924)

[Study 2 36](#_Toc188353925)

[Table S 11. Correlations Study 2. 36](#_Toc188353926)

[Table S 12. Fixed-Effects ANOVA results. 37](#_Toc188353927)

[Table S 13. IA Condition and BSM on Trust. 38](#_Toc188353928)

[Table S 14. IA Condition and BSM on Representation. 39](#_Toc188353929)

[Table S 15. IA Condition and BSM on Agency. 40](#_Toc188353930)

[Table S 16. IA Condition and BSM on Communion. 41](#_Toc188353931)

[Table S 17. Interaction CV condition and Council Condition. 42](#_Toc188353932)

[Table S 18. Interaction CV condition, Council Condition and BSM. 44](#_Toc188353933)

[Study 3 46](#_Toc188353934)

[Table S 19. Correlations Study 3. 46](#_Toc188353935)

[Table S 20. Fixed-Effects ANOVA results 47](#_Toc188353936)

[Table S 21. IA condition and BSM. 48](#_Toc188353937)

[Study 4 49](#_Toc188353938)

[*Table S 22.* Correlation Study 4. 49](#_Toc188353939)

[Table S 23. Fixed-Effects ANOVA results 50](#_Toc188353940)

[Table S 24. IA condition and BSM. 52](#_Toc188353941)

[Subgroup Analysis – all following analyses only include state educated participants 55](#_Toc188353942)

[Study 1a 55](#_Toc188353943)

[Table S 25. Main effect condition or Communion, Agency, Toxicity, and Trust*.* 55](#_Toc188353944)

[Table S 26*.* IA Condition and Belief in School Meritocracy for Communion, Agency, Toxicity, and Trust. 56](#_Toc188353945)

[Study 1b 57](#_Toc188353946)

[Table S 27. Communion and Agency (privately educated as reference group) 57](#_Toc188353947)

[Table S 28. Group Entitativity, Permeability and Status (privately educated as reference group). 58](#_Toc188353948)

[Table S 29. Interdependence, Influence and Conflict (privately educated as reference group). 59](#_Toc188353949)

[Table S 30. Communion and Agency by group and BSM (privately educated as reference group). 60](#_Toc188353950)

[Table S 31. Communion and Agency by group and BSM (privately educated as reference group). 62](#_Toc188353951)

[Table S 32. Interdependence, influence, and conflict by group and BSM (privately educated as reference group). 64](#_Toc188353952)

[Study 2 66](#_Toc188353953)

[Table S 33. Fixed-Effects ANOVA results. 66](#_Toc188353954)

[Table S 34. IA Condition and BSM on Trust. 67](#_Toc188353955)

[Table S 35. IA Condition and BSM on Representation. 68](#_Toc188353956)

[Table S 36. IA Condition and BSM on Agency. 69](#_Toc188353957)

[Table S 37. IA Condition and BSM on Communion. 70](#_Toc188353958)

[Study 3 71](#_Toc188353959)

[Table S 38. Fixed-Effects ANOVA results 71](#_Toc188353960)

[Table S 39. IA condition and BSM. 72](#_Toc188353961)

[Study 4 73](#_Toc188353962)

[Table S 40. Fixed-Effects ANOVA results 73](#_Toc188353963)

[Table S 41. IA condition and BSM. 75](#_Toc188353964)

[Participants Education Sector as Moderator 78](#_Toc188353965)

[Study 1a 78](#_Toc188353966)

[Table S 42. IA Condition and education sector on Communion, Agency, Toxicity and Trust study 1a. 78](#_Toc188353967)

[Study 2 79](#_Toc188353968)

[Table S 43. IA Condition and education sector on Trust.. 79](#_Toc188353969)

[Table S 44. IA Condition and Education Sector on Representation. 80](#_Toc188353970)

[Table S 45. IA Condition and Education Sector on Agency. 81](#_Toc188353971)

[Table S 46. IA Condition and Education Sector on Communion. 82](#_Toc188353972)

[Study 3 83](#_Toc188353973)

[Table S 47. IA condition and Education Sector Study 3. 83](#_Toc188353974)

[Study 4 84](#_Toc188353975)

[Table S 48. IA condition and BSM. 84](#_Toc188353976)

# Material

## Study 1a

Introduction Task

On the following pages, you will be presented with several brief descriptions of politicians in the fictional town Fairview. We are interested in what you think of each politician. We know that often people judge politicians based on the party they belong to, but we are just interested in what you think of them as individuals and their ability to participate in politics. For each individual you will therefore see some general and personal information, but no party membership.
Please read each politician's information carefully and indicate your perception.

*There are no right or wrong answers.*

**Vignettes**

*Vignette A:* David Smith is 48 years old and lives in the south of Fairview. He attended Preston Park Comprehensive school (state school) and is married with 2 children. In his free time, he is an active member of the photography association and enjoys spending time with family and friends. He has been active in politics since 2018.

*Vignette B:* Debbie Brown is 46 years old and lives in the northwest of Fairview. She attended Peacehaven Community school (state school) and is married with 3 children. In her free time, she is active for the local football association and makes plenty of time for travelling. She has been active in politics since 2008.

*Vignette C:* Charlie Thomson is 35 years old and lives in the east of Fairview. She attended Havelock Academy (state school) and cohabites with her partner. In her free time, she tries to go for a run a couple of times a week and likes to go out for dinner with friends. She has been active in politics since 2018.

*Vignette D:* Megan Robertson is 38 years old and lives in South Fairview. She attended Kingsbridge Independent School (fee-paying school). She is married and expecting a first child. In her free time, she listens often to music and likes to go to a concert. Besides that, she has been playing the guitar from a young age. She has been active in politics since 2016.

*Vignette E:* Ronald Wilson is 45 years old and lives in the west of Fairview. He attended Eton College (fee-paying school). He cohabites with his partner and has 2 children. In his free time, he likes to go for a walk or a run, and always has a good book to read. He has been active in politics since 2010.

*Vignette F:* Michael Campbell is 51 years old and lives in the east of Fairview. He attended Harrow School (fee-paying school) and is married. In his free time, he plays tennis twice a week and enjoys cooking for his friends. He has been active in politics since 2012.

Items

Based on the short description, how is your perception of [name of politician]? Please indicate your perception on a scale from 1 = Not at all to 5 = Extremely.

[*name of politician*] is …

1. decisive; (2) confident; (3) capable; (4) intelligent; (5) cooperative; (6) out of touch with ordinary people; (7) moral; (8) honest; (9) domineering; (10) corrupt; (11) eloquent; (12) has integrity; (13) reliable; (14) opportunistic; (15) manipulative; (16) friendly; (17) well-connected

Thinking about *[name of politician]* again, please imagine he is part of the council in your city. Please indicate your perception on a scale from *1 = Not at all* to *11 = Completely*.

- I would trust that council.
- I would feel represented by that council.
- I would respect that council.

In politics people sometimes talk of “left” and “right”. Where would you place [name of politician] on this scale, where 0 means the left and 10 means the right?

- Concerning economic issues
- Concerning moral issues

## Study 1b

| Scale | Items |
| --- | --- |
| **Group ratings:**  We are interested in how different groups are considered in British society. We are not interested in your personal beliefs but in how you think they are viewed by others.  (1 = Not at all;  5 = Extremely) | How domineering is this group, as viewed by society?  How manipulative is this group, as viewed by society?  How corrupt is this group, as viewed by society?  How confident is this group, as viewed by society?  How intelligent is this group, as viewed by society?  How eloquent is this group, as viewed by society?  How in touch with the ordinary people is this group, as viewed by society?  How moral is this group, as viewed by society?  How honest is this group, as viewed by society?  How much does this group act with integrity, as viewed by society?  How well-connected is this group, as viewed by society?  How prestigious are the jobs typically achieved by members of this group?  How economically successful have members of this group been?  Resources that go to members of this group are likely to take away from resources of people like me.  The more power members of this group have, the less power people like me are likely to have.  People like me rely on members of this group to help us achieve important goals.  People like me are in cooperative relationships with members of this group to achieve common goals.  Members of this group are good at building relationships with influential people.  Members of this group are good at using their connections and network to make things happen.  People who are a [group name] feel like a group to me.  Is it easy to become a member of [group name]? |
| **Belonging to group**  We all can be part of multiple groups. To which extent do you feel you belong to these groups on a scale from 0 (do not belong at all) to 100 (completely belong)? | People who were privately educated (in fee-paying schools)  People who attended state school  Rich people  Poor people  Lower educated  Higher educated  Politicians  People from the working class  People from the upper class  Doctors  Homeless People  Elderly |

## Study 2

**PART I**

**Council Task Instructions**
On the following page, you will be presented with the government of a fictional city. Specifically, we will show you five people who are the representatives of the larger city council. We are interested in what you think of the council. We know that often people judge candidates on the basis of the political party they belong to, but we are just interested in what you think of them as individuals and their ability to govern and represent the city. For each individual you will therefore see some general and personal information, but no disclosure of party membership.

Please read each member's information carefully. We will ask you some question about them later. The next button will appear after 1 minute.

Member 1: David Smith; Mayor of Fairview; Elected since 2018; Born 1974; Married with 2 children; Active member the Fairview photography association; [D.Smith@fairview.uk](mailto:D.Smith@fairview.uk)

Member 2: Debbie Brown; Representative for Fairview North; Elected since 2008; Born 1976; Married with 3 children; Active member the Fairview football association; [D.Brown@fairview.uk](mailto:D.Brown@fairview.uk)

Member 3: Charlie Thomson; Representative for Fairview East; Elected since 2018; Born 1988; Married; Active member the Fairview chess association; [C.Thomson@fairview.uk](mailto:C.Thomson@fairview.uk)

Member 4: Megan Robertson; Representative for Fairview West; Elected since 2018; Born 1986; Expecting first child; Active member the Fairview art association; [M.Robertson@fairview.uk](mailto:M.Robertson@fairview.uk)

Member 5: Ronald Wilson; Representative for Fairview South; Elected since 2014; Born 1975; Married with 1 child; Active member the Fairview running association; [R.Wilson@fairview.uk](mailto:R.Wilson@fairview.uk)

In the majority state school and majority private school condition the following information about schools were embedded within the portraits:

Fairview Park Comprehensive School (state school/fee-paying school)

Peacehaven Community School (state school/ state school)

Eton College (fee-paying school/ fee-paying school)

Havelock Academy (state school/ fee-paying school)

Kingsbridge Community College (state school/ fee-paying school)

**Manipulation Check**

|  | Yes | No | Unsure/ Don't know |
| --- | --- | --- | --- |
| Name (1) |  |  |  |
| Phone Number (2) |  |  |  |
| Education (3) |  |  |  |
| Marital status (4) |  |  |  |
| Election Year (5) |  |  |  |
| Pets (6) |  |  |  |
| Email address (7) |  |  |  |
| Jurisdiction (8) |  |  |  |
| Political Party (9) |  |  |  |

To check that you have read the presented information carefully, please indicate which of the following information was presented.

**Council Perception Items**

Based on the provided information, we are interested in your perception of the council. We are interested in what you think about the council as a whole, not any specific member.

Please indicate your agreement with each statement on a scale from 1 (Strongly disagree) to 7 (Strongly agree).

The council is … (1) trustworthy, (2) competent, (3) intelligent, (4) in touch with the ordinary people, (5) responsible, (6) warm, (7) honest, (8) kind, (9) sociable, (10) down to earth.

Thinking of the council, please indicate the extent to which you agree with the following statements on a scale from 1 (Strongly disagree) to 7 (Strongly agree).

The council represents my view.

The council has representatives that are similar to me in terms of my personal characteristics and background.

The council gives me a sense of belonging and pride in my identity.

The council will use the authority given to them in a responsible manner for the citizens.

**PART II**

**Internship Task Instructions**

The council offers internship opportunities for GCSE students. The aim is to engage young people and foster political interest.
In following you will see four candidates’ profiles. To ensure equal opportunities, all CVs have been formatted to the same layout and there is no indication of gender or ethnicity.
Please read each candidate’s profile and rate their internship compatibility.
There are no right or wrong answers.

**Internship Vignettes**

**M. Ford**
**Education**
**Since 2017 Fairview Park Academy (independent school)**
Currently studying for GCSEs in the following subjects:
German
Chemistry
History
English Language
Geography
Physical Education
 
**Work Experiences & Interests**
Twice a week I walk the dogs of my neighbor and I help with the training of the younger local volleyball team. I am active in the local volleyball and swim team. I practice regularly and participate in competitions.

**B. Smith
Education**
**Since 2017 Kingsbridge Community College**
Currently studying for GCSEs in the following subjects:
Math
Media Studies
Geology
English History
Spanish
Computer Science

**Work Experiences & Interests**
I tutor two children aged 11 and 13 in Math and Spanish twice a week. I am interested in art and nature photography. I regularly spend my free time in nature trying to get a good shot.

**S. Jones
Education
Since 2017 Turnbridge Wells (independent school)**
**Currently studying for GCSEs in the following subjects:**
 Math
 Spanish
 History
 English Language
 English History
 Music

 **Work Experiences & Interests**
 I regularly look after my Aunt’s children aged 5 and 3 and I have to ensure the children are safe, are put to bed at the correct time and sometimes I also have to prepare their dinner. I am a keen musician. I have loved to sing and play the guitar since I was a kid. I am also a member of the school choir.

**M. Berry
Education
Since 2017 Peacehaven Community College**
Currently studying for GCSEs in the following subjects:
Geography
Math
Art
English Language
German
Physical Education

**Work Experiences & Interests**
I regularly help with the training of children aged 7 to 9 in the local soccer club. I also play for the team as a central midfielder and practice twice a week.

**Internship Task Items**

Please indicate your perception of the candidate on a scale from 1 (very negative/ strongly disagree) to 7 (very positive/ strongly agree).

My overall perception of this candidate is …

I think this candidate will succeed in the internship.

I think this candidate fits well with the council.

I think the council will offer an internship to this candidate.

I think this candidate is well suited to work in politics.

How much do you think the candidate should be paid in GBP per hour? (scale between 5 and 100)

## Study 3

**Instruction Bimboola**

In this study you will learn about a new society called Bimboola. Bimboola is just like any other society. You will learn some information about Bimboola and how the society functions.
Please read the information carefully. We will ask you about your perception of Bimboola.
In the next slides you will be informed about the educational system of Bimboola.

**Educational System**

School attendance is compulsory. Compulsory education starts when children are 6 years old and ends when they are aged 16. Parents can choose which of two types of school they want their child to attend.

**Option 1: Private-funded schools:** The second type are private, fee-paying schools. That means parents have to pay tuition-fees if they want their child to attend these schools.

**Option 2: State-funded schools:** The first type of school are state-funded schools. These schools are financed by the Bimboola Government through taxes on citizens and they are free to attend for all Bimboola citizens.

In Bimboola society, around 10% of children attend a private, fee-paying school, and around 90% attend a state-funded school.

**Political System**
Bimboola is a representative democracy. Every four years, the Bimboola citizens vote for their parliament in free and fair elections. Citizens can cast one vote for a political party. After the election, the share of seats in the parliament represent the proportion of the votes for each party. That means that, if a political party receives 10% of the votes, they receive 10% of the seats. The party with the most votes provides the president and runs the government.
The parliament consists of 100 members. The parliament is in charge of running the country and pass legislation (e.g., passing tax laws, immigration laws).

Condition Underrepresentation: **0 out of 100 current parliament members attended a private school.**
Condition Overrepresentation: **35 out of 100 current parliament members attended a private school.**
Condition Matched Representation: **10 out of 100 current parliament members attended a private school.**

| Scale | Items |
| --- | --- |
| Representation  (1 = Not at all;  9 = Very much) | To what extent do you think the parliament is representative of Bimboola's society?  To what extent do you think Bimboola's parliament represents the will of the people?  To what extent do you think politicians in Bimboola's parliament are in touch with the ordinary people? |
| Trust  (1 = Not at all;  9 = Very much) | To what extent do you think Bimboola's parliament is trustworthy?  To what extent can you generally trust the people who run Bimboola's government to do what is right?  To what extend can most public officials in Bimboola be trusted to do what is right without having to constantly cheek on them?  To what extent do you think politicians in Bimboola's parliament are moral? |
| Deservingness  (1 = Not at all;  9 = Very much) | To what extent do you think politicians in Bimboola's parliament deserve their position? |
| Merit  (1 = Not at all;  9 = Very much) | To what extent do you think politicians in Bimboola's parliament got their position due to their effort?  To what extent do you think politicians in Bimboola's parliament got their position due to their talent?  To what extent do you think politicians in Bimboola's parliament got their position due to their family background?  To what extent do you think politicians in Bimboola's parliament got their position due to luck? |
| Merit School System  (1 = Not at all;  9 = Very much) | In Bimboola, to what extent does the type of school that children attend depend on their talent?  In Bimboola, to what extent does the type of school that children attend depend on their effort?  In Bimboola, to what extent does the type of school that children attend depend on luck?  In Bimboola, to what extent does the type of school that children attend depend on their family background? |
| Democratic Values  (1 = Strongly Disagree;  7 = Strongly Agree) | A democratic system is the best political system that we have.  I think of myself as someone who values democracy.  I think of myself as someone who supports democratic governance.  Democratic values are important to me. |
| Representation UK Government  (1 = Strongly Disagree;  7 = Strongly Agree) | The government represent my view.  The government has representatives that are similar to me in terms of my personal characteristics and background.  The government gives me a sense of belonging and pride in my identity.  The government will use the authority given to them in a responsible manner for the citizens. |
| Proportion of private educated  (0% to 100%) | Please indicate in percent the proportion of private educated individuals in ....  … the general population in the UK.  … serving as politicians. |

## Study 4

**Task Introduction**

On the following pages, you will be presented with a brief description of a politician. We are interested in what you think of the politician. We know that often people judge politicians based on the party they belong to, but we are just interested in what you think of them as individuals and their ability to participate in politics. You will therefore see some general and personal information, but no party membership.
Please read the politician's information carefully and indicate your perception.
There are no right or wrong answers.

**Conditions**

***State school condition:*** David Smith grew up in Fairview with his parents and one older sister. His father worked as an accountant and his mother as a teacher. He attended Preston Park Comprehensive School *(state school)*. After school he attended a high-ranked university. He is married with 2 children. In his free time, he tries to go for a run a couple of times a week and enjoys spending time with family and friends. He has been active in politics since 2010.

***Merit condition:*** David Smith grew up in Fairview with his parents and one older sister. His father worked as an accountant and his mother as a teacher. He attended Preston Park Independent School *(fee-paying school)*. All throughout school, he worked hard and won a competitive scholarship to a high-ranking university. He is married with 2 children. In his free time, he tries to go for a run a couple of times a week and enjoys spending time with family and friends. He has been active in politics since 2010.

***Private school condition:*** David Smith grew up in Fairview with his parents and one older sister. His father worked as an accountant and his mother as a teacher. He attended Preston Park Independent School *(fee-paying school)*. After school he attended a high-ranked university. He is married with 2 children. In his free time, he tries to go for a run a couple of times a week and enjoys spending time with family and friends. He has been active in politics since 2010.

**Items**

***Merit:*** Based on the short description, what is your perception of Davod Smith? Please indicate your answer on a scale from 1 (not at all) to 5 (extremely).

David Smith earnt his position in politics.

David Smith worked hard to get into politics.

David Smith got into politics through is talents.

David Smith had an unfair advantage compared to others trying to get into politics.

David Smith did not have to work very hard to get into politics.

David Smith did not have much talent to get into politics.

David Smith deserves his political position.

David Smith obtained his position through sheer luck.

David Smith got into politics through family connections.

***Agency and Communion:*** Based on the short description, what is your perception of Davod Smith? Please indicate your answer on a scale from 1 (not at all) to 5 (extremely).

David Smith is out of touch with the ordinary people.

David Smith is capable.

David Smith is moral.

David Smith is honest.

David Smith is well-connected.

David Smith has integrity.

David Smith is corrupt.

David Smith is opportunistic.

***Trust:*** Thinking about David Smith again, please imagine he is your local councilor. Please indicate your answer on a scale from 0 (not at all) to 10 (completely).

To what extent do you trust your local councilor - David Smith?

To what extent do you think your local councilor, David Smith, cares about the citizens and his constituency?

To what extent do you think your local councilor, David Smith, is reliable?

***Representation:*** Please indicate your answer on a scale from 1 (not at all) to 5 (completely).

- To what extent do you think David Smith's views are representative of you personally?
- To what extent do you think David Smith's views are representative of English society?
- To what extent do you think David Smith's views are representative of English politicians?

***Political Orientation:*** In politics people sometimes talk of “left” and “right”. Where would you place David Smith on this scale, where 0 means the left and 10 means the right?

Concerning economic issues

Concerning moral issues

## Individual Differences Measures in all Studies

| Feeling Thermometer  (measured on a scale from 0 to 100) | Ratings between 50 degrees and 100 degrees mean that you feel favorable and warm toward the group. Ratings between 0 degrees and 50 degrees mean that you don't feel favorable toward the group and that you don't care too much for that group. You would rate the group at the 50 degree mark if you don't feel particularly warm or cold toward the group.  People who were privately educated (in fee-paying schools)  People who attended state school  Rich people  Poor people  Lower educated  Higher educated  Politicians |
| --- | --- |
| Political Orientation  (0 = Left, 10 = Right) | In politics people sometimes talk of “left” and “right”. Where would you place yourself on this scale, where 0 means the left and 10 means the right?  Concerning economic issues  Concerning moral issues |
| Interest in Politics  (1 = Not at all interested;  2 = Not very interested; 3 = Somewhat interested;  4 = Very interested) | How interested would you say you are in politics? |
| Populism  (1 = Strongly disagree to 5 = Strongly agree) | The politicians in Parliament need to follow the will of the people.  The people, and not the politicians, should make the most important political decisions.  The political differences between the elite and the people are larger than the differences among the people.  I would rather be represented by an ordinary citizen than by a professional politician.  What people call ‘compromise’ in politics is really just selling out on one’s principles.  The established politicians who claim to defend our interest, have often betrayed the people.  Elected officials talk too much and take too little action. |

| Institutional Trust  (0 = No trust at all;  20 = Complete trust) | Please indicate on a scale of 0-10 how much you personally trust each of the institutions. 0 means you do not trust an institution at all, and 10 means you have complete trust.  UK’s Parliament  UK’s Politicians  The legal system |
| --- | --- |
| Representation UK Government  (1 = Strongly disagree to 7 = Strongly agree)  Only measured in Study III | Thinking of the UK government, please indicate the extent to which you agree with the following statements.  The government represent my view.  The government has representatives that are similar to me in terms of my personal characteristics and background.  The government gives me a sense of belonging and pride in my identity.  The government will use the authority given to them in a responsible manner for the citizens. |
| Political Efficacy  (1 = not at all confident/ able;  5 = completely confident/ able) | How confident are you in your own ability to participate in politics?  How able do you think you are to take an active role in a group involved in politics? |
| Political Cynicism  (1 = Strongly Disagree;  7 = Strongly Agree) | Politicians are out of touch with life in the real world.  Politicians are interested only in people’s votes, not in their opinions.  It seems like politicians only care about themselves or special interests.  I’m satisfied with the way that government works in our country. |
| Relative Deprivation  (1 = Strongly Disagree;  5 = Strongly Agree)  Only measured in Study III | Whichever way you look at it, people like me always get short-changed.  If we need something from the government, people like us always have to wait longer.  When an economic crisis strikes, people like me are always the first to be laid off. |
| Economic System Justification  (1 = Strongly Disagree;  5 = Strongly Agree) | Economic positions are legitimate reflections of people’s achievements.  If people work hard, they almost always get what they want.  Most people who don’t get ahead in our society should not blame the system; they have only themselves to blame. |
| Perceived Income differences  (1 = Not at all;  5 = Completely) | To what extent do you think that the distribution of the resources in the UK is equal?  To what extent do you think that the distribution of the resources in the UK is unequal? |
| Subjective Inequality in Everyday Life  (1 = Strongly Disagree;  5 = Strongly Agree) | Among the people I surround myself with, some can afford to buy a lot more and better things than others.  Among the people I know, some cannot afford unforeseen expenses and others cope with them without any difficulty  I know people who can afford to save money and others who struggle to get by.  Among the people I know, some live in bigger and more luxurious homes than others. |
| Belief in School Meritocracy  (1 = Not at all;  5 = Very much) | Everyone has the same chances to succeed at school.  At school, children obtain the grades they deserve.  At school, students who obtain good grades are those who have worked hard.  For children at school, where there is a will, there is a way |
| Support for abolishing private schools  (1 = Strongly Disagree;  7 = Strongly Agree) | I am in favour of abolishing private schools in the UK.  I believe that the tax advantages currently enjoyed by private schools should be removed.  I believe that parents should have the right to send their children to private schools if they want to do so.  I am against abolishing private schools in the UK. |
| Identity  (1 = Strongly disagree;  5 Strongly agree) | I identify with …  The United Kingdom  People with a similar level of education to me own  People with a similar level of income to my own. |
| Political Participation  Only measured in Study 2 | There are different ways of trying to improve things in the United Kingdom or help prevent things from going wrong. During the last 12 months, have you done any of the following?  Contacted a politician, government, or local government official?  Worked in a political party or action group?  Signed a petition?  Taken part in a lawful public demonstration?  Posted or shared anything about politics online, for example on blogs, via email or on social media such as Facebook or Twitter? |
| Party Preference  Only measured in Study 2 | Which political party do you feel closest to?  I do not feel close to any political party  Labour Party  Scottish National Party  Liberal Democrats  Conservative Party  Sinn Féin  Plaid Cymru  Social Democratic and Labour Party  Alba Party  Green Party of England and Wales  Alliance Party of Northern Ireland  Ulster Unionist Party  Scottish Greens  Green Party Northern Ireland  Traditional Unionist Voice  People Before Profit  Democratic Unionist Party |
| Party Identification  (1 = Strongly disagree;  5 Strongly agree)  Only measured in Study 2 | I identify with [party chosen in party preference question]. |
| Vote  Only measured in Study 2 | Suppose there will be a general election this week. For which party would you vote in this election?  Labour Party  Scottish National Party  Liberal Democrats  Conservative Party  Sinn Féin  Plaid Cymru  Social Democratic and Labour Party  Alba Party  Green Party of England and Wales  Alliance Party of Northern Ireland  Ulster Unionist Party  Scottish Greens  Green Party Northern Ireland  Traditional Unionist Voice  People Before Profit  Democratic Unionist Party  I would not vote or cast a blank vote  I am not eligible to vote |
| In what year were you born? | In what year were you born? |
| Gender | Are you …  Woman  Man  Non binary  Transgender  Prefer not to say  Other, please specify |
| Occupation | Which best describes your situation of the last seven days?  In paid work  In education  Unemployment  Permanently sick or disabled  Retired  In community or military service  Doing housework, looking after children or other persons |
| Education | What is the highest level of education you have successfully completed?  No formal qualifications  GCSE/O-levels/CSE, or NVQ/SVQ Level 1 or 2, or City and Guilds Level 1 or 2/Craft/Intermediate, or GNVQ/GSVQ Foundation or Intermediate level, or equivalent  A-levels, or NVQ/SVQ Level 3, or City and Guilds Level 3/Advanced/Final, or GNVQ/GSVQ Advanced Level, or equivalent  NVQ/SVQ Level 4 or 5, or City and Guilds Level 4/Full Technological, or equivalent  Higher National Certificate, Higher National Diploma, Foundation Degree  Undergraduate degree (BA/BSC/other)  Graduate degree (MA/MSc/MPhil/other)  Post-graduate diploma or certificate (e.g., PGCE)  Doctoral degree (PhD)  Other (please specify) |
| Income  (1 = very easy;  5 = very difficult) | To what extent do you feel that you are able to live a comfortable life with your current household income? |
| Urbanization | Would you consider the area in which you live to be …?  The open countryside  A village/small town  A medium to large town  Suburbs or outskirts of big city  Big city |
| Ethnicity | Which ethnic group do you consider yourself to belong to?  English/Welsh/Scottish/Northern Irish/British  Irish  Gypsy or Irish Traveller  Any other White background, please describe  White and Black Caribbean  White and Black African  White and Asian  Any other Mixed/Multiple ethnic background, please describe  Indian  Pakistani  Bangladeshi  Chinese  Any other Asian background, please describe  African  Caribbean  Any other Black/African/Caribbean background, please describe  Arab  Any other ethnic group, please describe |
| Type of School | Did you attend state school or public school (fee-paying school)?  Public school (fee-paying school)  State school |
| Children | Do you have children?  Yes  No |
| Children School | Did one or more of your children attend public school (fee-paying school)?  Yes  No  Both (i.e., attend public school and state school) |
| Comment | Anything you want to comment on? |

# Additional Analysis

## Study 1a

Table S 1. Correlation Study 1a.

|  | BSM | Political Orientation | Level of Education | Own type of education | Children’s education |
| --- | --- | --- | --- | --- | --- |
| BSM | - |  |  |  |  |
| Political Orientation | .525*** | - |  |  |  |
| Level of Education | -.293** | -.185** | - |  |  |
| Own type of education | -.012 | -.132+ | -.108 | - |  |
| Children’s education | -.146 | -.199* | -.037 | .309*** | - |

Note. + indicates p < .10. * indicates p < .05, ** indicates p < .01. *** indicates p < .001.

### Table S 2. *Main effect condition or Communion, Agency, Toxicity, and Trust.*

|  | **Communion** | | | **Agency** | | | **Toxicity** | | | **Trust** | | |
| --- | --- | --- | --- | --- | --- | --- | --- | --- | --- | --- | --- | --- |
| *Predictors* | *Estimates* | *CI* | *p* | *Estimates* | *CI* | *p* | *Estimates* | *CI* | *p* | *Estimates* | *CI* | *p* |
| (Intercept) | 0.35 | 0.32 – 0.38 | **<0.001** | 0.33 | 0.29 – 0.36 | **<0.001** | 0.20 | 0.16 – 0.25 | **<0.001** | 0.48 | 0.43 – 0.54 | **<0.001** |
| Privately educated | -0.06 | -0.07 – -0.04 | **<0.001** | 0.06 | 0.05 – 0.07 | **<0.001** | 0.09 | 0.08 – 0.11 | **<0.001** | -0.09 | -0.11 – -0.07 | **<0.001** |
| BSM (centred) | -0.00 | -0.00 – 0.00 | 0.393 | 0.00 | -0.00 – 0.00 | 0.925 | -0.00 | -0.00 – 0.00 | 0.090 | -0.00 | -0.00 – 0.00 | 0.250 |
| Age (centred) | -0.01 | -0.04 – 0.01 | 0.317 | -0.02 | -0.04 – 0.01 | 0.220 | 0.02 | -0.01 – 0.05 | 0.201 | -0.04 | -0.08 – -0.00 | **0.035** |
| Male | 0.01 | 0.01 – 0.02 | **<0.001** | 0.00 | -0.00 – 0.01 | 0.137 | -0.01 | -0.01 – 0.00 | 0.053 | 0.01 | 0.01 – 0.02 | **0.001** |
| LR (centred) | 0.06 | 0.02 – 0.09 | **0.001** | 0.05 | 0.02 – 0.09 | **0.003** | -0.03 | -0.07 – 0.01 | 0.193 | 0.04 | -0.01 – 0.10 | 0.121 |
| Own Education Private | 0.01 | -0.01 – 0.04 | 0.358 | 0.02 | -0.00 – 0.05 | 0.067 | -0.02 | -0.04 – 0.01 | 0.223 | 0.01 | -0.02 – 0.03 | 0.708 |
| **Random Effects** | | | | | | | | | | | | |
| σ^2^ | 0.01 | | | 0.01 | | | 0.01 | | | 0.02 | | |
| τ_00_ | 0.00 _id_ | | | 0.01 _id_ | | | 0.01 _id_ | | | 0.02 _id_ | | |
| ICC | 0.25 | | | 0.37 | | | 0.48 | | | 0.43 | | |
| N | 202 _id_ | | | 202 _id_ | | | 202 _id_ | | | 202 _id_ | | |
| Observations | 1211 | | | 1212 | | | 1212 | | | 1212 | | |
| Marginal R^2^ / Conditional R^2^ | 0.082 / 0.311 | | | 0.069 / 0.412 | | | 0.116 / 0.543 | | | 0.090 / 0.484 | | |

### Table S 3*. IA Condition and Belief in School Meritocracy for Communion, Agency, Toxicity, and Trust.*

|  | **Communion** | | | **Agency** | | | **Toxicity** | | | **Trust** | | |
| --- | --- | --- | --- | --- | --- | --- | --- | --- | --- | --- | --- | --- |
| *Predictors* | *Estimates* | *CI* | *p* | *Estimates* | *CI* | *p* | *Estimates* | *CI* | *p* | *Estimates* | *CI* | *p* |
| (Intercept) | 0.36 | 0.32 – 0.39 | **<0.001** | 0.33 | 0.30 – 0.37 | **<0.001** | 0.20 | 0.16 – 0.25 | **<0.001** | 0.49 | 0.44 – 0.54 | **<0.001** |
| Privately educated | -0.06 | -0.07 – -0.04 | **<0.001** | 0.06 | 0.05 – 0.07 | **<0.001** | 0.09 | 0.08 – 0.11 | **<0.001** | -0.09 | -0.11 – -0.07 | **<0.001** |
| BSM (centred) | 0.02 | -0.00 – 0.03 | 0.063 | 0.02 | 0.00 – 0.04 | **0.024** | 0.01 | -0.01 – 0.03 | 0.535 | 0.02 | -0.00 – 0.05 | 0.081 |
| IA | 0.02 | 0.01 – 0.04 | **0.003** | 0.00 | -0.01 – 0.02 | 0.563 | -0.02 | -0.03 – -0.01 | **0.003** | 0.02 | 0.01 – 0.04 | **0.006** |
| Age (centred) | -0.00 | -0.00 – 0.00 | 0.293 | -0.00 | -0.00 – 0.00 | 0.973 | -0.00 | -0.00 – 0.00 | 0.094 | -0.00 | -0.00 – 0.00 | 0.191 |
| Male | -0.02 | -0.04 – 0.01 | 0.183 | -0.02 | -0.05 – 0.01 | 0.142 | 0.02 | -0.01 – 0.05 | 0.194 | -0.05 | -0.09 – -0.01 | **0.017** |
| LR (centred) | 0.01 | -0.00 – 0.01 | 0.062 | -0.00 | -0.01 – 0.01 | 0.980 | -0.01 | -0.01 – 0.00 | 0.132 | 0.01 | -0.00 – 0.02 | 0.104 |
| Own Education Private | 0.05 | 0.02 – 0.09 | **0.002** | 0.05 | 0.01 – 0.09 | **0.007** | -0.03 | -0.07 – 0.02 | 0.208 | 0.04 | -0.02 – 0.09 | 0.203 |
| σ^2^ | 0.01 | | | 0.01 | | | 0.01 | | | 0.02 | | |
| τ_00_ | 0.00 _id_ | | | 0.01 _id_ | | | 0.01 _id_ | | | 0.02 _id_ | | |
| ICC | 0.24 | | | 0.36 | | | 0.49 | | | 0.42 | | |
| N | 202 _id_ | | | 202 _id_ | | | 202 _id_ | | | 202 _id_ | | |
| Observations | 1211 | | | 1212 | | | 1212 | | | 1212 | | |
| Marginal R^2^ / Conditional R^2^ | 0.107 / 0.317 | | | 0.084 / 0.413 | | | 0.120 / 0.548 | | | 0.111 / 0.489 | | |

*.*

## Study 1b

Study 1b explores how different social groups – including the privately educated, the state educated, and politicians – are perceived by U.K. society with respect to key dimensions of social perception (agency and communion), trust, and representation. We expect that the *privately educated* group will be perceived as more agentic and less communal than the *state-educated* group.

**Method**

***Participants and Experimental Design***

The final sample consisted of 196 U.K. participants (103 women, 89 men, 4 other; M_Age_ = 44.27 SD_Age_ = 14.24) who were recruited via Prolific Academics. After giving informed consent, each participant rated how 12 social groups, presented in a randomized order, are viewed by society. Participants rated the communion, agency, social structural dimensions, group entitativity, group influence, and group permeability of each group. After rating all groups, participants filled out multiple questionnaires about their social and political attitudes, provided socio-demographic information, were fully debriefed and compensated.

***Measures***

**Communion and Agency.** Participants indicated how society perceives 12 social groups: (1) people who attend private school (i.e., fee-paying school), (2) people who are rich, (3) people who are highly educated, (4) politicians, (5) people who are poor, (6) people who are lower educated, (7) people who attend state school, (8) people from the working class, (9) people from the upper class, (10) elderly people, (11) homeless people, and (12) doctors. Each group was rated on 11 items on a scale from 1 (Not at all) to 5 (Extremely): *(1) domineering, (2) manipulative, (3) corrupt, (4) confident, (5) intelligent, (6) eloquent, (7) in touch with the ordinary people, (8) moral, (9) honest, (10) integrity*, and *(11) well-connected*. The items were inspired by Abele et al. (2016) and Cuddy et al. (2008), as well as research on political leadership skills (for an overview see Aaldering & Vliegenthart, 2016).

**Social Structural Correlates.** Social structural relations of each group were measured with 10 items by Cuddy et al. (2008) on a scale from 1 (Not at all) to 5 (Extremely). An example item reads: *‘How prestigious are the jobs typically achieved by members of this group?’*

**Group Influence, Entitativity & Permeability.** Perceived influence was measured with 2 items, i.e., "Members of this group are good at building relationships with influential people." and "Members of this group are good at using their connections and network to make things happen." Perceived group entitativity was measured with 1 item, i.e., "[Group name] feel like a group to me." Perceived group permeability was measured with 1 item, i.e., "Is it easy to become [Group name]." Each item was measured on a scale from 1 (Not at all) to 5 (Extremely).

**Individual differences.** Belief in school meritocracy (Cronbach’s alpha = .86), political orientation (r = .77), institutional trust (Cronbach’s alpha = .88) and demographics were measured with the same items as in Study 1a.

**Results**

We analysed the data using cluster analysis and multilevel regressions with random intercepts to account for the nested structure of the data (i.e., groups nested within participants). The results show that the privately educated and the state-educated are perceived differently. The *privately educated* group clustered with other privileged and high-status groups (*politicians*, *rich*, and *upper* *class*), while the *state-educated* group clustered with lower status groups (*working* *class* and *elderly*) (Figure S1).


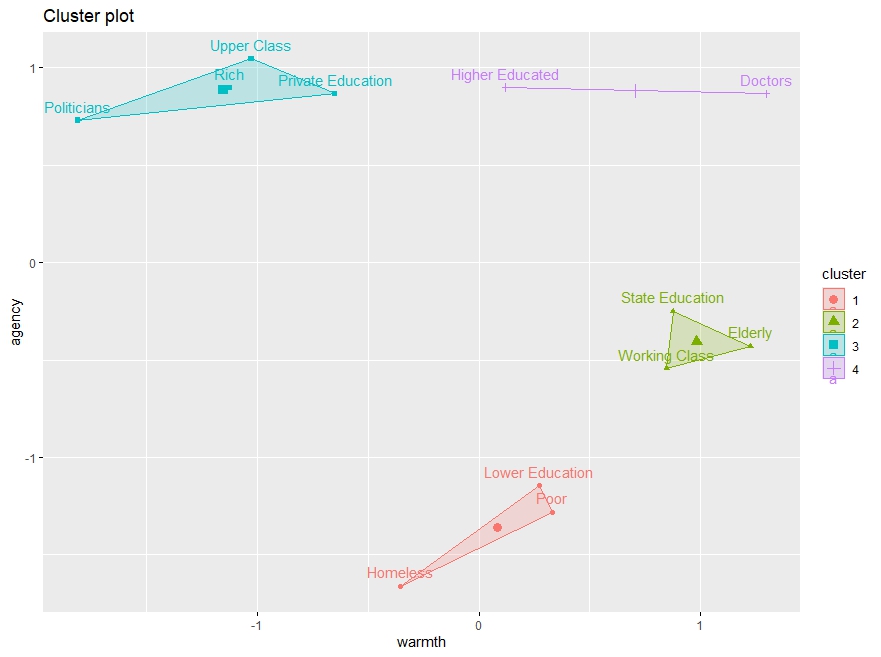


Figure S1. Cluster analysis.

The multilevel regression results further strengthen these findings by showing that the *state-educated* group is perceived as higher on communal traits, while the *privately educated* group is perceived as higher on agentic traits. The *privately educated* group is also seen as a cohesive, high-status group that is difficult to join. Indeed, only becoming a doctor or member of the upper class is perceived as more difficult. Considering the social structural dimensions, the results indicate that the *privately educated* group – unlike the *state-educated* group – is independent from other groups, is highly influential, and has interests that conflict with the interests of other groups (Table S3-S5).

We also investigated whether belief in school meritocracy (BSM) moderated these perceptions. Controlling for participants’ age, gender, political orientation, and school type (as we do in the equivalent analyses below), individuals with higher (compared to lower) BSM perceived the privately educated and state-educated groups as more similar (i.e., the difference in ratings is smaller) on agency, communion, status, influence, and conflict (see Figure S2, Table S6-S8).

### Table S 4. *Correlation Study 1b.*

|  | BSM | Political Orientation | Level of Education | Own type of education | Children’s education |
| --- | --- | --- | --- | --- | --- |
| BSM | - |  |  |  |  |
| Political Orientation | .279*** | - |  |  |  |
| Level of Education | -.193** | -.037 | - |  |  |
| Own type of education | -.056 | -.122+ | -.057 | - |  |
| Children’s education | -.158 | -.140 | -.142 | .001 | - |

Note. + indicates p < .10. * indicates p < .05, ** indicates p < .01. *** indicates p < .001.

Figure S2. DV’s by BSM and Social Group (Private Educated vs. State Educated) in Study 1b.

Table S 5. Communion and Agency (privately educated as reference group)

|  | **Communion** | | | **Agency** | | |
| --- | --- | --- | --- | --- | --- | --- |
| *Predictors* | *Estimates* | *CI* | *p* | *Estimates* | *CI* | *p* |
| (Intercept) | 0.34 | 0.29 – 0.39 | **<0.001** | 0.73 | 0.68 – 0.77 | **<0.001** |
| State Educated | 0.34 | 0.30 – 0.39 | **<0.001** | -0.32 | -0.37 – -0.28 | **<0.001** |
| Upper Class | -0.07 | -0.12 – -0.03 | **0.001** | 0.08 | 0.03 – 0.12 | **<0.001** |
| Working Class | 0.33 | 0.28 – 0.37 | **<0.001** | -0.35 | -0.39 – -0.30 | **<0.001** |
| Rich | -0.08 | -0.12 – -0.03 | **<0.001** | 0.09 | 0.05 – 0.13 | **<0.001** |
| Poor | 0.34 | 0.30 – 0.38 | **<0.001** | -0.50 | -0.54 – -0.46 | **<0.001** |
| Higher Educated | 0.11 | 0.07 – 0.16 | **<0.001** | -0.09 | -0.13 – -0.04 | **<0.001** |
| Lower Educated | 0.34 | 0.30 – 0.38 | **<0.001** | -0.44 | -0.48 – -0.39 | **<0.001** |
| Politicians | -0.20 | -0.25 – -0.16 | **<0.001** | 0.12 | 0.08 – 0.17 | **<0.001** |
| Doctors | 0.37 | 0.33 – 0.42 | **<0.001** | -0.22 | -0.26 – -0.17 | **<0.001** |
| Elderly | 0.42 | 0.38 – 0.47 | **<0.001** | -0.42 | -0.46 – -0.37 | **<0.001** |
| Homeless | 0.30 | 0.26 – 0.34 | **<0.001** | -0.60 | -0.64 – -0.55 | **<0.001** |
| Age centered | -0.00 | -0.00 – -0.00 | **0.013** | 0.00 | -0.00 – 0.00 | 0.093 |
| Male | -0.01 | -0.04 – 0.02 | 0.490 | 0.00 | -0.02 – 0.03 | 0.906 |
| Political Orientation centered | 0.00 | -0.00 – 0.01 | 0.782 | -0.00 | -0.01 – 0.00 | 0.147 |
| Participant state educated | -0.01 | -0.05 – 0.03 | 0.725 | -0.02 | -0.06 – 0.02 | 0.293 |
| **Random Effects** | | | | | | |
| σ^2^ | 0.05 | | | 0.05 | | |
| τ_00_ | 0.01 _id_ | | | 0.00 _id_ | | |
| ICC | 0.11 | | | 0.07 | | |
| N | 191 _id_ | | | 191 _id_ | | |
| Observations | 2292 | | | 2292 | | |
| Marginal R^2^ / Conditional R^2^ | 0.453 / 0.511 | | | 0.538 / 0.573 | | |

Table S 6. Group Entitativity, Permeability and Status (privately educated as reference group).

|  | **Entitativity** | | | **Permeability** | | | **Status** | | |
| --- | --- | --- | --- | --- | --- | --- | --- | --- | --- |
| *Predictors* | *Estimates* | *CI* | *p* | *Estimates* | *CI* | *p* | *Estimates* | *CI* | *p* |
| (Intercept) | 0.65 | 0.57 – 0.73 | **<0.001** | 0.26 | 0.20 – 0.31 | **<0.001** | 0.84 | 0.80 – 0.87 | **<0.001** |
| State Educated | -0.22 | -0.27 – -0.18 | **<0.001** | 0.61 | 0.57 – 0.66 | **<0.001** | -0.31 | -0.34 – -0.28 | **<0.001** |
| Upper Class | 0.04 | -0.01 – 0.08 | 0.111 | -0.08 | -0.12 – -0.03 | **0.001** | 0.05 | 0.02 – 0.08 | **0.001** |
| Working Class | -0.06 | -0.11 – -0.02 | **0.008** | 0.48 | 0.44 – 0.53 | **<0.001** | -0.46 | -0.49 – -0.42 | **<0.001** |
| Rich | -0.01 | -0.05 – 0.04 | 0.783 | -0.03 | -0.08 – 0.01 | 0.172 | 0.06 | 0.03 – 0.09 | **<0.001** |
| Poor | -0.25 | -0.29 – -0.20 | **<0.001** | 0.54 | 0.50 – 0.59 | **<0.001** | -0.71 | -0.74 – -0.68 | **<0.001** |
| Higher Educated | -0.09 | -0.13 – -0.04 | **<0.001** | 0.14 | 0.09 – 0.18 | **<0.001** | -0.02 | -0.05 – 0.01 | 0.253 |
| Lower Educated | -0.28 | -0.33 – -0.24 | **<0.001** | 0.42 | 0.38 – 0.47 | **<0.001** | -0.61 | -0.65 – -0.58 | **<0.001** |
| Politicians | 0.09 | 0.04 – 0.14 | **<0.001** | 0.07 | 0.02 – 0.11 | **0.004** | 0.00 | -0.03 – 0.03 | 0.903 |
| Doctors | 0.04 | -0.01 – 0.08 | 0.111 | -0.10 | -0.15 – -0.06 | **<0.001** | 0.01 | -0.02 – 0.04 | 0.683 |
| Elderly | -0.05 | -0.09 – 0.00 | 0.054 | 0.46 | 0.41 – 0.50 | **<0.001** | -0.39 | -0.42 – -0.36 | **<0.001** |
| Homeless | -0.16 | -0.20 – -0.11 | **<0.001** | 0.42 | 0.37 – 0.46 | **<0.001** | -0.76 | -0.79 – -0.73 | **<0.001** |
| Age centered | 0.00 | 0.00 – 0.00 | **0.006** | 0.00 | -0.00 – 0.00 | 0.842 | 0.00 | 0.00 – 0.00 | **0.002** |
| Male | -0.01 | -0.06 – 0.03 | 0.574 | -0.03 | -0.06 – 0.00 | 0.051 | -0.03 | -0.05 – -0.01 | **0.002** |
| Political Orientation centered | 0.01 | -0.00 – 0.01 | 0.222 | -0.00 | -0.01 – 0.00 | 0.152 | -0.00 | -0.01 – 0.00 | 0.097 |
| Participant state educated | 0.08 | 0.01 – 0.15 | **0.019** | -0.01 | -0.06 – 0.03 | 0.515 | -0.01 | -0.04 – 0.01 | 0.356 |
| **Random Effects** | | | | | | | | | |
| σ^2^ | 0.05 | | | 0.05 | | | 0.02 | | |
| τ_00_ | 0.02 _id_ | | | 0.01 _id_ | | | 0.00 _id_ | | |
| ICC | 0.29 | | | 0.10 | | | 0.07 | | |
| N | 191 _id_ | | | 191 _id_ | | | 191 _id_ | | |
| Observations | 2292 | | | 2292 | | | 2292 | | |
| Marginal R^2^ / Conditional R^2^ | 0.175 / 0.411 | | | 0.541 / 0.587 | | | 0.778 / 0.792 | | |

Table S 7. Interdependence, Influence and Conflict (privately educated as reference group).

|  | **Interdependence** | | | **Influence** | | | **Conflict** | | |
| --- | --- | --- | --- | --- | --- | --- | --- | --- | --- |
| *Predictors* | *Estimates* | *CI* | *p* | *Estimates* | *CI* | *p* | *Estimates* | *CI* | *p* |
| (Intercept) | 0.41 | 0.35 – 0.46 | **<0.001** | 0.86 | 0.82 – 0.90 | **<0.001** | 0.64 | 0.59 – 0.70 | **<0.001** |
| State Educated | 0.20 | 0.16 – 0.24 | **<0.001** | -0.38 | -0.42 – -0.35 | **<0.001** | -0.38 | -0.42 – -0.34 | **<0.001** |
| Upper Class | -0.04 | -0.08 – 0.00 | 0.062 | 0.06 | 0.03 – 0.10 | **0.001** | 0.07 | 0.03 – 0.11 | **0.002** |
| Working Class | 0.21 | 0.17 – 0.25 | **<0.001** | -0.44 | -0.48 – -0.41 | **<0.001** | -0.34 | -0.38 – -0.29 | **<0.001** |
| Rich | -0.00 | -0.04 – 0.03 | 0.819 | 0.05 | 0.02 – 0.09 | **0.004** | 0.08 | 0.04 – 0.12 | **<0.001** |
| Poor | -0.07 | -0.11 – -0.03 | **0.001** | -0.67 | -0.71 – -0.64 | **<0.001** | -0.36 | -0.40 – -0.32 | **<0.001** |
| Higher Educated | 0.16 | 0.12 – 0.20 | **<0.001** | -0.09 | -0.12 – -0.05 | **<0.001** | -0.15 | -0.19 – -0.11 | **<0.001** |
| Lower Educated | -0.03 | -0.07 – 0.01 | 0.170 | -0.62 | -0.65 – -0.58 | **<0.001** | -0.37 | -0.42 – -0.33 | **<0.001** |
| Politicians | 0.05 | 0.01 – 0.09 | **0.010** | 0.03 | -0.00 – 0.07 | 0.060 | 0.11 | 0.07 – 0.15 | **<0.001** |
| Doctors | 0.24 | 0.20 – 0.28 | **<0.001** | -0.15 | -0.18 – -0.11 | **<0.001** | -0.39 | -0.43 – -0.34 | **<0.001** |
| Elderly | -0.01 | -0.05 – 0.03 | 0.769 | -0.46 | -0.49 – -0.42 | **<0.001** | -0.32 | -0.36 – -0.27 | **<0.001** |
| Homeless | -0.28 | -0.32 – -0.24 | **<0.001** | -0.76 | -0.80 – -0.73 | **<0.001** | -0.48 | -0.52 – -0.44 | **<0.001** |
| Age centered | 0.00 | -0.00 – 0.00 | 0.948 | 0.00 | 0.00 – 0.00 | **0.002** | 0.00 | -0.00 – 0.00 | 0.162 |
| Male | 0.01 | -0.02 – 0.04 | 0.615 | -0.02 | -0.04 – 0.00 | 0.097 | 0.02 | -0.02 – 0.05 | 0.343 |
| Political Orientation centered | -0.01 | -0.02 – -0.00 | **0.001** | -0.00 | -0.01 – 0.00 | 0.458 | -0.01 | -0.01 – 0.00 | 0.130 |
| Participant state educated | -0.05 | -0.09 – -0.00 | **0.050** | -0.02 | -0.05 – 0.00 | 0.088 | -0.01 | -0.06 – 0.04 | 0.627 |
| **Random Effects** | | | | | | | | | |
| σ^2^ | 0.04 | | | 0.03 | | | 0.04 | | |
| τ_00_ | 0.01 _id_ | | | 0.00 _id_ | | | 0.01 _id_ | | |
| ICC | 0.20 | | | 0.06 | | | 0.19 | | |
| N | 191 _id_ | | | 191 _id_ | | | 191 _id_ | | |
| Observations | 2292 | | | 2292 | | | 2292 | | |
| Marginal R^2^ / Conditional R^2^ | 0.308 / 0.443 | | | 0.728 / 0.746 | | | 0.445 / 0.553 | | |

Table S 8. Communion and Agency by group and BSM (privately educated as reference group).

|  | **Communion** | | | | | **Agency** | | | | | | |  |  |
| --- | --- | --- | --- | --- | --- | --- | --- | --- | --- | --- | --- | --- | --- | --- |
| *Predictors* | *Estimates* | *CI* | | *p* | | | *Estimates* | | *CI* | | *p* | | |  |
| (Intercept) | 0.34 | | 0.29 – 0.39 | | **<0.001** | | | 0.73 | | 0.68 – 0.77 | | **<0.001** | | |
| State Educated | 0.34 | | 0.30 – 0.38 | | **<0.001** | | | -0.32 | | -0.36 – -0.28 | | **<0.001** | | |
| Upper Class | -0.07 | | -0.12 – -0.03 | | **0.001** | | | 0.08 | | 0.04 – 0.12 | | **<0.001** | | |
| Working Class | 0.32 | | 0.28 – 0.37 | | **<0.001** | | | -0.35 | | -0.39 – -0.30 | | **<0.001** | | |
| Rich | -0.08 | | -0.12 – -0.04 | | **<0.001** | | | 0.09 | | 0.05 – 0.13 | | **<0.001** | | |
| Poor | 0.34 | | 0.29 – 0.38 | | **<0.001** | | | -0.50 | | -0.54 – -0.46 | | **<0.001** | | |
| Higher Educated | 0.11 | | 0.07 – 0.15 | | **<0.001** | | | -0.09 | | -0.13 – -0.04 | | **<0.001** | | |
| Lower Educated | 0.34 | | 0.30 – 0.38 | | **<0.001** | | | -0.44 | | -0.48 – -0.39 | | **<0.001** | | |
| Politicians | -0.20 | | -0.25 – -0.16 | | **<0.001** | | | 0.12 | | 0.08 – 0.17 | | **<0.001** | | |
| Doctors | 0.37 | | 0.33 – 0.42 | | **<0.001** | | | -0.22 | | -0.26 – -0.17 | | **<0.001** | | |
| Elderly | 0.42 | | 0.38 – 0.47 | | **<0.001** | | | -0.42 | | -0.46 – -0.37 | | **<0.001** | | |
| Homeless | 0.30 | | 0.26 – 0.34 | | **<0.001** | | | -0.60 | | -0.64 – -0.55 | | **<0.001** | | |
| BSM | 0.06 | | 0.03 – 0.09 | | **<0.001** | | | -0.06 | | -0.09 – -0.04 | | **<0.001** | | |
| Age centered | -0.00 | | -0.00 – -0.00 | | **0.018** | | | 0.00 | | -0.00 – 0.00 | | 0.102 | | |
| Male | -0.01 | | -0.04 – 0.02 | | 0.473 | | | 0.00 | | -0.02 – 0.03 | | 0.900 | | |
| Political Orientation centered | -0.00 | | -0.01 – 0.01 | | 0.818 | | | -0.00 | | -0.01 – 0.00 | | 0.223 | | |
| Participant state educated | -0.01 | | -0.05 – 0.04 | | 0.780 | | | -0.02 | | -0.06 – 0.02 | | 0.285 | | |
| State Educated*BSM | -0.06 | | -0.09 – -0.02 | | **0.002** | | | 0.07 | | 0.04 – 0.11 | | **<0.001** | | |
| Upper Class*BSM | -0.05 | | -0.09 – -0.02 | | **0.004** | | | 0.05 | | 0.02 – 0.09 | | **0.004** | | |
| Working Class*BSM | -0.07 | | -0.11 – -0.04 | | **<0.001** | | | 0.07 | | 0.04 – 0.11 | | **<0.001** | | |
| Rich*BSM | -0.03 | | -0.07 – 0.00 | | 0.061 | | | 0.03 | | -0.01 – 0.06 | | 0.123 | | |
| Poor*BSM | -0.03 | | -0.07 – 0.00 | | 0.084 | | | 0.08 | | 0.04 – 0.11 | | **<0.001** | | |
| Higher Educated*BSM | -0.08 | | -0.12 – -0.04 | | **<0.001** | | | 0.06 | | 0.03 – 0.10 | | **<0.001** | | |
| Lower Educated*BSM | -0.06 | | -0.10 – -0.03 | | **0.001** | | | 0.08 | | 0.04 – 0.11 | | **<0.001** | | |
| Politicians*BSM | -0.04 | | -0.07 – -0.00 | | **0.036** | | | 0.02 | | -0.01 – 0.06 | | 0.164 | | |
| Doctors*BSM | -0.06 | | -0.10 – -0.03 | | **0.001** | | | 0.07 | | 0.04 – 0.11 | | **<0.001** | | |
| Elderly*BSM | -0.06 | | -0.10 – -0.03 | | **<0.001** | | | 0.07 | | 0.04 – 0.11 | | **<0.001** | | |
| Homeless*BSM | -0.07 | | -0.10 – -0.03 | | **<0.001** | | | 0.09 | | 0.06 – 0.13 | | **<0.001** | | |
| **Random Effects** | | | | | | | | | | | | |  |  |
| σ^2^ | 0.05 | | | | | 0.05 | | | | | | |  |  |
| τ_00_ | 0.01 _id_ | | | | | 0.00 _id_ | | | | | | |  |  |
| ICC | 0.11 | | | | | 0.08 | | | | | | |  |  |
| N | 191 _id_ | | | | | 191 _id_ | | | | | | |  |  |
| Observations | 2292 | | | | | 2292 | | | | | | |  |  |
| Marginal R^2^ / Conditional R^2^ | 0.460 / 0.517 | | | | | 0.547 / 0.582 | | | | | | |  |  |

Table S 9. Communion and Agency by group and BSM (privately educated as reference group).

|  | **Entitativity** | | | **Permeability** | | | **Status** | | |
| --- | --- | --- | --- | --- | --- | --- | --- | --- | --- |
| *Predictors* | *Estimates* | *CI* | *p* | *Estimates* | *CI* | *p* | *Estimates* | *CI* | *p* |
| (Intercept) | 0.65 | 0.58 – 0.73 | **<0.001** | 0.26 | 0.20 – 0.31 | **<0.001** | 0.84 | 0.80 – 0.87 | **<0.001** |
| State Educated | -0.22 | -0.27 – -0.18 | **<0.001** | 0.61 | 0.57 – 0.66 | **<0.001** | -0.31 | -0.34 – -0.28 | **<0.001** |
| Upper Class | 0.04 | -0.01 – 0.08 | 0.113 | -0.08 | -0.12 – -0.04 | **<0.001** | 0.05 | 0.02 – 0.08 | **0.001** |
| Working Class | -0.06 | -0.11 – -0.02 | **0.008** | 0.48 | 0.44 – 0.52 | **<0.001** | -0.46 | -0.49 – -0.42 | **<0.001** |
| Rich | -0.01 | -0.05 – 0.04 | 0.787 | -0.03 | -0.08 – 0.01 | 0.168 | 0.06 | 0.03 – 0.09 | **<0.001** |
| Poor | -0.25 | -0.29 – -0.20 | **<0.001** | 0.54 | 0.50 – 0.59 | **<0.001** | -0.71 | -0.74 – -0.68 | **<0.001** |
| Higher Educated | -0.09 | -0.13 – -0.04 | **<0.001** | 0.14 | 0.09 – 0.18 | **<0.001** | -0.02 | -0.05 – 0.01 | 0.262 |
| Lower Educated | -0.28 | -0.33 – -0.24 | **<0.001** | 0.42 | 0.38 – 0.47 | **<0.001** | -0.61 | -0.64 – -0.58 | **<0.001** |
| Politicians | 0.09 | 0.05 – 0.14 | **<0.001** | 0.07 | 0.02 – 0.11 | **0.004** | 0.00 | -0.03 – 0.03 | 0.878 |
| Doctors | 0.04 | -0.01 – 0.09 | 0.108 | -0.10 | -0.15 – -0.06 | **<0.001** | 0.01 | -0.02 – 0.04 | 0.649 |
| Elderly | -0.05 | -0.09 – 0.00 | 0.056 | 0.46 | 0.41 – 0.50 | **<0.001** | -0.39 | -0.42 – -0.36 | **<0.001** |
| Homeless | -0.16 | -0.20 – -0.11 | **<0.001** | 0.42 | 0.37 – 0.46 | **<0.001** | -0.76 | -0.79 – -0.73 | **<0.001** |
| BSM | -0.02 | -0.05 – 0.02 | 0.320 | 0.04 | 0.01 – 0.06 | **0.008** | -0.04 | -0.06 – -0.02 | **<0.001** |
| Age centered | 0.00 | 0.00 – 0.00 | **0.008** | 0.00 | -0.00 – 0.00 | 0.716 | 0.00 | 0.00 – 0.00 | **0.001** |
| Male | -0.01 | -0.06 – 0.03 | 0.583 | -0.03 | -0.06 – -0.00 | **0.046** | -0.03 | -0.05 – -0.01 | **0.002** |
| Political Orientation centered | 0.01 | -0.00 – 0.02 | 0.169 | -0.01 | -0.01 – -0.00 | **0.044** | -0.00 | -0.01 – 0.00 | 0.078 |
| Participant state educated | 0.08 | 0.01 – 0.15 | **0.021** | -0.01 | -0.05 – 0.03 | 0.577 | -0.01 | -0.04 – 0.01 | 0.373 |
| State Educated*BSM | 0.02 | -0.02 – 0.05 | 0.432 | -0.03 | -0.06 – 0.01 | 0.122 | 0.05 | 0.03 – 0.08 | **<0.001** |
| Upper Class*BSM | -0.02 | -0.05 – 0.02 | 0.375 | 0.00 | -0.03 – 0.04 | 0.921 | 0.02 | -0.00 – 0.05 | 0.102 |
| Working Class*BSM | 0.00 | -0.03 – 0.04 | 0.846 | -0.02 | -0.06 – 0.02 | 0.300 | 0.07 | 0.05 – 0.10 | **<0.001** |
| Rich*BSM | 0.01 | -0.03 – 0.04 | 0.714 | -0.00 | -0.04 – 0.04 | 0.951 | 0.02 | -0.01 – 0.04 | 0.151 |
| Poor*BSM | -0.00 | -0.04 – 0.03 | 0.847 | -0.07 | -0.10 – -0.03 | **<0.001** | 0.07 | 0.05 – 0.10 | **<0.001** |
| Higher Educated*BSM | 0.03 | -0.01 – 0.06 | 0.176 | 0.01 | -0.03 – 0.04 | 0.667 | 0.04 | 0.01 – 0.06 | **0.003** |
| Lower Educated*BSM | 0.00 | -0.03 – 0.04 | 0.854 | -0.07 | -0.11 – -0.03 | **<0.001** | 0.07 | 0.04 – 0.09 | **<0.001** |
| Politicians*BSM | 0.01 | -0.03 – 0.05 | 0.661 | -0.01 | -0.05 – 0.02 | 0.423 | 0.03 | 0.01 – 0.06 | **0.018** |
| Doctors*BSM | 0.02 | -0.01 – 0.06 | 0.211 | -0.02 | -0.05 – 0.02 | 0.313 | 0.04 | 0.02 – 0.07 | **0.001** |
| Elderly*BSM | 0.02 | -0.02 – 0.06 | 0.360 | -0.04 | -0.07 – -0.00 | **0.038** | 0.04 | 0.02 – 0.07 | **0.001** |
| Homeless*BSM | 0.03 | -0.01 – 0.07 | 0.126 | -0.05 | -0.09 – -0.01 | **0.007** | 0.06 | 0.03 – 0.08 | **<0.001** |
| **Random Effects** | | | | | | | | | |
| σ^2^ | 0.05 | | | 0.05 | | | 0.02 | | |
| τ_00_ | 0.02 _id_ | | | 0.01 _id_ | | | 0.00 _id_ | | |
| ICC | 0.29 | | | 0.10 | | | 0.07 | | |
| N | 191 _id_ | | | 191 _id_ | | | 191 _id_ | | |
| Observations | 2292 | | | 2292 | | | 2292 | | |
| Marginal R^2^ / Conditional R^2^ | 0.178 / 0.414 | | | 0.549 / 0.595 | | | 0.783 / 0.799 | | |

Table S 10. Interdependence, influence, and conflict by group and BSM (privately educated as reference group).

|  | **Interdependence** | | | **Influence** | | | **Conflict** | | |
| --- | --- | --- | --- | --- | --- | --- | --- | --- | --- |
| *Predictors* | *Estimates* | *CI* | *p* | *Estimates* | *CI* | *p* | *Estimates* | *CI* | *p* |
| (Intercept) | 0.41 | 0.35 – 0.46 | **<0.001** | 0.86 | 0.82 – 0.90 | **<0.001** | 0.65 | 0.59 – 0.70 | **<0.001** |
| State Educated | 0.20 | 0.16 – 0.24 | **<0.001** | -0.38 | -0.42 – -0.35 | **<0.001** | -0.38 | -0.42 – -0.34 | **<0.001** |
| Upper Class | -0.04 | -0.08 – 0.00 | 0.063 | 0.06 | 0.03 – 0.10 | **<0.001** | 0.07 | 0.03 – 0.11 | **0.001** |
| Working Class | 0.21 | 0.17 – 0.25 | **<0.001** | -0.44 | -0.47 – -0.41 | **<0.001** | -0.33 | -0.38 – -0.29 | **<0.001** |
| Rich | -0.00 | -0.04 – 0.03 | 0.811 | 0.05 | 0.02 – 0.09 | **0.003** | 0.08 | 0.04 – 0.12 | **<0.001** |
| Poor | -0.07 | -0.11 – -0.03 | **0.001** | -0.67 | -0.71 – -0.64 | **<0.001** | -0.36 | -0.40 – -0.31 | **<0.001** |
| Higher Educated | 0.16 | 0.12 – 0.20 | **<0.001** | -0.08 | -0.12 – -0.05 | **<0.001** | -0.15 | -0.19 – -0.11 | **<0.001** |
| Lower Educated | -0.03 | -0.07 – 0.01 | 0.162 | -0.62 | -0.65 – -0.58 | **<0.001** | -0.37 | -0.41 – -0.33 | **<0.001** |
| Politicians | 0.05 | 0.01 – 0.09 | **0.010** | 0.03 | -0.00 – 0.07 | 0.056 | 0.11 | 0.07 – 0.15 | **<0.001** |
| Doctors | 0.24 | 0.20 – 0.28 | **<0.001** | -0.15 | -0.18 – -0.11 | **<0.001** | -0.39 | -0.43 – -0.34 | **<0.001** |
| Elderly | -0.01 | -0.04 – 0.03 | 0.781 | -0.46 | -0.49 – -0.42 | **<0.001** | -0.32 | -0.36 – -0.27 | **<0.001** |
| Homeless | -0.28 | -0.32 – -0.24 | **<0.001** | -0.76 | -0.79 – -0.73 | **<0.001** | -0.48 | -0.52 – -0.44 | **<0.001** |
| BSM | 0.01 | -0.01 – 0.04 | 0.316 | -0.04 | -0.06 – -0.02 | **<0.001** | -0.09 | -0.11 – -0.06 | **<0.001** |
| Age centered | 0.00 | -0.00 – 0.00 | 0.903 | 0.00 | 0.00 – 0.00 | **0.002** | 0.00 | -0.00 – 0.00 | 0.235 |
| Male | 0.01 | -0.02 – 0.04 | 0.624 | -0.02 | -0.04 – 0.00 | 0.098 | 0.02 | -0.02 – 0.05 | 0.312 |
| Political Orientation centered | -0.01 | -0.02 – -0.00 | **0.001** | -0.00 | -0.01 – 0.00 | 0.454 | -0.00 | -0.01 – 0.01 | 0.594 |
| Participant state educated | -0.05 | -0.09 – 0.00 | 0.055 | -0.02 | -0.05 – 0.00 | 0.091 | -0.02 | -0.07 – 0.03 | 0.526 |
| State Educated*BSM | -0.03 | -0.06 – 0.00 | 0.056 | 0.05 | 0.02 – 0.08 | **<0.001** | 0.10 | 0.07 – 0.13 | **<0.001** |
| Upper Class*BSM | 0.01 | -0.02 – 0.04 | 0.467 | 0.02 | -0.01 – 0.05 | 0.182 | 0.03 | -0.00 – 0.07 | 0.058 |
| Working Class*BSM | -0.02 | -0.06 – 0.01 | 0.127 | 0.06 | 0.04 – 0.09 | **<0.001** | 0.10 | 0.06 – 0.13 | **<0.001** |
| Rich*BSM | -0.01 | -0.04 – 0.02 | 0.459 | 0.02 | -0.01 – 0.05 | 0.190 | 0.04 | 0.01 – 0.08 | **0.010** |
| Poor*BSM | -0.02 | -0.05 – 0.01 | 0.159 | 0.06 | 0.04 – 0.09 | **<0.001** | 0.07 | 0.03 – 0.10 | **<0.001** |
| Higher Educated*BSM | -0.01 | -0.04 – 0.02 | 0.495 | 0.04 | 0.01 – 0.06 | **0.012** | 0.08 | 0.04 – 0.11 | **<0.001** |
| Lower Educated*BSM | -0.03 | -0.06 – 0.00 | 0.098 | 0.07 | 0.04 – 0.10 | **<0.001** | 0.08 | 0.05 – 0.11 | **<0.001** |
| Politicians*BSM | -0.01 | -0.04 – 0.02 | 0.556 | 0.01 | -0.02 – 0.04 | 0.409 | 0.05 | 0.01 – 0.08 | **0.006** |
| Doctors*BSM | -0.00 | -0.03 – 0.03 | 0.920 | 0.06 | 0.03 – 0.09 | **<0.001** | 0.10 | 0.07 – 0.13 | **<0.001** |
| Elderly*BSM | 0.02 | -0.01 – 0.06 | 0.144 | 0.04 | 0.01 – 0.07 | **0.006** | 0.06 | 0.03 – 0.09 | **0.001** |
| Homeless*BSM | 0.01 | -0.03 – 0.04 | 0.745 | 0.05 | 0.02 – 0.08 | **0.001** | 0.08 | 0.05 – 0.12 | **<0.001** |
| **Random Effects** | | | | | | | | | |
| σ^2^ | 0.61 | | | 0.47 | | | 0.69 | | |
| τ_00_ | 0.16 _id_ | | | 0.03 _id_ | | | 0.16 _id_ | | |
| ICC | 0.20 | | | 0.07 | | | 0.19 | | |
| N | 191 _id_ | | | 191 _id_ | | | 191 _id_ | | |
| Observations | 2292 | | | 2292 | | | 2292 | | |
| Marginal R^2^ / Conditional R^2^ | 0.307 / 0.448 | | | 0.733 / 0.752 | | | 0.462 / 0.566 | | |

## Study 2

Table S 11. Correlations Study 2.

| Study 2 | BSM | Political Orientation | Level of Education | Own type of education | Children’s education |
| --- | --- | --- | --- | --- | --- |
| BSM | - |  |  |  |  |
| Political Orientation | .405*** | - |  |  |  |
| Level of Education | -.068+ | -.070+ | - |  |  |
| Own type of education | .051 | -.008 | -.134*** | - |  |
| Children’s education | .027 | -.039 | -.051 | .258*** | - |

Note. + indicates p < .10. * indicates p < .05, ** indicates p < .01. *** indicates p < .001.

Table S 12. Fixed-Effects ANOVA results.

| Criterion | Predictor | Sum of Squares | *df* | Mean Square | *F* | *p* | _partial_ η^2^ | _partial_ η^2^  90% CI  [LL, UL] |
| --- | --- | --- | --- | --- | --- | --- | --- | --- |
| Trust in Council | (Intercept) | 5457.37 | 1 | 5457.37 | 5170.12 | .000 |  |  |
|  | Private vs. Control | 10.65 | 1 | 10.65 | 10.09 | .002 | .02 | [.00, .04] |
|  | Private vs. State | 7.58 | 1 | 7.58 | 7.18 | .008 | .01 | [.00, .03] |
|  | Age | 2.35 | 1 | 2.35 | 2.22 | .137 | .00 | [.00, .02] |
|  | Political Orientation | 3.18 | 1 | 3.18 | 3.02 | .083 | .01 | [.00, .02] |
|  | Gender | 0.01 | 1 | 0.01 | 0.01 | .906 | .00 | [.00, .00] |
|  | Education Sector | 0.16 | 1 | 0.16 | 0.16 | .694 | .00 | [.00, .01] |
|  | Error | 623.84 | 591 | 1.06 |  |  |  |  |
| Representation | (Intercept) | 4339.85 | 1 | 4339.85 | 4103.51 | .000 |  |  |
|  | Private vs. Control | 18.51 | 1 | 18.51 | 17.50 | .000 | .03 | [.01, .05] |
|  | Private vs. State | 16.02 | 1 | 16.02 | 15.15 | .000 | .02 | [.01, .05] |
|  | Age | 0.23 | 1 | 0.23 | 0.22 | .640 | .00 | [.00, .01] |
|  | Political Orientation | 18.76 | 1 | 18.76 | 17.73 | .000 | .03 | [.01, .05] |
|  | Gender | 1.00 | 1 | 1.00 | 0.95 | .331 | .00 | [.00, .01] |
|  | Education Sector | 0.30 | 1 | 0.30 | 0.28 | .597 | .00 | [.00, .01] |
|  | Error | 625.04 | 591 | 1.06 |  |  |  |  |
| Communion | (Intercept) | 6037.96 | 1 | 6037.96 | 4844.55 | .000 |  |  |
|  | Private vs. Control | 87.58 | 1 | 87.58 | 70.27 | .000 | .11 | [.07, .15] |
|  | Private vs. State | 74.39 | 1 | 74.39 | 59.69 | .000 | .09 | [.06, .13] |
|  | Age | 0.50 | 1 | 0.50 | 0.40 | .528 | .00 | [.00, .01] |
|  | Political Orientation | 3.07 | 1 | 3.07 | 2.46 | .117 | .00 | [.00, .02] |
|  | Gender | 1.57 | 1 | 1.57 | 1.26 | .263 | .00 | [.00, .01] |
|  | Education Sector | 0.06 | 1 | 0.06 | 0.05 | .824 | .00 | [.00, .00] |
|  | Error | 736.59 | 591 | 1.25 |  |  |  |  |
| Agency | (Intercept) | 5969.44 | 1 | 5969.44 | 7167.39 | .000 |  |  |
|  | Private vs. Control | 0.07 | 1 | 0.07 | 0.08 | .780 | .00 | [.00, .01] |
|  | Private vs. State | 1.03 | 1 | 1.03 | 1.24 | .266 | .00 | [.00, .01] |
|  | Age | 0.03 | 1 | 0.03 | 0.04 | .844 | .00 | [.00, .00] |
|  | Political Orientation | 4.07 | 1 | 4.07 | 4.89 | .027 | .01 | [.00, .02] |
|  | Gender | 1.45 | 1 | 1.45 | 1.74 | .187 | .00 | [.00, .01] |
|  | Education Sector | 0.01 | 1 | 0.01 | 0.01 | .928 | .00 | [.00, .00] |
|  | Error | 492.22 | 591 | 0.83 |  |  |  |  |

*Note.* LL and UL represent the lower-limit and upper-limit of the partial η^2^ confidence interval, respectively.

Table S 13. IA Condition and BSM on Trust.

| Predictor | *b* | *b*  95% CI  [LL, UL] | *sr^2^* | *sr^2^*  95% CI  [LL, UL] | Fit |
| --- | --- | --- | --- | --- | --- |
| (Intercept) | 0.58** | [0.54, 0.62] |  |  |  |
| Private vs. Control | 0.05** | [0.02, 0.08] | .01 | [-.00, .03] |  |
| Private vs. State | 0.04* | [0.01, 0.08] | .01 | [-.01, .03] |  |
| Belief in School Meritocracy | 0.05** | [0.02, 0.07] | .02 | [-.00, .05] |  |
| Age | 0.00 | [-0.00, 0.00] | .00 | [-.01, .01] |  |
| Political Orientation | 0.00 | [-0.01, 0.01] | .00 | [-.00, .00] |  |
| Gender | -0.01 | [-0.04, 0.02] | .00 | [-.00, .00] |  |
| Education Sector | -0.01 | [-0.05, 0.03] | .00 | [-.00, .00] |  |
| Private vs. Control * BSM | -0.01 | [-0.04, 0.03] | .00 | [-.00, .00] |  |
| Private vs. State * BSM | -0.04* | [-0.07, -0.00] | .01 | [-.01, .02] |  |
|  |  |  |  |  | *R^2^*  = .064** |
|  |  |  |  |  | 95% CI[.02,.09] |
|  |  |  |  |  |  |

*Note.* A significant *b*-weight indicates the semi-partial correlation is also significant. *b* represents unstandardized regression weights. *sr^2^* represents the semi-partial correlation squared. *LL* and *UL* indicate the lower and upper limits of a confidence interval, respectively.
* indicates p < .05. ** indicates p < .01.

Table S 14. IA Condition and BSM on Representation.

| Predictor | *b* | *b*  95% CI  [LL, UL] | *sr^2^* | *sr^2^*  95% CI  [LL, UL] | Fit |
| --- | --- | --- | --- | --- | --- |
| (Intercept) | 0.49** | [0.45, 0.53] |  |  |  |
| Private vs. Control | 0.07** | [0.04, 0.10] | .03 | [.00, .05] |  |
| Private vs. State | 0.06** | [0.03, 0.10] | .02 | [-.00, .04] |  |
| Belief in School Meritocracy | 0.06** | [0.04, 0.08] | .03 | [.01, .06] |  |
| Age | 0.00 | [-0.00, 0.00] | .00 | [-.00, .00] |  |
| Political Orientation | 0.01* | [0.00, 0.01] | .01 | [-.01, .02] |  |
| Gender | -0.02 | [-0.05, 0.00] | .00 | [-.01, .01] |  |
| Education Sector | -0.01 | [-0.05, 0.02] | .00 | [-.00, .00] |  |
| Private vs. Control * BSM | -0.03 | [-0.07, 0.00] | .01 | [-.01, .02] |  |
| Private vs. State * BSM | -0.04* | [-0.08, -0.00] | .01 | [-.01, .02] |  |
|  |  |  |  |  | *R^2^*  = .106** |
|  |  |  |  |  | 95% CI[.05,.14] |
|  |  |  |  |  |  |

*Note.* A significant *b*-weight indicates the semi-partial correlation is also significant. *b* represents unstandardized regression weights. *sr^2^* represents the semi-partial correlation squared. *LL* and *UL* indicate the lower and upper limits of a confidence interval, respectively.
* indicates p < .05. ** indicates p < .01.

Table S 15. IA Condition and BSM on Agency.

| Predictor | *b* | *b*  95% CI  [LL, UL] | *sr^2^* | *sr^2^*  95% CI  [LL, UL] | Fit |
| --- | --- | --- | --- | --- | --- |
| (Intercept) | 0.66** | [0.62, 0.70] |  |  |  |
| Private vs. Control | 0.00 | [-0.03, 0.03] | .00 | [-.00, .00] |  |
| Private vs. State | 0.01 | [-0.01, 0.04] | .00 | [-.00, .01] |  |
| Belief in School Meritocracy | 0.05** | [0.02, 0.07] | .03 | [.00, .05] |  |
| Age | -0.00 | [-0.00, 0.00] | .00 | [-.00, .00] |  |
| Political Orientation | 0.00 | [-0.00, 0.01] | .00 | [-.00, .00] |  |
| Gender | -0.02 | [-0.05, 0.00] | .01 | [-.01, .02] |  |
| Education Sector | -0.00 | [-0.03, 0.03] | .00 | [-.00, .00] |  |
| Private vs. Control * BSM | -0.03 | [-0.06, 0.00] | .00 | [-.01, .02] |  |
| Private vs. State * BSM | -0.03* | [-0.07, -0.00] | .01 | [-.01, .02] |  |
|  |  |  |  |  | *R^2^*  = .044** |
|  |  |  |  |  | 95% CI[.01,.07] |
|  |  |  |  |  |  |

*Note.* A significant *b*-weight indicates the semi-partial correlation is also significant. *b* represents unstandardized regression weights. *sr^2^* represents the semi-partial correlation squared. *LL* and *UL* indicate the lower and upper limits of a confidence interval, respectively.
* indicates p < .05. ** indicates p < .01.

Table S 16. IA Condition and BSM on Communion.

| Predictor | *b* | *b*  95% CI  [LL, UL] | *sr^2^* | *sr^2^*  95% CI  [LL, UL] | Fit |
| --- | --- | --- | --- | --- | --- |
| (Intercept) | 0.53** | [0.48, 0.57] |  |  |  |
| Private vs. Control | 0.15** | [0.12, 0.19] | .10 | [.05, .14] |  |
| Private vs. State | 0.14** | [0.10, 0.18] | .08 | [.04, .12] |  |
| Belief in School Meritocracy | 0.07** | [0.04, 0.10] | .04 | [.01, .06] |  |
| Age | 0.00 | [-0.00, 0.00] | .00 | [-.00, .00] |  |
| Political Orientation | -0.00 | [-0.01, 0.01] | .00 | [-.00, .00] |  |
| Gender | -0.03 | [-0.06, 0.00] | .01 | [-.01, .02] |  |
| Education Sector | 0.00 | [-0.04, 0.04] | .00 | [-.00, .00] |  |
| Private vs. Control * BSM | -0.04* | [-0.08, -0.00] | .01 | [-.01, .02] |  |
| Private vs. State * BSM | -0.05* | [-0.09, -0.01] | .01 | [-.00, .02] |  |
|  |  |  |  |  | *R^2^*  = .177** |
|  |  |  |  |  | 95% CI[.11,.22] |
|  |  |  |  |  |  |

*Note.* A significant *b*-weight indicates the semi-partial correlation is also significant. *b* represents unstandardized regression weights. *sr^2^* represents the semi-partial correlation squared. *LL* and *UL* indicate the lower and upper limits of a confidence interval, respectively.
* indicates p < .05. ** indicates p < .01.

Table S 17. Interaction CV condition and Council Condition.

|  | **Overall Perception** | | | **Success in internship** | | | **Fit with council** | | |
| --- | --- | --- | --- | --- | --- | --- | --- | --- | --- |
| *Predictors* | *Estimates* | *CI* | *p* | *Estimates* | *CI* | *p* | *Estimates* | *CI* | *p* |
| (Intercept) | 5.41 | 5.31 – 5.50 | **<0.001** | 5.07 | 4.97 – 5.18 | **<0.001** | 4.91 | 4.80 – 5.02 | **<0.001** |
| State CV | 0.08 | 0.02 – 0.14 | **0.006** | 0.02 | -0.05 – 0.09 | 0.620 | -0.03 | -0.10 – 0.05 | 0.489 |
| Private vs. Other Council | -0.11 | -0.22 – 0.00 | 0.056 | -0.07 | -0.20 – 0.05 | 0.257 | -0.13 | -0.26 – -0.00 | **0.048** |
| State vs. Other Council | -0.09 | -0.20 – 0.03 | 0.132 | -0.03 | -0.16 – 0.09 | 0.620 | -0.13 | -0.26 – 0.00 | 0.052 |
| Age centered | 0.00 | -0.00 – 0.01 | 0.593 | 0.00 | -0.00 – 0.01 | 0.899 | 0.00 | -0.00 – 0.01 | 0.493 |
| LR centered | -0.01 | -0.04 – 0.01 | 0.343 | -0.03 | -0.06 – -0.00 | **0.027** | -0.03 | -0.06 – -0.00 | **0.048** |
| Male | -0.14 | -0.27 – -0.02 | **0.028** | -0.12 | -0.26 – 0.01 | 0.074 | -0.07 | -0.22 – 0.07 | 0.316 |
| CV * Private vs. Other Council | 0.06 | -0.04 – 0.15 | 0.228 | -0.05 | -0.16 – 0.07 | 0.444 | -0.08 | -0.21 – 0.04 | 0.198 |
| CV * State vs. Other Council | -0.04 | -0.14 – 0.05 | 0.349 | -0.12 | -0.23 – -0.01 | **0.040** | 0.05 | -0.08 – 0.17 | 0.454 |
| **Random Effects** | | | | | | | | | |
| σ^2^ | 0.40 | | | 0.63 | | | 0.76 | | |
| τ_00_ | 0.51 _id_ | | | 0.56 _id_ | | | 0.59 _id_ | | |
| ICC | 0.56 | | | 0.47 | | | 0.44 | | |
| N | 598 _id_ | | | 598 _id_ | | | 598 _id_ | | |
| Observations | 2392 | | | 2392 | | | 2392 | | |
| Marginal R^2^ / Conditional R^2^ | 0.015 / 0.566 | | | 0.013 / 0.477 | | | 0.015 / 0.445 | | |

Table S 17 continued.

|  | **Likelihood of Offer** | | | **Suited to work in Politics** | | | **Wage per Hour** | | |
| --- | --- | --- | --- | --- | --- | --- | --- | --- | --- |
| *Predictors* | *Estimates* | *CI* | *p* | *Estimates* | *CI* | *p* | *Estimates* | *CI* | *p* |
| (Intercept) | 4.84 | 4.73 – 4.96 | **<0.001** | 4.40 | 4.29 – 4.51 | **<0.001** | 13.08 | 12.06 – 14.10 | **<0.001** |
| State CV | -0.12 | -0.20 – -0.03 | **0.009** | 0.02 | -0.05 – 0.10 | 0.531 | 0.25 | -0.11 – 0.61 | 0.181 |
| Private vs. Other Council | -0.05 | -0.19 – 0.09 | 0.475 | -0.01 | -0.14 – 0.12 | 0.888 | -0.74 | -1.92 – 0.43 | 0.216 |
| State vs. Other Council | -0.03 | -0.17 – 0.11 | 0.636 | -0.01 | -0.14 – 0.12 | 0.926 | -0.48 | -1.66 – 0.69 | 0.420 |
| Age centered | 0.00 | -0.00 – 0.01 | 0.178 | 0.00 | -0.00 – 0.01 | 0.229 | -0.01 | -0.06 – 0.04 | 0.724 |
| LR centered | -0.02 | -0.05 – 0.01 | 0.190 | -0.02 | -0.05 – 0.01 | 0.115 | -0.06 | -0.35 – 0.24 | 0.692 |
| Male | -0.05 | -0.19 – 0.10 | 0.547 | -0.06 | -0.20 – 0.09 | 0.440 | 0.58 | -0.82 – 1.99 | 0.416 |
| CV * Private vs. Other Council | -0.18 | -0.32 – -0.03 | **0.018** | -0.01 | -0.13 – 0.12 | 0.886 | 0.01 | -0.59 – 0.60 | 0.978 |
| CV * State vs. Other Council | -0.03 | -0.17 – 0.11 | 0.693 | -0.06 | -0.18 – 0.06 | 0.346 | 0.03 | -0.56 – 0.61 | 0.931 |
| **Random Effects** | | | | | | | | | |
| σ^2^ | 1.00 | | | 0.73 | | | 15.58 | | |
| τ_00_ | 0.59 _id_ | | | 0.60 _id_ | | | 71.60 _id_ | | |
| ICC | 0.37 | | | 0.45 | | | 0.82 | | |
| N | 598 _id_ | | | 598 _id_ | | | 598 _id_ | | |
| Observations | 2392 | | | 2392 | | | 2392 | | |
| Marginal R^2^ / Conditional R^2^ | 0.012 / 0.376 | | | 0.005 / 0.453 | | | 0.004 / 0.822 | | |

Table S 18. Interaction CV condition, Council Condition and BSM.

|  | **Overall Perception** | | | **Success in internship** | | | **Fit with council** | | |
| --- | --- | --- | --- | --- | --- | --- | --- | --- | --- |
| *Predictors* | *Estimates* | *CI* | *p* | *Estimates* | *CI* | *p* | *Estimates* | *CI* | *p* |
| (Intercept) | 5.41 | 5.31 – 5.50 | **<0.001** | 5.07 | 4.96 – 5.17 | **<0.001** | 4.91 | 4.80 – 5.02 | **<0.001** |
| State CV | 0.08 | 0.03 – 0.14 | **0.004** | 0.02 | -0.05 – 0.09 | 0.546 | -0.02 | -0.10 – 0.05 | 0.562 |
| Private vs. Other Council | -0.11 | -0.22 – 0.01 | 0.067 | -0.07 | -0.20 – 0.05 | 0.267 | -0.13 | -0.26 – 0.00 | 0.053 |
| State vs. Other Council | -0.08 | -0.19 – 0.03 | 0.159 | -0.03 | -0.16 – 0.09 | 0.622 | -0.13 | -0.26 – 0.00 | 0.053 |
| BSM centered | 0.06 | -0.00 – 0.12 | 0.060 | -0.00 | -0.07 – 0.07 | 0.937 | 0.01 | -0.06 – 0.08 | 0.766 |
| age centered | 0.00 | -0.00 – 0.01 | 0.635 | 0.00 | -0.00 – 0.01 | 0.887 | 0.00 | -0.00 – 0.01 | 0.478 |
| LR centered | -0.02 | -0.05 – 0.01 | 0.185 | -0.03 | -0.06 – -0.00 | **0.042** | -0.04 | -0.07 – -0.00 | **0.037** |
| Male | -0.14 | -0.27 – -0.01 | **0.029** | -0.12 | -0.26 – 0.02 | 0.086 | -0.07 | -0.21 – 0.07 | 0.350 |
| CV * Private vs. Other Council | 0.05 | -0.04 – 0.14 | 0.286 | -0.05 | -0.17 – 0.07 | 0.416 | -0.09 | -0.21 – 0.04 | 0.183 |
| CV * State vs. Other Council | -0.05 | -0.14 – 0.04 | 0.285 | -0.12 | -0.23 – -0.01 | **0.040** | 0.05 | -0.08 – 0.17 | 0.472 |
| CV * BSM | -0.05 | -0.10 – -0.01 | **0.029** | 0.02 | -0.04 – 0.07 | 0.598 | 0.01 | -0.05 – 0.07 | 0.714 |
| Private vs. Other Council * BSM | -0.02 | -0.10 – 0.07 | 0.732 | -0.03 | -0.13 – 0.07 | 0.535 | -0.00 | -0.11 – 0.10 | 0.957 |
| State vs. Other Council * BSM | -0.02 | -0.11 – 0.08 | 0.709 | -0.03 | -0.14 – 0.07 | 0.542 | -0.06 | -0.17 – 0.06 | 0.330 |
| CV * Private vs. Other Council * BSM | 0.07 | -0.00 – 0.14 | 0.066 | 0.07 | -0.02 – 0.16 | 0.144 | 0.11 | 0.01 – 0.21 | **0.029** |
| CV * State vs. Other Council * BSM | 0.09 | 0.01 – 0.17 | **0.020** | 0.11 | 0.01 – 0.20 | **0.034** | 0.10 | -0.00 – 0.21 | 0.057 |
| **Random Effects** | | | | | | | | | |
| σ^2^ | 0.40 | | | 0.63 | | | 0.76 | | |
| τ_00_ | 0.51 _id_ | | | 0.57 _id_ | | | 0.59 _id_ | | |
| ICC | 0.56 | | | 0.47 | | | 0.44 | | |
| N | 598 _id_ | | | 598 _id_ | | | 598 _id_ | | |
| Observations | 2392 | | | 2392 | | | 2392 | | |
| Marginal R^2^ / Conditional R^2^ | 0.019 / 0.570 | | | 0.015 / 0.481 | | | 0.019 / 0.449 | | |

Table S 18 continued.

|  | **Likelihood of Offer** | | | **Suited to work in Politics** | | | **Wage per Hour** | | |
| --- | --- | --- | --- | --- | --- | --- | --- | --- | --- |
| *Predictors* | *Estimates* | *CI* | *p* | *Estimates* | *CI* | *p* | *Estimates* | *CI* | *p* |
| (Intercept) | 4.84 | 4.72 – 4.95 | **<0.001** | 4.39 | 4.28 – 4.50 | **<0.001** | 13.05 | 12.02 – 14.07 | **<0.001** |
| State CV | -0.11 | -0.20 – -0.02 | **0.013** | 0.03 | -0.05 – 0.10 | 0.462 | 0.26 | -0.10 – 0.62 | 0.152 |
| Private vs. Other Council | -0.05 | -0.19 – 0.09 | 0.492 | -0.01 | -0.14 – 0.13 | 0.939 | -0.76 | -1.94 – 0.42 | 0.206 |
| State vs. Other Council | -0.03 | -0.17 – 0.11 | 0.653 | -0.00 | -0.13 – 0.13 | 0.966 | -0.53 | -1.70 – 0.65 | 0.378 |
| BSM centered | 0.00 | -0.07 – 0.08 | 0.907 | 0.03 | -0.04 – 0.10 | 0.449 | -0.23 | -0.88 – 0.41 | 0.476 |
| age centered | 0.00 | -0.00 – 0.01 | 0.166 | 0.00 | -0.00 – 0.01 | 0.247 | -0.01 | -0.06 – 0.04 | 0.707 |
| LR centered | -0.02 | -0.06 – 0.01 | 0.154 | -0.02 | -0.06 – 0.01 | 0.181 | -0.00 | -0.32 – 0.32 | 0.997 |
| Male | -0.04 | -0.19 – 0.11 | 0.614 | -0.05 | -0.19 – 0.09 | 0.493 | 0.65 | -0.76 – 2.05 | 0.368 |
| CV * Private vs. Other Council | -0.18 | -0.32 – -0.03 | **0.016** | -0.02 | -0.14 – 0.11 | 0.770 | -0.02 | -0.62 – 0.57 | 0.936 |
| CV * State vs. Other Council | -0.03 | -0.17 – 0.11 | 0.676 | -0.07 | -0.19 – 0.05 | 0.278 | 0.01 | -0.57 – 0.59 | 0.977 |
| CV * BSM | 0.03 | -0.04 – 0.11 | 0.357 | -0.07 | -0.14 – -0.01 | **0.020** | -0.21 | -0.50 – 0.09 | 0.165 |
| Private vs. Other Council * BSM | -0.04 | -0.15 – 0.07 | 0.469 | -0.04 | -0.15 – 0.06 | 0.401 | 0.45 | -0.48 – 1.38 | 0.344 |
| State vs. Other Council * BSM | -0.02 | -0.14 – 0.10 | 0.717 | -0.07 | -0.19 – 0.04 | 0.205 | -0.15 | -1.16 – 0.85 | 0.765 |
| CV * Private vs. Other Council * BSM | 0.15 | 0.03 – 0.26 | **0.012** | 0.10 | -0.00 – 0.20 | 0.057 | 0.24 | -0.24 – 0.71 | 0.328 |
| CV * State vs. Other Council * BSM | 0.12 | 0.00 – 0.24 | **0.047** | 0.12 | 0.01 – 0.22 | **0.030** | 0.55 | 0.06 – 1.05 | **0.029** |
| **Random Effects** | | | | | | | | | |
| σ^2^ | 1.00 | | | 0.73 | | | 15.56 | | |
| τ_00_ | 0.59 _id_ | | | 0.61 _id_ | | | 71.58 _id_ | | |
| ICC | 0.37 | | | 0.45 | | | 0.82 | | |
| N | 598 _id_ | | | 598 _id_ | | | 598 _id_ | | |
| Observations | 2392 | | | 2392 | | | 2392 | | |
| Marginal R^2^ / Conditional R^2^ | 0.015 / 0.381 | | | 0.008 / 0.459 | | | 0.008 / 0.823 | | |

## Study 3

Table S 19. Correlations Study 3.

| Study 3 | BSM | Political Orientation | Level of Education | Own type of education | Children’s education |
| --- | --- | --- | --- | --- | --- |
| BSM | - |  |  |  |  |
| Political Orientation | .296*** | - |  |  |  |
| Level of Education | -.179*** | -.101* | - |  |  |
| Own type of education | .001 | .022 | -.083 | - |  |
| Children’s education | .207 | -.039 | -.057 | .246** | - |

Note. + indicates p < .10. * indicates p < .05, ** indicates p < .01. *** indicates p < .001.

Table S 20. Fixed-Effects ANOVA results

| Criterion | Predictor | Sum of Squares | *df* | Mean Square | *F* | *p* | _partial_ η^2^ | _partial_ η^2^  90% CI  [LL, UL] |
| --- | --- | --- | --- | --- | --- | --- | --- | --- |
| Trust | (Intercept) | 273.14 | 1 | 273.14 | 122.64 | .000 |  |  |
|  | Private vs. Control | 60.13 | 1 | 60.13 | 27.00 | .000 | .07 | [.03, .11] |
|  | Private vs. State | 96.71 | 1 | 96.71 | 43.42 | .000 | .10 | [.06, .15] |
|  | Age | 0.12 | 1 | 0.12 | 0.05 | .815 | .00 | [.00, .01] |
|  | Political Orientation | 5.72 | 1 | 5.72 | 2.57 | .110 | .01 | [.00, .03] |
|  | Gender | 0.29 | 1 | 0.29 | 0.13 | .721 | .00 | [.00, .01] |
|  | Education Sector | 0.10 | 1 | 0.10 | 0.05 | .831 | .00 | [.00, .01] |
|  | Error | 832.97 | 374 | 2.23 |  |  |  |  |
| Representation | (Intercept) | 219.89 | 1 | 219.89 | 68.41 | .000 |  |  |
|  | Private vs. Control | 332.01 | 1 | 332.01 | 103.28 | .000 | .22 | [.16, .27] |
|  | Private vs. State | 312.17 | 1 | 312.17 | 97.11 | .000 | .21 | [.15, .26] |
|  | Age | 0.43 | 1 | 0.43 | 0.13 | .715 | .00 | [.00, .01] |
|  | Political Orientation | 0.08 | 1 | 0.08 | 0.03 | .872 | .00 | [.00, .01] |
|  | Gender | 2.38 | 1 | 2.38 | 0.74 | .390 | .00 | [.00, .02] |
|  | Education Sector | 2.19 | 1 | 2.19 | 0.68 | .410 | .00 | [.00, .02] |
|  | Error | 1202.25 | 374 | 3.21 |  |  |  |  |

*Note.* LL and UL represent the lower-limit and upper-limit of the partial η^2^ confidence interval, respectively.

Table S 21. IA condition and BSM.

|  | Predictor | Sum  of  Squares | *df* | Mean  Square | *F* | *p* | _partial_ η^2^ | _partial_ η^2^  90% CI  [LL, UL] |
| --- | --- | --- | --- | --- | --- | --- | --- | --- |
| Trust | (Intercept) | 275.70 | 1 | 275.70 | 126.00 | .000 |  |  |
|  | Private vs. Control | 60.94 | 1 | 60.94 | 27.85 | .000 | .07 | [.03, .11] |
|  | Private vs. State | 95.94 | 1 | 95.94 | 43.85 | .000 | .11 | [.06, .16] |
|  | Belief in School Meritocracy | 9.45 | 1 | 9.45 | 4.32 | .038 | .01 | [.00, .04] |
|  | Age | 0.44 | 1 | 0.44 | 0.20 | .656 | .00 | [.00, .01] |
|  | Political Orientation | 1.04 | 1 | 1.04 | 0.48 | .491 | .00 | [.00, .01] |
|  | Gender | 0.82 | 1 | 0.82 | 0.38 | .540 | .00 | [.00, .01] |
|  | Education Sector | 0.06 | 1 | 0.06 | 0.03 | .872 | .00 | [.00, .01] |
|  | Private vs. Control * BSM | 0.00 | 1 | 0.00 | 0.00 | .968 | .00 | [.00, 1.00] |
|  | Private vs. State * BSM | 0.83 | 1 | 0.83 | 0.38 | .538 | .00 | [.00, .01] |
|  | Error | 811.77 | 371 | 2.19 |  |  |  |  |
| Representation | (Intercept) | 222.68 | 1 | 222.68 | 71.44 | .000 |  |  |
|  | Private vs. Control | 334.56 | 1 | 334.56 | 107.33 | .000 | .22 | [.17, .28] |
|  | Private vs. State | 309.40 | 1 | 309.40 | 99.26 | .000 | .21 | [.15, .27] |
|  | Belief in School Meritocracy | 21.16 | 1 | 21.16 | 6.79 | .010 | .02 | [.00, .05] |
|  | Age | 1.44 | 1 | 1.44 | 0.46 | .497 | .00 | [.00, .01] |
|  | Political Orientation | 4.74 | 1 | 4.74 | 1.52 | .218 | .00 | [.00, .02] |
|  | Gender | 1.18 | 1 | 1.18 | 0.38 | .539 | .00 | [.00, .01] |
|  | Education Sector | 1.90 | 1 | 1.90 | 0.61 | .435 | .00 | [.00, .02] |
|  | Private vs. Control * BSM | 0.25 | 1 | 0.25 | 0.08 | .779 | .00 | [.00, .01] |
|  | Private vs. State * BSM | 0.54 | 1 | 0.54 | 0.17 | .676 | .00 | [.00, .01] |
|  | Error | 1156.42 | 371 | 3.12 |  |  |  |  |

*Note.* LL and UL represent the lower-limit and upper-limit of the partial η^2^ confidence interval, respectively.

## Study 4

### *Table S 22.* Correlation Study 4.

| Study 4 | BSM | Political Orientation | Level of Education | Own type of education | Children’s education |
| --- | --- | --- | --- | --- | --- |
| BSM | - |  |  |  |  |
| Political Orientation | .471*** | - |  |  |  |
| Level of Education | -.028 | -.076+ | - |  |  |
| Own type of education | -.110** | -.095* | -.038 | - |  |
| Children’s education | -.058 | -.022 | -.036 | .198*** | - |

Note. + indicates p < .10. * indicates p < .05, ** indicates p < .01. *** indicates p < .001.

Table S 23. Fixed-Effects ANOVA results

| Criterion | Predictor | Sum of Squares | *df* | Mean Square | *F* | *p* | _partial_ η^2^ | _partial_ η^2^  90% CI  [LL, UL] |
| --- | --- | --- | --- | --- | --- | --- | --- | --- |
| Trust in Council | (Intercept) | 19.39 | 1 | 19.39 | 462.32 | .000 |  |  |
|  | Private vs. Merit | 0.10 | 1 | 0.10 | 2.42 | .120 | .00 | [.00, .02] |
|  | Private vs. State | 1.31 | 1 | 1.31 | 31.28 | .000 | .05 | [.02, .08] |
|  | Age | 0.01 | 1 | 0.01 | 0.26 | .609 | .00 | [.00, .01] |
|  | Political Orientation | 1.50 | 1 | 1.50 | 35.85 | .000 | .06 | [.03, .09] |
|  | Gender | 0.13 | 1 | 0.13 | 3.21 | .074 | .01 | [.00, .02] |
|  | Education Sector | 0.23 | 1 | 0.23 | 5.58 | .018 | .01 | [.00, .03] |
|  | Error | 25.16 | 600 | 0.04 |  |  |  |  |
| Representation Personal | (Intercept) | 8.51 | 1 | 8.51 | 170.05 | .000 |  |  |
|  | Private vs. Merit | 0.27 | 1 | 0.27 | 5.32 | .021 | .01 | [.00, .03] |
|  | Private vs. State | 2.04 | 1 | 2.04 | 40.64 | .000 | .06 | [.04, .10] |
|  | Age | 0.02 | 1 | 0.02 | 0.40 | .525 | .00 | [.00, .01] |
|  | Political Orientation | 1.78 | 1 | 1.78 | 35.49 | .000 | .06 | [.03, .09] |
|  | Gender | 0.01 | 1 | 0.01 | 0.21 | .650 | .00 | [.00, .01] |
|  | Education Sector | 0.45 | 1 | 0.45 | 8.99 | .003 | .01 | [.00, .03] |
|  | Error | 30.04 | 600 | 0.05 |  |  |  |  |
| Representation Society | (Intercept) | 12.35 | 1 | 12.35 | 308.00 | .000 |  |  |
|  | Private vs. Merit | 0.14 | 1 | 0.14 | 3.55 | .060 | .01 | [.00, .02] |
|  | Private vs. State | 1.04 | 1 | 1.04 | 25.89 | .000 | .04 | [.02, .07] |
|  | Age | 0.00 | 1 | 0.00 | 0.07 | .798 | .00 | [.00, .00] |
|  | Political Orientation | 0.48 | 1 | 0.48 | 11.95 | .001 | .02 | [.01, .04] |
|  | Gender | 0.05 | 1 | 0.05 | 1.23 | .267 | .00 | [.00, .01] |
|  | Education Sector | 0.34 | 1 | 0.34 | 8.42 | .004 | .01 | [.00, .03] |
|  | Error | 24.06 | 600 | 0.04 |  |  |  |  |

*Note.* LL and UL represent the lower-limit and upper-limit of the partial η^2^ confidence interval, respectively.

Table S 18 continued.

| Criterion | Predictor | Sum of Squares | *df* | Mean Square | *F* | *p* | _partial_ η^2^ | _partial_ η^2^  90% CI  [LL, UL] |
| --- | --- | --- | --- | --- | --- | --- | --- | --- |
| Representation Politicians | (Intercept) | 21.35 | 1 | 21.35 | 485.54 | .000 |  |  |
|  | Private vs. Merit | 0.01 | 1 | 0.01 | 0.17 | .678 | .00 | [.00, .01] |
|  | Private vs. State | 0.79 | 1 | 0.79 | 17.95 | .000 | .03 | [.01, .05] |
|  | Age | 0.07 | 1 | 0.07 | 1.65 | .199 | .00 | [.00, .01] |
|  | Political Orientation | 0.00 | 1 | 0.00 | 0.01 | .907 | .00 | [.00, .00] |
|  | Gender | 0.15 | 1 | 0.15 | 3.46 | .063 | .01 | [.00, .02] |
|  | Education Sector | 0.07 | 1 | 0.07 | 1.60 | .206 | .00 | [.00, .01] |
|  | Error | 26.34 | 599 | 0.04 |  |  |  |  |
| Deservingness | (Intercept) | 18.68 | 1 | 18.68 | 443.13 | .000 |  |  |
|  | Private vs. Merit | 0.73 | 1 | 0.73 | 17.27 | .000 | .03 | [.01, .05] |
|  | Private vs. State | 1.55 | 1 | 1.55 | 36.85 | .000 | .06 | [.03, .09] |
|  | Age | 0.01 | 1 | 0.01 | 0.22 | .636 | .00 | [.00, .01] |
|  | Political Orientation | 1.11 | 1 | 1.11 | 26.37 | .000 | .04 | [.02, .07] |
|  | Gender | 0.28 | 1 | 0.28 | 6.65 | .010 | .01 | [.00, .03] |
|  | Education Sector | 0.24 | 1 | 0.24 | 5.72 | .017 | .01 | [.00, .03] |
|  | Error | 25.29 | 600 | 0.04 |  |  |  |  |

*Note.* LL and UL represent the lower-limit and upper-limit of the partial η^2^ confidence interval, respectively.

Table S 24. IA condition and BSM.

|  | Predictor | Sum  of  Squares | *df* | Mean  Square | *F* | *p* | _partial_ η^2^ | _partial_ η^2^  90% CI  [LL, UL] |
| --- | --- | --- | --- | --- | --- | --- | --- | --- |
| Trust | (Intercept) | 19.14 | 1 | 19.14 | 484.32 | .000 |  |  |
|  | Private vs. Merit | 0.11 | 1 | 0.11 | 2.81 | .094 | .00 | [.00, .02] |
|  | Private vs. State | 1.22 | 1 | 1.22 | 30.84 | .000 | .05 | [.02, .08] |
|  | Belief in School Meritocracy | 0.31 | 1 | 0.31 | 7.96 | .005 | .01 | [.00, .03] |
|  | Age | 0.00 | 1 | 0.00 | 0.08 | .777 | .00 | [.00, .01] |
|  | Political Orientation | 0.41 | 1 | 0.41 | 10.37 | .001 | .02 | [.00, .04] |
|  | Gender | 0.20 | 1 | 0.20 | 5.06 | .025 | .01 | [.00, .02] |
|  | Education Sector | 0.15 | 1 | 0.15 | 3.80 | .052 | .01 | [.00, .02] |
|  | Private vs. Merit * BSM | 0.19 | 1 | 0.19 | 4.82 | .029 | .01 | [.00, .02] |
|  | Private vs. State * BSM | 0.03 | 1 | 0.03 | 0.73 | .394 | .00 | [.00, .01] |
|  | Error | 23.59 | 597 | 0.04 |  |  |  |  |
| Representation Personal | (Intercept) | 8.37 | 1 | 8.37 | 173.51 | .000 |  |  |
|  | Private vs. Merit | 0.28 | 1 | 0.28 | 5.79 | .016 | .01 | [.00, .03] |
|  | Private vs. State | 0.25 | 1 | 0.25 | 5.13 | .024 | .01 | [.00, .02] |
|  | Belief in School Meritocracy | 1.93 | 1 | 1.93 | 39.93 | .000 | .06 | [.03, .10] |
|  | Age | 0.03 | 1 | 0.03 | 0.71 | .398 | .00 | [.00, .01] |
|  | Political Orientation | 0.62 | 1 | 0.62 | 12.77 | .000 | .02 | [.01, .04] |
|  | Gender | 0.03 | 1 | 0.03 | 0.64 | .423 | .00 | [.00, .01] |
|  | Education Sector | 0.34 | 1 | 0.34 | 7.08 | .008 | .01 | [.00, .03] |
|  | Private vs. Merit * BSM | 0.15 | 1 | 0.15 | 3.02 | .083 | .01 | [.00, .02] |
|  | Private vs. State * BSM | 0.02 | 1 | 0.02 | 0.38 | .538 | .00 | [.00, .01] |
|  | Error | 28.81 | 597 | 0.05 |  |  |  |  |

*Note.* LL and UL represent the lower-limit and upper-limit of the partial η^2^ confidence interval, respectively.

*Table S 19 continued.*

|  | Predictor | Sum  of  Squares | *df* | Mean  Square | *F* | *p* | _partial_ η^2^ | _partial_ η^2^  90% CI  [LL, UL] |
| --- | --- | --- | --- | --- | --- | --- | --- | --- |
| Representation Society | (Intercept) | 12.25 | 1 | 12.25 | 312.27 | .000 |  |  |
|  | Private vs. Merit | 0.15 | 1 | 0.15 | 3.79 | .052 | .01 | [.00, .02] |
|  | Private vs. State | 0.10 | 1 | 0.10 | 2.47 | .116 | .00 | [.00, .02] |
|  | Belief in School Meritocracy | 0.97 | 1 | 0.97 | 24.83 | .000 | .04 | [.02, .07] |
|  | Age | 0.00 | 1 | 0.00 | 0.00 | .947 | .00 | [.00, 1.00] |
|  | Political Orientation | 0.10 | 1 | 0.10 | 2.64 | .105 | .00 | [.00, .02] |
|  | Gender | 0.02 | 1 | 0.02 | 0.61 | .433 | .00 | [.00, .01] |
|  | Education Sector | 0.27 | 1 | 0.27 | 6.82 | .009 | .01 | [.00, .03] |
|  | Private vs. Merit * BSM | 0.10 | 1 | 0.10 | 2.44 | .119 | .00 | [.00, .02] |
|  | Private vs. State * BSM | 0.00 | 1 | 0.00 | 0.01 | .923 | .00 | [.00, .00] |
|  | Error | 23.41 | 597 | 0.04 |  |  |  |  |
| Representation Politicians | (Intercept) | 21.41 | 1 | 21.41 | 487.59 | .000 |  |  |
|  | Private vs. Merit | 0.01 | 1 | 0.01 | 0.19 | .663 | .00 | [.00, .01] |
|  | Private vs. State | 0.01 | 1 | 0.01 | 0.12 | .732 | .00 | [.00, .01] |
|  | Belief in School Meritocracy | 0.82 | 1 | 0.82 | 18.71 | .000 | .03 | [.01, .06] |
|  | Age | 0.06 | 1 | 0.06 | 1.36 | .245 | .00 | [.00, .01] |
|  | Political Orientation | 0.01 | 1 | 0.01 | 0.13 | .717 | .00 | [.00, .01] |
|  | Gender | 0.12 | 1 | 0.12 | 2.70 | .101 | .00 | [.00, .02] |
|  | Education Sector | 0.06 | 1 | 0.06 | 1.47 | .227 | .00 | [.00, .01] |
|  | Private vs. Merit * BSM | 0.01 | 1 | 0.01 | 0.12 | .733 | .00 | [.00, .01] |
|  | Private vs. State * BSM | 0.13 | 1 | 0.13 | 2.95 | .086 | .00 | [.00, .02] |
|  | Error | 26.17 | 596 | 0.04 |  |  |  |  |

*Note.* LL and UL represent the lower-limit and upper-limit of the partial η^2^ confidence interval, respectively.

*Table S 19 continued.*

|  | Predictor | Sum  of  Squares | *df* | Mean  Square | *F* | *p* | _partial_ η^2^ | _partial_ η^2^  90% CI  [LL, UL] |
| --- | --- | --- | --- | --- | --- | --- | --- | --- |
| Deservingness | (Intercept) | 18.41 | 1 | 18.41 | 459.39 | .000 |  |  |
|  | Private vs. Merit | 0.75 | 1 | 0.75 | 18.65 | .000 | .03 | [.01, .06] |
|  | Private vs. State | 0.46 | 1 | 0.46 | 11.52 | .001 | .02 | [.01, .04] |
|  | Belief in School Meritocracy | 1.47 | 1 | 1.47 | 36.66 | .000 | .06 | [.03, .09] |
|  | Age | 0.02 | 1 | 0.02 | 0.44 | .509 | .00 | [.00, .01] |
|  | Political Orientation | 0.26 | 1 | 0.26 | 6.42 | .012 | .01 | [.00, .03] |
|  | Gender | 0.34 | 1 | 0.34 | 8.47 | .004 | .01 | [.00, .03] |
|  | Education Sector | 0.16 | 1 | 0.16 | 4.08 | .044 | .01 | [.00, .02] |
|  | Private vs. Merit * BSM | 0.06 | 1 | 0.06 | 1.42 | .233 | .00 | [.00, .01] |
|  | Private vs. State * BSM | 0.14 | 1 | 0.14 | 3.61 | .058 | .01 | [.00, .02] |
|  | Error | 23.93 | 597 | 0.04 |  |  |  |  |

*Note.* LL and UL represent the lower-limit and upper-limit of the partial η^2^ confidence interval, respectively.

# Subgroup Analysis – all following analyses only include state educated participants

## Study 1a

### Table S 25. *Main effect condition or Communion, Agency, Toxicity, and Trust.*

|  | **Communion** | | | **Agency** | | | **Toxicity** | | | **Trust** | | |
| --- | --- | --- | --- | --- | --- | --- | --- | --- | --- | --- | --- | --- |
| *Predictors* | *Estimates* | *CI* | *p* | *Estimates* | *CI* | *p* | *Estimates* | *CI* | *p* | *Estimates* | *CI* | *p* |
| (Intercept) | 0.41 | 0.39 – 0.42 | **<0.001** | 0.38 | 0.36 – 0.40 | **<0.001** | 0.17 | 0.15 – 0.20 | **<0.001** | 0.53 | 0.50 – 0.56 | **<0.001** |
| Privately educated | -0.06 | -0.08 – -0.05 | **<0.001** | 0.06 | 0.04 – 0.07 | **<0.001** | 0.10 | 0.08 – 0.11 | **<0.001** | -0.10 | -0.11 – -0.08 | **<0.001** |
| Age (centred) | -0.00 | -0.00 – 0.00 | 0.754 | 0.00 | -0.00 – 0.00 | 0.609 | -0.00 | -0.00 – -0.00 | **0.047** | -0.00 | -0.00 – 0.00 | 0.461 |
| Male | -0.01 | -0.03 – 0.02 | 0.593 | -0.01 | -0.03 – 0.02 | 0.694 | 0.02 | -0.01 – 0.05 | 0.256 | -0.04 | -0.08 – 0.01 | 0.086 |
| LR (centred) | 0.01 | 0.00 – 0.01 | **0.003** | 0.00 | -0.00 – 0.01 | 0.688 | -0.01 | -0.01 – 0.00 | 0.136 | 0.01 | 0.00 – 0.02 | **0.014** |
| **Random Effects** | | | | | | | | | | | | |
| σ^2^ | 0.02 | | | 0.01 | | | 0.01 | | | 0.02 | | |
| τ_00_ | 0.00 _id_ | | | 0.01 _id_ | | | 0.01 _id_ | | | 0.02 _id_ | | |
| ICC | 0.23 | | | 0.34 | | | 0.44 | | | 0.44 | | |
| N | 173 _id_ | | | 173 _id_ | | | 173 _id_ | | | 173 _id_ | | |
| Observations | 1037 | | | 1038 | | | 1038 | | | 1038 | | |
| Marginal R^2^ / Conditional R^2^ | 0.063 / 0.275 | | | 0.048 / 0.372 | | | 0.128 / 0.515 | | | 0.083 / 0.482 | | |

### Table S 26*. IA Condition and Belief in School Meritocracy for Communion, Agency, Toxicity, and Trust.*

|  | **Communion** | | | **Agency** | | | **Toxicity** | | | **Trust** | | |
| --- | --- | --- | --- | --- | --- | --- | --- | --- | --- | --- | --- | --- |
| *Predictors* | *Estimates* | *CI* | *p* | *Estimates* | *CI* | *p* | *Estimates* | *CI* | *p* | *Estimates* | *CI* | *p* |
| (Intercept) | 0.41 | 0.39 – 0.42 | **<0.001** | 0.38 | 0.36 – 0.40 | **<0.001** | 0.17 | 0.15 – 0.20 | **<0.001** | 0.53 | 0.50 – 0.56 | **<0.001** |
| Privately educated | -0.06 | -0.08 – -0.05 | **<0.001** | 0.06 | 0.04 – 0.07 | **<0.001** | 0.10 | 0.09 – 0.11 | **<0.001** | -0.10 | -0.12 – -0.08 | **<0.001** |
| BSM (centred) | 0.01 | -0.00 – 0.03 | 0.110 | 0.02 | -0.00 – 0.04 | 0.065 | 0.01 | -0.01 – 0.03 | 0.520 | 0.02 | -0.01 – 0.05 | 0.195 |
| IA | 0.02 | 0.00 – 0.04 | **0.017** | 0.00 | -0.01 – 0.02 | 0.737 | -0.02 | -0.04 – -0.01 | **0.003** | 0.02 | 0.00 – 0.04 | **0.018** |
| Age (centred) | -0.00 | -0.00 – 0.00 | 0.678 | 0.00 | -0.00 – 0.00 | 0.652 | -0.00 | -0.00 – -0.00 | **0.049** | -0.00 | -0.00 – 0.00 | 0.416 |
| Male | -0.01 | -0.04 – 0.02 | 0.434 | -0.01 | -0.03 – 0.02 | 0.573 | 0.02 | -0.01 – 0.05 | 0.247 | -0.04 | -0.08 – 0.00 | 0.057 |
| LR (centred) | 0.00 | -0.00 – 0.01 | 0.235 | -0.00 | -0.01 – 0.00 | 0.483 | -0.00 | -0.01 – 0.00 | 0.261 | 0.01 | -0.00 – 0.02 | 0.282 |
| σ^2^ | 0.02 | | | 0.01 | | | 0.01 | | | 0.02 | | |
| τ_00_ | 0.00 _id_ | | | 0.01 _id_ | | | 0.01 _id_ | | | 0.02 _id_ | | |
| ICC | 0.21 | | | 0.33 | | | 0.45 | | | 0.43 | | |
| N | 173 _id_ | | | 173 _id_ | | | 173 _id_ | | | 173 _id_ | | |
| Observations | 1037 | | | 1038 | | | 1038 | | | 1212 | | |
| Marginal R^2^ / Conditional R^2^ | 0.084 / 0.280 | | | 0.059 / 0.373 | | | 0.132 / 0.520 | | | 0.111 / 0.489 | | |

*.*

Study 1b

Table S 27. Communion and Agency (privately educated as reference group)

|  | **Communion** | | | **Agency** | | |
| --- | --- | --- | --- | --- | --- | --- |
| *Predictors* | *Estimates* | *CI* | *p* | *Estimates* | *CI* | *p* |
| (Intercept) | 0.33 | 0.29 – 0.37 | **<0.001** | 0.72 | 0.68 – 0.75 | **<0.001** |
| State Educated | 0.35 | 0.31 – 0.40 | **<0.001** | -0.34 | -0.38 – -0.29 | **<0.001** |
| Upper Class | -0.07 | -0.11 – -0.02 | **0.006** | 0.06 | 0.02 – 0.11 | **0.007** |
| Working Class | 0.35 | 0.30 – 0.39 | **<0.001** | -0.37 | -0.41 – -0.32 | **<0.001** |
| Rich | -0.08 | -0.12 – -0.03 | **0.002** | 0.08 | 0.03 – 0.13 | **0.001** |
| Poor | 0.37 | 0.32 – 0.41 | **<0.001** | -0.52 | -0.56 – -0.47 | **<0.001** |
| Higher Educated | 0.11 | 0.07 – 0.16 | **<0.001** | -0.09 | -0.14 – -0.04 | **<0.001** |
| Lower Educated | 0.36 | 0.31 – 0.40 | **<0.001** | -0.46 | -0.50 – -0.41 | **<0.001** |
| Politicians | -0.19 | -0.24 – -0.15 | **<0.001** | 0.11 | 0.06 – 0.16 | **<0.001** |
| Doctors | 0.38 | 0.33 – 0.42 | **<0.001** | -0.22 | -0.27 – -0.18 | **<0.001** |
| Elderly | 0.44 | 0.39 – 0.48 | **<0.001** | -0.43 | -0.48 – -0.39 | **<0.001** |
| Homeless | 0.31 | 0.26 – 0.36 | **<0.001** | -0.61 | -0.66 – -0.56 | **<0.001** |
| Age centered | -0.00 | -0.00 – -0.00 | **0.049** | 0.00 | -0.00 – 0.00 | 0.078 |
| Male | -0.02 | -0.05 – 0.01 | 0.159 | 0.00 | -0.02 – 0.03 | 0.889 |
| Political Orientation centered | 0.00 | -0.00 – 0.01 | 0.751 | -0.01 | -0.01 – 0.00 | 0.053 |
| **Random Effects** | | | | | | |
| σ^2^ | 0.05 | | | 0.05 | | |
| τ_00_ | 0.01 _id_ | | | 0.00 _id_ | | |
| ICC | 0.10 | | | 0.07 | | |
| N | 166 _id_ | | | 166 _id_ | | |
| Observations | 1992 | | | 1992 | | |
| Marginal R^2^ / Conditional R^2^ | 0.465 / 0.520 | | | 0.548 / 0.581 | | |

Table S 28. Group Entitativity, Permeability and Status (privately educated as reference group).

|  | **Entitativity** | | | **Permeability** | | | **Status** | | |
| --- | --- | --- | --- | --- | --- | --- | --- | --- | --- |
| *Predictors* | *Estimates* | *CI* | *p* | *Estimates* | *CI* | *p* | *Estimates* | *CI* | *p* |
| (Intercept) | 0.74 | 0.69 – 0.79 | **<0.001** | 0.23 | 0.19 – 0.27 | **<0.001** | 0.83 | 0.81 – 0.86 | **<0.001** |
| State Educated | -0.23 | -0.28 – -0.18 | **<0.001** | 0.62 | 0.57 – 0.67 | **<0.001** | -0.33 | -0.36 – -0.29 | **<0.001** |
| Upper Class | 0.04 | -0.01 – 0.09 | 0.157 | -0.08 | -0.13 – -0.03 | **0.001** | 0.05 | 0.01 – 0.08 | **0.006** |
| Working Class | -0.08 | -0.13 – -0.03 | **0.003** | 0.50 | 0.45 – 0.54 | **<0.001** | -0.47 | -0.50 – -0.44 | **<0.001** |
| Rich | -0.00 | -0.05 – 0.05 | 0.906 | -0.03 | -0.08 – 0.02 | 0.215 | 0.06 | 0.02 – 0.09 | **0.001** |
| Poor | -0.25 | -0.30 – -0.20 | **<0.001** | 0.55 | 0.51 – 0.60 | **<0.001** | -0.73 | -0.76 – -0.69 | **<0.001** |
| Higher Educated | -0.09 | -0.14 – -0.04 | **0.001** | 0.14 | 0.10 – 0.19 | **<0.001** | -0.02 | -0.05 – 0.02 | 0.304 |
| Lower Educated | -0.28 | -0.33 – -0.23 | **<0.001** | 0.43 | 0.39 – 0.48 | **<0.001** | -0.63 | -0.67 – -0.60 | **<0.001** |
| Politicians | 0.09 | 0.04 – 0.14 | **0.001** | 0.06 | 0.01 – 0.11 | **0.013** | 0.00 | -0.03 – 0.03 | 1.000 |
| Doctors | 0.03 | -0.02 – 0.08 | 0.194 | -0.10 | -0.15 – -0.05 | **<0.001** | 0.00 | -0.03 – 0.03 | 0.964 |
| Elderly | -0.04 | -0.09 – 0.01 | 0.099 | 0.46 | 0.42 – 0.51 | **<0.001** | -0.41 | -0.44 – -0.38 | **<0.001** |
| Homeless | -0.16 | -0.21 – -0.11 | **<0.001** | 0.44 | 0.39 – 0.49 | **<0.001** | -0.77 | -0.80 – -0.74 | **<0.001** |
| Age centered | 0.00 | 0.00 – 0.00 | **0.029** | 0.00 | -0.00 – 0.00 | 0.994 | 0.00 | 0.00 – 0.00 | **0.006** |
| Male | -0.02 | -0.07 – 0.03 | 0.365 | -0.02 | -0.05 – 0.01 | 0.225 | -0.03 | -0.04 – -0.01 | **0.005** |
| Political Orientation centered | 0.01 | -0.00 – 0.02 | 0.058 | -0.01 | -0.01 – 0.00 | 0.065 | -0.00 | -0.01 – 0.00 | 0.087 |
| **Random Effects** | | | | | | | | | |
| σ^2^ | 0.05 | | | 0.05 | | | 0.02 | | |
| τ_00_ | 0.02 _id_ | | | 0.01 _id_ | | | 0.00 _id_ | | |
| ICC | 0.26 | | | 0.10 | | | 0.07 | | |
| N | 166 _id_ | | | 166 _id_ | | | 166 _id_ | | |
| Observations | 1992 | | | 1992 | | | 1992 | | |
| Marginal R^2^ / Conditional R^2^ | 0.174 / 0.391 | | | 0.559 / 0.604 | | | 0.791 / 0.805 | | |

Table S 29. Interdependence, Influence and Conflict (privately educated as reference group).

|  | **Interdependence** | | | **Influence** | | | **Conflict** | | |
| --- | --- | --- | --- | --- | --- | --- | --- | --- | --- |
| *Predictors* | *Estimates* | *CI* | *p* | *Estimates* | *CI* | *p* | *Estimates* | *CI* | *p* |
| (Intercept) | 0.35 | 0.31 – 0.38 | **<0.001** | 0.85 | 0.82 – 0.88 | **<0.001** | 0.65 | 0.61 – 0.69 | **<0.001** |
| State Educated | 0.22 | 0.18 – 0.27 | **<0.001** | -0.40 | -0.44 – -0.37 | **<0.001** | -0.41 | -0.46 – -0.37 | **<0.001** |
| Upper Class | -0.03 | -0.07 – 0.01 | 0.139 | 0.05 | 0.02 – 0.09 | **0.005** | 0.05 | 0.01 – 0.10 | **0.020** |
| Working Class | 0.24 | 0.20 – 0.28 | **<0.001** | -0.46 | -0.49 – -0.42 | **<0.001** | -0.37 | -0.42 – -0.33 | **<0.001** |
| Rich | 0.01 | -0.03 – 0.05 | 0.698 | 0.04 | 0.01 – 0.08 | **0.022** | 0.07 | 0.03 – 0.12 | **0.002** |
| Poor | -0.05 | -0.09 – -0.00 | **0.032** | -0.69 | -0.73 – -0.65 | **<0.001** | -0.39 | -0.44 – -0.35 | **<0.001** |
| Higher Educated | 0.16 | 0.12 – 0.20 | **<0.001** | -0.09 | -0.12 – -0.05 | **<0.001** | -0.17 | -0.21 – -0.12 | **<0.001** |
| Lower Educated | -0.01 | -0.05 – 0.03 | 0.597 | -0.64 | -0.67 – -0.60 | **<0.001** | -0.41 | -0.45 – -0.36 | **<0.001** |
| Politicians | 0.06 | 0.02 – 0.10 | **0.005** | 0.02 | -0.01 – 0.06 | 0.186 | 0.10 | 0.05 – 0.14 | **<0.001** |
| Doctors | 0.25 | 0.21 – 0.29 | **<0.001** | -0.16 | -0.20 – -0.13 | **<0.001** | -0.41 | -0.46 – -0.37 | **<0.001** |
| Elderly | -0.00 | -0.04 – 0.04 | 0.888 | -0.48 | -0.52 – -0.44 | **<0.001** | -0.35 | -0.39 – -0.30 | **<0.001** |
| Homeless | -0.27 | -0.31 – -0.23 | **<0.001** | -0.78 | -0.82 – -0.75 | **<0.001** | -0.52 | -0.56 – -0.47 | **<0.001** |
| Age centered | 0.00 | -0.00 – 0.00 | 0.407 | 0.00 | 0.00 – 0.00 | **0.004** | 0.00 | -0.00 – 0.00 | 0.260 |
| Male | 0.01 | -0.02 – 0.05 | 0.440 | -0.01 | -0.03 – 0.01 | 0.262 | 0.03 | -0.00 – 0.07 | 0.082 |
| Political Orientation centered | -0.01 | -0.02 – -0.01 | **<0.001** | -0.00 | -0.01 – 0.00 | 0.223 | -0.01 | -0.01 – 0.00 | 0.054 |
| **Random Effects** | | | | | | | | | |
| σ^2^ | 0.04 | | | 0.03 | | | 0.04 | | |
| τ_00_ | 0.01 _id_ | | | 0.00 _id_ | | | 0.01 _id_ | | |
| ICC | 0.19 | | | 0.06 | | | 0.19 | | |
| N | 166 _id_ | | | 166 _id_ | | | 166 _id_ | | |
| Observations | 1992 | | | 1992 | | | 1992 | | |
| Marginal R^2^ / Conditional R^2^ | 0.319 / 0.449 | | | 0.740 / 0.757 | | | 0.482 / 0.578 | | |

Table S 30. Communion and Agency by group and BSM (privately educated as reference group).

|  | **Communion** | | | | | **Agency** | | | | | | |  |  |
| --- | --- | --- | --- | --- | --- | --- | --- | --- | --- | --- | --- | --- | --- | --- |
| *Predictors* | *Estimates* | *CI* | | *p* | | | *Estimates* | | *CI* | | *p* | | |  |
| (Intercept) | 0.33 | | 0.30 – 0.37 | | **<0.001** | | | 0.72 | | 0.68 – 0.75 | | **<0.001** | | |
| State Educated | 0.35 | | 0.30 – 0.40 | | **<0.001** | | | -0.33 | | -0.38 – -0.29 | | **<0.001** | | |
| Upper Class | -0.07 | | -0.12 – -0.02 | | **0.003** | | | 0.07 | | 0.02 – 0.11 | | **0.005** | | |
| Working Class | 0.34 | | 0.30 – 0.39 | | **<0.001** | | | -0.36 | | -0.41 – -0.32 | | **<0.001** | | |
| Rich | -0.08 | | -0.12 – -0.03 | | **0.001** | | | 0.08 | | 0.04 – 0.13 | | **0.001** | | |
| Poor | 0.37 | | 0.32 – 0.41 | | **<0.001** | | | -0.51 | | -0.56 – -0.47 | | **<0.001** | | |
| Higher Educated | 0.11 | | 0.06 – 0.16 | | **<0.001** | | | -0.09 | | -0.13 – -0.04 | | **<0.001** | | |
| Lower Educated | 0.35 | | 0.31 – 0.40 | | **<0.001** | | | -0.45 | | -0.50 – -0.41 | | **<0.001** | | |
| Politicians | -0.19 | | -0.24 – -0.15 | | **<0.001** | | | 0.11 | | 0.06 – 0.16 | | **<0.001** | | |
| Doctors | 0.37 | | 0.33 – 0.42 | | **<0.001** | | | -0.22 | | -0.27 – -0.17 | | **<0.001** | | |
| Elderly | 0.43 | | 0.39 – 0.48 | | **<0.001** | | | -0.43 | | -0.47 – -0.38 | | **<0.001** | | |
| Homeless | 0.31 | | 0.26 – 0.35 | | **<0.001** | | | -0.61 | | -0.65 – -0.56 | | **<0.001** | | |
| BSM | 0.06 | | 0.04 – 0.09 | | **<0.001** | | | -0.05 | | -0.08 – -0.03 | | **<0.001** | | |
| Age centered | -0.00 | | -0.00 – 0.00 | | 0.053 | | | 0.00 | | -0.00 – 0.00 | | 0.080 | | |
| Male | -0.02 | | -0.05 – 0.01 | | 0.164 | | | 0.00 | | -0.02 – 0.03 | | 0.892 | | |
| Political Orientation centered | -0.00 | | -0.01 – 0.01 | | 0.882 | | | -0.01 | | -0.01 – 0.00 | | 0.075 | | |
| State Educated*BSM | -0.06 | | -0.09 – -0.02 | | **0.003** | | | 0.07 | | 0.03 – 0.10 | | **0.001** | | |
| Upper Class*BSM | -0.06 | | -0.10 – -0.02 | | **0.002** | | | 0.05 | | 0.01 – 0.09 | | **0.009** | | |
| Working Class*BSM | -0.08 | | -0.12 – -0.04 | | **<0.001** | | | 0.07 | | 0.03 – 0.11 | | **<0.001** | | |
| Rich*BSM | -0.04 | | -0.08 – -0.01 | | **0.022** | | | 0.02 | | -0.02 – 0.06 | | 0.307 | | |
| Poor*BSM | -0.03 | | -0.07 – 0.01 | | 0.131 | | | 0.06 | | 0.02 – 0.10 | | **0.001** | | |
| Higher Educated*BSM | -0.09 | | -0.12 – -0.05 | | **<0.001** | | | 0.06 | | 0.02 – 0.10 | | **0.001** | | |
| Lower Educated*BSM | -0.07 | | -0.10 – -0.03 | | **0.001** | | | 0.06 | | 0.02 – 0.09 | | **0.002** | | |
| Politicians*BSM | -0.04 | | -0.07 – 0.00 | | 0.063 | | | 0.02 | | -0.02 – 0.05 | | 0.418 | | |
| Doctors*BSM | -0.06 | | -0.10 – -0.02 | | **0.002** | | | 0.07 | | 0.04 – 0.11 | | **<0.001** | | |
| Elderly*BSM | -0.06 | | -0.10 – -0.02 | | **0.002** | | | 0.06 | | 0.02 – 0.09 | | **0.003** | | |
| Homeless*BSM | -0.07 | | -0.11 – -0.03 | | **<0.001** | | | 0.08 | | 0.05 – 0.12 | | **<0.001** | | |
| **Random Effects** | | | | | | | | | | | | |  |  |
| σ^2^ | 0.05 | | | | | 0.05 | | | | | | |  |  |
| τ_00_ | 0.01 _id_ | | | | | 0.00 _id_ | | | | | | |  |  |
| ICC | 0.10 | | | | | 0.08 | | | | | | |  |  |
| N | 166 _id_ | | | | | 166 _id_ | | | | | | |  |  |
| Observations | 1992 | | | | | 1992 | | | | | | |  |  |
| Marginal R^2^ / Conditional R^2^ | 0.472 / 0.527 | | | | | 0.555 / 0.589 | | | | | | |  |  |

Table S 31. Communion and Agency by group and BSM (privately educated as reference group).

|  | **Entitativity** | | | **Permeability** | | | **Status** | | |
| --- | --- | --- | --- | --- | --- | --- | --- | --- | --- |
| *Predictors* | *Estimates* | *CI* | *p* | *Estimates* | *CI* | *p* | *Estimates* | *CI* | *p* |
| (Intercept) | 0.74 | 0.69 – 0.79 | **<0.001** | 0.23 | 0.20 – 0.27 | **<0.001** | 0.83 | 0.81 – 0.86 | **<0.001** |
| State Educated | -0.23 | -0.28 – -0.18 | **<0.001** | 0.62 | 0.57 – 0.67 | **<0.001** | -0.32 | -0.36 – -0.29 | **<0.001** |
| Upper Class | 0.04 | -0.01 – 0.09 | 0.169 | -0.08 | -0.13 – -0.03 | **0.001** | 0.05 | 0.01 – 0.08 | **0.004** |
| Working Class | -0.08 | -0.13 – -0.03 | **0.003** | 0.49 | 0.45 – 0.54 | **<0.001** | -0.47 | -0.50 – -0.43 | **<0.001** |
| Rich | -0.00 | -0.05 – 0.05 | 0.913 | -0.03 | -0.08 – 0.02 | 0.223 | 0.06 | 0.03 – 0.09 | **<0.001** |
| Poor | -0.25 | -0.30 – -0.20 | **<0.001** | 0.55 | 0.50 – 0.60 | **<0.001** | -0.72 | -0.76 – -0.69 | **<0.001** |
| Higher Educated | -0.09 | -0.14 – -0.04 | **0.001** | 0.15 | 0.10 – 0.19 | **<0.001** | -0.02 | -0.05 – 0.02 | 0.356 |
| Lower Educated | -0.28 | -0.33 – -0.23 | **<0.001** | 0.43 | 0.38 – 0.48 | **<0.001** | -0.63 | -0.66 – -0.60 | **<0.001** |
| Politicians | 0.09 | 0.04 – 0.14 | **0.001** | 0.06 | 0.01 – 0.11 | **0.012** | 0.00 | -0.03 – 0.03 | 0.911 |
| Doctors | 0.03 | -0.02 – 0.08 | 0.177 | -0.10 | -0.15 – -0.05 | **<0.001** | 0.00 | -0.03 – 0.04 | 0.859 |
| Elderly | -0.04 | -0.09 – 0.01 | 0.107 | 0.46 | 0.42 – 0.51 | **<0.001** | -0.41 | -0.44 – -0.38 | **<0.001** |
| Homeless | -0.16 | -0.21 – -0.11 | **<0.001** | 0.44 | 0.39 – 0.48 | **<0.001** | -0.77 | -0.80 – -0.73 | **<0.001** |
| BSM | -0.01 | -0.04 – 0.03 | 0.637 | 0.02 | -0.00 – 0.05 | 0.091 | -0.04 | -0.06 – -0.02 | **<0.001** |
| Age centered | 0.00 | 0.00 – 0.00 | **0.030** | 0.00 | -0.00 – 0.00 | 0.963 | 0.00 | 0.00 – 0.00 | **0.006** |
| Male | -0.02 | -0.07 – 0.03 | 0.365 | -0.02 | -0.05 – 0.01 | 0.232 | -0.03 | -0.04 – -0.01 | **0.005** |
| Political Orientation centered | 0.01 | -0.00 – 0.02 | 0.066 | -0.01 | -0.01 – -0.00 | **0.026** | -0.00 | -0.01 – 0.00 | 0.075 |
| State Educated*BSM | 0.01 | -0.03 – 0.05 | 0.622 | -0.02 | -0.06 – 0.02 | 0.282 | 0.05 | 0.03 – 0.08 | **<0.001** |
| Upper Class*BSM | -0.02 | -0.06 – 0.02 | 0.399 | 0.02 | -0.02 – 0.05 | 0.396 | 0.02 | -0.01 – 0.04 | 0.214 |
| Working Class*BSM | -0.00 | -0.05 – 0.04 | 0.818 | -0.01 | -0.05 – 0.03 | 0.586 | 0.07 | 0.04 – 0.09 | **<0.001** |
| Rich*BSM | 0.00 | -0.04 – 0.04 | 0.856 | 0.01 | -0.03 – 0.05 | 0.509 | 0.02 | -0.01 – 0.04 | 0.184 |
| Poor*BSM | -0.00 | -0.04 – 0.04 | 0.878 | -0.05 | -0.09 – -0.02 | **0.006** | 0.07 | 0.04 – 0.10 | **<0.001** |
| Higher Educated*BSM | 0.03 | -0.01 – 0.07 | 0.169 | 0.02 | -0.02 – 0.05 | 0.412 | 0.03 | 0.01 – 0.06 | **0.010** |
| Lower Educated*BSM | 0.00 | -0.04 – 0.04 | 0.883 | -0.06 | -0.10 – -0.03 | **0.001** | 0.06 | 0.04 – 0.09 | **<0.001** |
| Politicians*BSM | -0.00 | -0.04 – 0.04 | 0.969 | 0.00 | -0.04 – 0.04 | 0.925 | 0.03 | 0.01 – 0.06 | **0.014** |
| Doctors*BSM | 0.02 | -0.02 – 0.06 | 0.250 | -0.01 | -0.05 – 0.03 | 0.688 | 0.04 | 0.01 – 0.07 | **0.004** |
| Elderly*BSM | 0.02 | -0.02 – 0.06 | 0.409 | -0.03 | -0.07 – 0.01 | 0.168 | 0.04 | 0.01 – 0.06 | **0.004** |
| Homeless*BSM | 0.02 | -0.02 – 0.07 | 0.233 | -0.04 | -0.08 – -0.01 | **0.025** | 0.05 | 0.03 – 0.08 | **<0.001** |
| **Random Effects** | | | | | | | | | |
| σ^2^ | 0.05 | | | 0.05 | | | 0.02 | | |
| τ_00_ | 0.02 _id_ | | | 0.01 _id_ | | | 0.00 _id_ | | |
| ICC | 0.26 | | | 0.10 | | | 0.07 | | |
| N | 166 _id_ | | | 166 _id_ | | | 166 _id_ | | |
| Observations | 1992 | | | 1992 | | | 1992 | | |
| Marginal R^2^ / Conditional R^2^ | 0.176 / 0.393 | | | 0.567 / 0.612 | | | 0.796 / 0.810 | | |

Table S 32. Interdependence, influence, and conflict by group and BSM (privately educated as reference group).

|  | **Interdependence** | | | **Influence** | | | **Conflict** | | |
| --- | --- | --- | --- | --- | --- | --- | --- | --- | --- |
| *Predictors* | *Estimates* | *CI* | *p* | *Estimates* | *CI* | *p* | *Estimates* | *CI* | *p* |
| (Intercept) | 0.35 | 0.31 – 0.38 | **<0.001** | 0.84 | 0.82 – 0.87 | **<0.001** | 0.65 | 0.61 – 0.68 | **<0.001** |
| State Educated | 0.22 | 0.18 – 0.26 | **<0.001** | -0.40 | -0.44 – -0.36 | **<0.001** | -0.41 | -0.45 – -0.36 | **<0.001** |
| Upper Class | -0.03 | -0.07 – 0.01 | 0.147 | 0.05 | 0.02 – 0.09 | **0.004** | 0.05 | 0.01 – 0.10 | **0.014** |
| Working Class | 0.24 | 0.19 – 0.28 | **<0.001** | -0.45 | -0.49 – -0.42 | **<0.001** | -0.37 | -0.41 – -0.32 | **<0.001** |
| Rich | 0.01 | -0.03 – 0.05 | 0.710 | 0.04 | 0.01 – 0.08 | **0.019** | 0.07 | 0.03 – 0.12 | **0.001** |
| Poor | -0.05 | -0.09 – -0.01 | **0.028** | -0.69 | -0.72 – -0.65 | **<0.001** | -0.39 | -0.43 – -0.35 | **<0.001** |
| Higher Educated | 0.16 | 0.12 – 0.20 | **<0.001** | -0.09 | -0.12 – -0.05 | **<0.001** | -0.16 | -0.21 – -0.12 | **<0.001** |
| Lower Educated | -0.01 | -0.05 – 0.03 | 0.548 | -0.63 | -0.67 – -0.60 | **<0.001** | -0.40 | -0.45 – -0.36 | **<0.001** |
| Politicians | 0.06 | 0.02 – 0.10 | **0.005** | 0.03 | -0.01 – 0.06 | 0.170 | 0.10 | 0.06 – 0.15 | **<0.001** |
| Doctors | 0.25 | 0.21 – 0.29 | **<0.001** | -0.16 | -0.20 – -0.12 | **<0.001** | -0.41 | -0.45 – -0.36 | **<0.001** |
| Elderly | -0.00 | -0.04 – 0.04 | 0.943 | -0.48 | -0.52 – -0.44 | **<0.001** | -0.34 | -0.39 – -0.30 | **<0.001** |
| Homeless | -0.27 | -0.31 – -0.23 | **<0.001** | -0.78 | -0.82 – -0.75 | **<0.001** | -0.51 | -0.55 – -0.47 | **<0.001** |
| BSM | 0.01 | -0.02 – 0.04 | 0.514 | -0.04 | -0.06 – -0.01 | **0.001** | -0.08 | -0.11 – -0.05 | **<0.001** |
| Age centered | 0.00 | -0.00 – 0.00 | 0.400 | 0.00 | 0.00 – 0.00 | **0.004** | 0.00 | -0.00 – 0.00 | 0.281 |
| Male | 0.01 | -0.02 – 0.05 | 0.436 | -0.01 | -0.03 – 0.01 | 0.264 | 0.03 | -0.00 – 0.07 | 0.083 |
| Political Orientation centered | -0.01 | -0.02 – -0.01 | **<0.001** | -0.00 | -0.01 – 0.00 | 0.228 | -0.00 | -0.01 – 0.00 | 0.274 |
| State Educated*BSM | -0.03 | -0.06 – 0.01 | 0.131 | 0.04 | 0.01 – 0.07 | **0.007** | 0.09 | 0.05 – 0.12 | **<0.001** |
| Upper Class*BSM | 0.01 | -0.02 – 0.05 | 0.476 | 0.02 | -0.01 – 0.05 | 0.299 | 0.04 | 0.00 – 0.07 | **0.030** |
| Working Class*BSM | -0.02 | -0.05 – 0.01 | 0.236 | 0.06 | 0.03 – 0.09 | **<0.001** | 0.10 | 0.06 – 0.13 | **<0.001** |
| Rich*BSM | -0.01 | -0.04 – 0.03 | 0.722 | 0.02 | -0.01 – 0.05 | 0.295 | 0.05 | 0.02 – 0.09 | **0.003** |
| Poor*BSM | -0.02 | -0.05 – 0.01 | 0.261 | 0.06 | 0.03 – 0.09 | **<0.001** | 0.06 | 0.02 – 0.09 | **0.002** |
| Higher Educated*BSM | -0.01 | -0.04 – 0.02 | 0.542 | 0.04 | 0.01 – 0.06 | **0.019** | 0.08 | 0.04 – 0.11 | **<0.001** |
| Lower Educated*BSM | -0.03 | -0.06 – 0.01 | 0.117 | 0.07 | 0.04 – 0.10 | **<0.001** | 0.07 | 0.03 – 0.10 | **<0.001** |
| Politicians*BSM | -0.01 | -0.04 – 0.03 | 0.704 | 0.01 | -0.02 – 0.04 | 0.401 | 0.05 | 0.01 – 0.09 | **0.006** |
| Doctors*BSM | 0.01 | -0.03 – 0.04 | 0.632 | 0.06 | 0.03 – 0.09 | **<0.001** | 0.09 | 0.06 – 0.13 | **<0.001** |
| Elderly*BSM | 0.03 | -0.01 – 0.06 | 0.126 | 0.03 | 0.00 – 0.06 | **0.031** | 0.06 | 0.02 – 0.09 | **0.002** |
| Homeless*BSM | 0.01 | -0.02 – 0.04 | 0.574 | 0.04 | 0.01 – 0.07 | **0.007** | 0.07 | 0.04 – 0.11 | **<0.001** |
| **Random Effects** | | | | | | | | | |
| σ^2^ | 0.04 | | | 0.03 | | | 0.04 | | |
| τ_00_ | 0.01 _id_ | | | 0.00 _id_ | | | 0.01 _id_ | | |
| ICC | 0.19 | | | 0.07 | | | 0.18 | | |
| N | 166 _id_ | | | 166 _id_ | | | 166 _id_ | | |
| Observations | 1992 | | | 1992 | | | 1992 | | |
| Marginal R^2^ / Conditional R^2^ | 0.324 / 0.454 | | | 0.745 / 0.763 | | | 0.496 / 0.588 | | |

## Study 2

Table S 33. Fixed-Effects ANOVA results.

| Criterion | Predictor | Sum of Squares | *df* | Mean Square | *F* | *p* | _partial_ η^2^ | _partial_ η^2^  90% CI  [LL, UL] |
| --- | --- | --- | --- | --- | --- | --- | --- | --- |
| Trust in Council | (Intercept) | 39.34 | 1 | 39.34 | 1290.06 | .000 |  |  |
|  | Private vs. Control | 0.20 | 1 | 0.20 | 6.64 | .010 | .01 | [.00, .03] |
|  | Private vs. State | 0.28 | 1 | 0.28 | 9.28 | .002 | .02 | [.00, .04] |
|  | Age | 0.07 | 1 | 0.07 | 2.40 | .122 | .00 | [.00, .02] |
|  | Political Orientation | 0.07 | 1 | 0.07 | 2.36 | .125 | .00 | [.00, .02] |
|  | Gender | 0.00 | 1 | 0.00 | 0.01 | .918 | .00 | [.00, .00] |
|  | Error | 15.25 | 500 | 0.03 |  |  |  |  |
| Representation | (Intercept) | 26.27 | 1 | 26.27 | 876.63 | .000 |  |  |
|  | Private vs. Control | 0.52 | 1 | 0.52 | 17.36 | .000 | .03 | [.01, .06] |
|  | Private vs. State | 0.52 | 1 | 0.52 | 17.36 | .000 | .03 | [.01, .06] |
|  | Age | 0.00 | 1 | 0.00 | 0.04 | .843 | .00 | [.00, .00] |
|  | Political Orientation | 0.47 | 1 | 0.47 | 15.67 | .000 | .03 | [.01, .06] |
|  | Gender | 0.03 | 1 | 0.03 | 0.84 | .361 | .00 | [.00, .01] |
|  | Error | 14.98 | 500 | 0.03 |  |  |  |  |
| Communion | (Intercept) | 33.86 | 1 | 33.86 | 937.23 | .000 |  |  |
|  | Private vs. Control | 1.93 | 1 | 1.93 | 53.42 | .000 | .10 | [.06, .14] |
|  | Private vs. State | 1.81 | 1 | 1.81 | 50.19 | .000 | .09 | [.05, .13] |
|  | Age | 0.01 | 1 | 0.01 | 0.19 | .666 | .00 | [.00, .01] |
|  | Political Orientation | 0.03 | 1 | 0.03 | 0.96 | .328 | .00 | [.00, .01] |
|  | Gender | 0.05 | 1 | 0.05 | 1.35 | .246 | .00 | [.00, .02] |
|  | Error | 18.06 | 500 | 0.04 |  |  |  |  |
| Agency | (Intercept) | 52.85 | 1 | 52.85 | 2212.78 | .000 |  |  |
|  | Private vs. Control | 0.00 | 1 | 0.00 | 0.00 | .956 | .00 | [.00, 1.00] |
|  | Private vs. State | 0.04 | 1 | 0.04 | 1.53 | .217 | .00 | [.00, .02] |
|  | Age | 0.00 | 1 | 0.00 | 0.08 | .773 | .00 | [.00, .01] |
|  | Political Orientation | 0.07 | 1 | 0.07 | 2.96 | .086 | .01 | [.00, .02] |
|  | Gender | 0.04 | 1 | 0.04 | 1.48 | .224 | .00 | [.00, .02] |
|  | Error | 11.94 | 500 | 0.02 |  |  |  |  |

*Note.* LL and UL represent the lower-limit and upper-limit of the partial η^2^ confidence interval, respectively.

Table S 34. IA Condition and BSM on Trust.

| Predictor | *b* | *b*  95% CI  [LL, UL] | *sr^2^* | *sr^2^*  95% CI  [LL, UL] | Fit |
| --- | --- | --- | --- | --- | --- |
| (Intercept) | 0.57** | [0.54, 0.60] |  |  |  |
| Private vs. Control | 0.05* | [0.01, 0.08] | .01 | [-.01, .03] |  |
| Private vs. State | 0.06** | [0.02, 0.10] | .02 | [-.00, .04] |  |
| Belief in School Meritocracy | 0.05** | [0.02, 0.07] | .02 | [-.00, .04] |  |
| Age | 0.00 | [-0.00, 0.00] | .00 | [-.01, .01] |  |
| Political Orientation | 0.00 | [-0.01, 0.01] | .00 | [-.00, .00] |  |
| Gender | -0.01 | [-0.04, 0.02] | .00 | [-.00, .01] |  |
| Private vs. Control * BSM | 0.00 | [-0.04, 0.04] | .00 | [-.00, .00] |  |
| Private vs. State * BSM | -0.05* | [-0.09, -0.01] | .01 | [-.01, .03] |  |
|  |  |  |  |  | *R^2^*  = .069** |
|  |  |  |  |  | 95% CI[.02,.10] |
|  |  |  |  |  |  |

*Note.* A significant *b*-weight indicates the semi-partial correlation is also significant. *b* represents unstandardized regression weights. *sr^2^* represents the semi-partial correlation squared. *LL* and *UL* indicate the lower and upper limits of a confidence interval, respectively.
* indicates p < .05. ** indicates p < .01.

Table S 35. IA Condition and BSM on Representation.

| Predictor | *b* | *b*  95% CI  [LL, UL] | *sr^2^* | *sr^2^*  95% CI  [LL, UL] | Fit |
| --- | --- | --- | --- | --- | --- |
| (Intercept) | 0.47** | [0.44, 0.50] |  |  |  |
| Private vs. Control | 0.08** | [0.04, 0.11] | .03 | [.00, .06] |  |
| Private vs. State | 0.08** | [0.04, 0.11] | .03 | [.00, .06] |  |
| Belief in School Meritocracy | 0.06** | [0.03, 0.09] | .03 | [.00, .06] |  |
| Age | -0.00 | [-0.00, 0.00] | .00 | [-.00, .00] |  |
| Political Orientation | 0.01* | [0.00, 0.01] | .01 | [-.01, .02] |  |
| Gender | -0.03 | [-0.06, 0.01] | .00 | [-.01, .02] |  |
| Type of Own Education | -0.03 | [-0.07, 0.01] | .00 | [-.01, .01] |  |
| Private vs. Control * BSM | -0.04* | [-0.08, -0.00] | .01 | [-.01, .02] |  |
| Private vs. State * BSM |  |  |  |  | *R^2^*  = .113** |
|  |  |  |  |  | 95% CI[.05,.15] |
|  |  |  |  |  | 95% CI[.05,.14] |
|  |  |  |  |  |  |

*Note.* A significant *b*-weight indicates the semi-partial correlation is also significant. *b* represents unstandardized regression weights. *sr^2^* represents the semi-partial correlation squared. *LL* and *UL* indicate the lower and upper limits of a confidence interval, respectively.
* indicates p < .05. ** indicates p < .01.

Table S 36. IA Condition and BSM on Agency.

| Predictor | *b* | *b*  95% CI  [LL, UL] | *sr^2^* | *sr^2^*  95% CI  [LL, UL] | Fit |
| --- | --- | --- | --- | --- | --- |
| (Intercept) | 0.66** | [0.63, 0.69] |  |  |  |
| Private vs. Control | -0.00 | [-0.03, 0.03] | .00 | [-.00, .00] |  |
| Private vs. State | 0.02 | [-0.01, 0.05] | .00 | [-.01, .01] |  |
| Belief in School Meritocracy | 0.04** | [0.02, 0.07] | .03 | [-.00, .05] |  |
| Age | -0.00 | [-0.00, 0.00] | .00 | [-.00, .00] |  |
| Political Orientation | 0.00 | [-0.01, 0.01] | .00 | [-.00, .00] |  |
| Gender | -0.03 | [-0.05, 0.00] | .01 | [-.01, .02] |  |
| Private vs. Control * BSM | -0.02 | [-0.05, 0.02] | .00 | [-.01, .01] |  |
| Private vs. State * BSM | -0.04* | [-0.07, -0.00] | .01 | [-.01, .02] |  |
|  |  |  |  |  | *R^2^*  = .046** |
|  |  |  |  |  | 95% CI[.01,.07] |
|  |  |  |  |  |  |

*Note.* A significant *b*-weight indicates the semi-partial correlation is also significant. *b* represents unstandardized regression weights. *sr^2^* represents the semi-partial correlation squared. *LL* and *UL* indicate the lower and upper limits of a confidence interval, respectively.
* indicates p < .05. ** indicates p < .01.

Table S 37. IA Condition and BSM on Communion.

| Predictor | *b* | *b*  95% CI  [LL, UL] | *sr^2^* | *sr^2^*  95% CI  [LL, UL] | Fit |
| --- | --- | --- | --- | --- | --- |
| (Intercept) | 0.53** | [0.50, 0.57] |  |  |  |
| Private vs. Control | 0.15** | [0.11, 0.19] | .09 | [.04, .14] |  |
| Private vs. State | 0.15** | [0.11, 0.19] | .08 | [.04, .13] |  |
| Belief in School Meritocracy | 0.07** | [0.04, 0.10] | .04 | [.01, .07] |  |
| Age | -0.00 | [-0.00, 0.00] | .00 | [-.00, .00] |  |
| Political Orientation | -0.00 | [-0.01, 0.00] | .00 | [-.00, .01] |  |
| Gender | -0.03* | [-0.07, -0.00] | .01 | [-.01, .02] |  |
| Private vs. Control * BSM | -0.04 | [-0.08, 0.00] | .01 | [-.01, .02] |  |
| Private vs. State * BSM | -0.04* | [-0.09, -0.00] | .01 | [-.01, .02] |  |
|  |  |  |  |  | *R^2^*  = .169** |
|  |  |  |  |  | 95% CI[.10,.22] |
|  |  |  |  |  |  |

*Note.* A significant *b*-weight indicates the semi-partial correlation is also significant. *b* represents unstandardized regression weights. *sr^2^* represents the semi-partial correlation squared. *LL* and *UL* indicate the lower and upper limits of a confidence interval, respectively.
* indicates p < .05. ** indicates p < .01.

## Study 3

Table S 38. Fixed-Effects ANOVA results

| Criterion | Predictor | Sum of Squares | *df* | Mean Square | *F* | *p* | _partial_ η^2^ | _partial_ η^2^  90% CI  [LL, UL] |
| --- | --- | --- | --- | --- | --- | --- | --- | --- |
| Trust | (Intercept) | 879.94 | 1 | 879.94 | 392.25 | .000 |  |  |
|  | Private vs. Control | 61.35 | 1 | 61.35 | 27.35 | .000 | .08 | [.04, .13] |
|  | Private vs. State | 97.54 | 1 | 97.54 | 43.48 | .000 | .12 | [.07, .18] |
|  | Age | 0.00 | 1 | 0.00 | 0.00 | .988 | .00 | [.00, 1.00] |
|  | Political Orientation | 5.13 | 1 | 5.13 | 2.29 | .132 | .01 | [.00, .03] |
|  | Gender | 0.55 | 1 | 0.55 | 0.24 | .622 | .00 | [.00, .01] |
|  | Error | 704.40 | 314 | 2.24 |  |  |  |  |
| Representation | (Intercept) | 614.96 | 1 | 614.96 | 188.85 | .000 |  |  |
|  | Private vs. Control | 275.11 | 1 | 275.11 | 84.48 | .000 | .21 | [.15, .27] |
|  | Private vs. State | 286.69 | 1 | 286.69 | 88.04 | .000 | .22 | [.16, .28] |
|  | Age | 2.78 | 1 | 2.78 | 0.85 | .356 | .00 | [.00, .02] |
|  | Political Orientation | 0.99 | 1 | 0.99 | 0.30 | .582 | .00 | [.00, .01] |
|  | Gender | 1.76 | 1 | 1.76 | 0.54 | .463 | .00 | [.00, .02] |
|  | Error | 1022.49 | 314 | 3.26 |  |  |  |  |

*Note.* LL and UL represent the lower-limit and upper-limit of the partial η^2^ confidence interval, respectively.

Table S 39. IA condition and BSM.

|  | Predictor | Sum  of  Squares | *df* | Mean  Square | *F* | *p* | _partial_ η^2^ | _partial_ η^2^  90% CI  [LL, UL] |
| --- | --- | --- | --- | --- | --- | --- | --- | --- |
| Trust | (Intercept) | 890.64 | 1 | 890.64 | 402.34 | .000 |  |  |
|  | Private vs. Control | 62.91 | 1 | 62.91 | 28.42 | .000 | .08 | [.04, .14] |
|  | Private vs. State | 98.16 | 1 | 98.16 | 44.34 | .000 | .12 | [.07, .18] |
|  | Belief in School Meritocracy | 6.08 | 1 | 6.08 | 2.75 | .098 | .01 | [.00, .03] |
|  | Age | 0.04 | 1 | 0.04 | 0.02 | .895 | .00 | [.00, .01] |
|  | Political Orientation | 1.02 | 1 | 1.02 | 0.46 | .499 | .00 | [.00, .02] |
|  | Gender | 1.29 | 1 | 1.29 | 0.58 | .446 | .00 | [.00, .02] |
|  | Private vs. Control * BSM | 0.12 | 1 | 0.12 | 0.05 | .817 | .00 | [.00, .01] |
|  | Private vs. State * BSM | 0.48 | 1 | 0.48 | 0.22 | .641 | .00 | [.00, .01] |
|  | Error | 688.44 | 311 | 2.21 |  |  |  |  |
| Representation | (Intercept) | 630.69 | 1 | 630.69 | 198.37 | .000 |  |  |
|  | Private vs. Control | 279.62 | 1 | 279.62 | 87.95 | .000 | .22 | [.16, .28] |
|  | Private vs. State | 287.86 | 1 | 287.86 | 90.54 | .000 | .23 | [.16, .29] |
|  | Belief in School Meritocracy | 13.32 | 1 | 13.32 | 4.19 | .042 | .01 | [.00, .04] |
|  | Age | 4.14 | 1 | 4.14 | 1.30 | .255 | .00 | [.00, .02] |
|  | Political Orientation | 0.58 | 1 | 0.58 | 0.18 | .669 | .00 | [.00, .01] |
|  | Gender | 0.59 | 1 | 0.59 | 0.19 | .667 | .00 | [.00, .01] |
|  | Private vs. Control * BSM | 0.07 | 1 | 0.07 | 0.02 | .885 | .00 | [.00, .01] |
|  | Private vs. State * BSM | 0.55 | 1 | 0.55 | 0.17 | .679 | .00 | [.00, .01] |
|  | Error | 988.79 | 311 | 3.18 |  |  |  |  |

*Note.* LL and UL represent the lower-limit and upper-limit of the partial η^2^ confidence interval, respectively.

## Study 4

Table S 40. Fixed-Effects ANOVA results

| Criterion | Predictor | Sum of Squares | *df* | Mean Square | *F* | *p* | _partial_ η^2^ | _partial_ η^2^  90% CI  [LL, UL] |
| --- | --- | --- | --- | --- | --- | --- | --- | --- |
| Trust in Council | (Intercept) | 32.76 | 1 | 32.76 | 738.28 | .000 |  |  |
|  | Private vs. Merit | 0.10 | 1 | 0.10 | 2.25 | .135 | .00 | [.00, .02] |
|  | Private vs. State | 1.14 | 1 | 1.14 | 25.78 | .000 | .05 | [.02, .08] |
|  | Age | 0.04 | 1 | 0.04 | 0.98 | .323 | .00 | [.00, .01] |
|  | Political Orientation | 1.18 | 1 | 1.18 | 26.50 | .000 | .05 | [.02, .08] |
|  | Gender | 0.22 | 1 | 0.22 | 5.03 | .025 | .01 | [.00, .03] |
|  | Error | 22.14 | 499 | 0.04 |  |  |  |  |
| Representation Personal | (Intercept) | 11.47 | 1 | 11.47 | 229.82 | .000 |  |  |
|  | Private vs. Merit | 0.24 | 1 | 0.24 | 4.90 | .027 | .01 | [.00, .03] |
|  | Private vs. State | 1.82 | 1 | 1.82 | 36.41 | .000 | .07 | [.04, .11] |
|  | Age | 0.00 | 1 | 0.00 | 0.04 | .841 | .00 | [.00, .00] |
|  | Political Orientation | 1.36 | 1 | 1.36 | 27.22 | .000 | .05 | [.02, .09] |
|  | Gender | 0.07 | 1 | 0.07 | 1.48 | .224 | .00 | [.00, .02] |
|  | Error | 24.91 | 499 | 0.05 |  |  |  |  |
| Representation Society | (Intercept) | 18.71 | 1 | 18.71 | 470.78 | .000 |  |  |
|  | Private vs. Merit | 0.07 | 1 | 0.07 | 1.66 | .199 | .00 | [.00, .02] |
|  | Private vs. State | 1.06 | 1 | 1.06 | 26.60 | .000 | .05 | [.02, .08] |
|  | Age | 0.01 | 1 | 0.01 | 0.20 | .658 | .00 | [.00, .01] |
|  | Political Orientation | 0.50 | 1 | 0.50 | 12.68 | .000 | .02 | [.01, .05] |
|  | Gender | 0.02 | 1 | 0.02 | 0.39 | .534 | .00 | [.00, .01] |
|  | Error | 19.83 | 499 | 0.04 |  |  |  |  |

*Note.* LL and UL represent the lower-limit and upper-limit of the partial η^2^ confidence interval, respectively.

Table S 40 continued.

| Criterion | Predictor | Sum of Squares | *df* | Mean Square | *F* | *p* | _partial_ η^2^ | _partial_ η^2^  90% CI  [LL, UL] |
| --- | --- | --- | --- | --- | --- | --- | --- | --- |
| Representation Politicians | (Intercept) | 39.05 | 1 | 39.05 | 860.73 | .000 |  |  |
|  | Private vs. Merit | 0.00 | 1 | 0.00 | 0.09 | .769 | .00 | [.00, .01] |
|  | Private vs. State | 0.78 | 1 | 0.78 | 17.09 | .000 | .03 | [.01, .06] |
|  | Age | 0.09 | 1 | 0.09 | 1.98 | .160 | .00 | [.00, .02] |
|  | Political Orientation | 0.00 | 1 | 0.00 | 0.04 | .839 | .00 | [.00, .00] |
|  | Gender | 0.16 | 1 | 0.16 | 3.57 | .059 | .01 | [.00, .02] |
|  | Error | 22.59 | 498 | 0.05 |  |  |  |  |
| Deservingness | (Intercept) | 31.65 | 1 | 31.65 | 734.51 | .000 |  |  |
|  | Private vs. Merit | 0.48 | 1 | 0.48 | 11.25 | .001 | .02 | [.01, .05] |
|  | Private vs. State | 1.41 | 1 | 1.41 | 32.63 | .000 | .06 | [.03, .10] |
|  | Age | 0.00 | 1 | 0.00 | 0.00 | .974 | .00 | [.00, 1.00] |
|  | Political Orientation | 0.68 | 1 | 0.68 | 15.73 | .000 | .03 | [.01, .06] |
|  | Gender | 0.37 | 1 | 0.37 | 8.66 | .003 | .02 | [.00, .04] |
|  | Error | 21.50 | 499 | 0.04 |  |  |  |  |

*Note.* LL and UL represent the lower-limit and upper-limit of the partial η^2^ confidence interval, respectively.

Table S 41. IA condition and BSM.

|  | Predictor | Sum  of  Squares | *df* | Mean  Square | *F* | *p* | _partial_ η^2^ | _partial_ η^2^  90% CI  [LL, UL] |
| --- | --- | --- | --- | --- | --- | --- | --- | --- |
| Trust | (Intercept) | 33.41 | 1 | 33.41 | 809.86 | .000 |  |  |
|  | Private vs. Merit | 0.13 | 1 | 0.13 | 3.05 | .081 | .01 | [.00, .02] |
|  | Private vs. State | 1.02 | 1 | 1.02 | 24.71 | .000 | .05 | [.02, .08] |
|  | Belief in School Meritocracy | 0.35 | 1 | 0.35 | 8.47 | .004 | .02 | [.00, .04] |
|  | Age | 0.01 | 1 | 0.01 | 0.35 | .553 | .00 | [.00, .01] |
|  | Political Orientation | 0.27 | 1 | 0.27 | 6.62 | .010 | .01 | [.00, .03] |
|  | Gender | 0.30 | 1 | 0.30 | 7.24 | .007 | .01 | [.00, .04] |
|  | Private vs. Merit * BSM | 0.22 | 1 | 0.22 | 5.44 | .020 | .01 | [.00, .03] |
|  | Private vs. State * BSM | 0.07 | 1 | 0.07 | 1.66 | .198 | .00 | [.00, .02] |
|  | Error | 20.46 | 496 | 0.04 |  |  |  |  |
| Representation Personal | (Intercept) | 11.95 | 1 | 11.95 | 250.18 | .000 |  |  |
|  | Private vs. Merit | 0.26 | 1 | 0.26 | 5.45 | .020 | .01 | [.00, .03] |
|  | Private vs. State | 1.65 | 1 | 1.65 | 34.64 | .000 | .07 | [.03, .10] |
|  | Belief in School Meritocracy | 0.43 | 1 | 0.43 | 8.94 | .003 | .02 | [.00, .04] |
|  | Age | 0.02 | 1 | 0.02 | 0.32 | .575 | .00 | [.00, .01] |
|  | Political Orientation | 0.38 | 1 | 0.38 | 7.94 | .005 | .02 | [.00, .04] |
|  | Gender | 0.12 | 1 | 0.12 | 2.49 | .115 | .01 | [.00, .02] |
|  | Private vs. Merit * BSM | 0.05 | 1 | 0.05 | 1.12 | .290 | .00 | [.00, .01] |
|  | Private vs. State * BSM | 0.07 | 1 | 0.07 | 1.42 | .233 | .00 | [.00, .02] |
|  | Error | 23.68 | 496 | 0.05 |  |  |  |  |

*Note.* LL and UL represent the lower-limit and upper-limit of the partial η^2^ confidence interval, respectively.

*Table S 41 continued.*

|  | Predictor | Sum  of  Squares | *df* | Mean  Square | *F* | *p* | _partial_ η^2^ | _partial_ η^2^  90% CI  [LL, UL] |
| --- | --- | --- | --- | --- | --- | --- | --- | --- |
| Representation Society | (Intercept) | 18.99 | 1 | 18.99 | 487.60 | .000 |  |  |
|  | Private vs. Merit | 0.07 | 1 | 0.07 | 1.89 | .170 | .00 | [.00, .02] |
|  | Private vs. State | 0.98 | 1 | 0.98 | 25.15 | .000 | .05 | [.02, .08] |
|  | Belief in School Meritocracy | 0.13 | 1 | 0.13 | 3.38 | .067 | .01 | [.00, .02] |
|  | Age | 0.00 | 1 | 0.00 | 0.03 | .858 | .00 | [.00, .00] |
|  | Political Orientation | 0.13 | 1 | 0.13 | 3.44 | .064 | .01 | [.00, .02] |
|  | Gender | 0.00 | 1 | 0.00 | 0.13 | .721 | .00 | [.00, .01] |
|  | Private vs. Merit * BSM | 0.04 | 1 | 0.04 | 1.15 | .285 | .00 | [.00, .01] |
|  | Private vs. State * BSM | 0.01 | 1 | 0.01 | 0.24 | .626 | .00 | [.00, .01] |
|  | Error | 19.32 | 496 | 0.04 |  |  |  |  |
| Representation Politicians | (Intercept) | 38.77 | 1 | 38.77 | 858.63 | .000 |  |  |
|  | Private vs. Merit | 0.00 | 1 | 0.00 | 0.08 | .778 | .00 | [.00, .01] |
|  | Private vs. State | 0.77 | 1 | 0.77 | 16.96 | .000 | .03 | [.01, .06] |
|  | Belief in School Meritocracy | 0.05 | 1 | 0.05 | 1.08 | .299 | .00 | [.00, .01] |
|  | Age | 0.08 | 1 | 0.08 | 1.75 | .187 | .00 | [.00, .02] |
|  | Political Orientation | 0.00 | 1 | 0.00 | 0.04 | .840 | .00 | [.00, .00] |
|  | Gender | 0.13 | 1 | 0.13 | 2.84 | .092 | .01 | [.00, .02] |
|  | Private vs. Merit * BSM | 0.02 | 1 | 0.02 | 0.39 | .535 | .00 | [.00, .01] |
|  | Private vs. State * BSM | 0.23 | 1 | 0.23 | 5.03 | .025 | .01 | [.00, .03] |
|  | Error | 22.35 | 495 | 0.05 |  |  |  |  |

*Note.* LL and UL represent the lower-limit and upper-limit of the partial η^2^ confidence interval, respectively.

*Table S 41 continued.*

|  | Predictor | Sum  of  Squares | *df* | Mean  Square | *F* | *p* | _partial_ η^2^ | _partial_ η^2^  90% CI  [LL, UL] |
| --- | --- | --- | --- | --- | --- | --- | --- | --- |
| Deservingness | (Intercept) | 32.19 | 1 | 32.19 | 791.80 | .000 |  |  |
|  | Private vs. Merit | 0.49 | 1 | 0.49 | 12.07 | .001 | .02 | [.01, .05] |
|  | Private vs. State | 1.26 | 1 | 1.26 | 30.93 | .000 | .06 | [.03, .09] |
|  | Belief in School Meritocracy | 0.70 | 1 | 0.70 | 17.12 | .000 | .03 | [.01, .06] |
|  | Age | 0.00 | 1 | 0.00 | 0.12 | .732 | .00 | [.00, .01] |
|  | Political Orientation | 0.10 | 1 | 0.10 | 2.40 | .122 | .00 | [.00, .02] |
|  | Gender | 0.42 | 1 | 0.42 | 10.43 | .001 | .02 | [.00, .05] |
|  | Private vs. Merit * BSM | 0.00 | 1 | 0.00 | 0.04 | .841 | .00 | [.00, .00] |
|  | Private vs. State * BSM | 0.35 | 1 | 0.35 | 8.68 | .003 | .02 | [.00, .04] |
|  | Error | 20.17 | 496 | 0.04 |  |  |  |  |

*Note.* LL and UL represent the lower-limit and upper-limit of the partial η^2^ confidence interval, respectively.

# Participants Education Sector as Moderator

## Study 1a

Table S 42. IA Condition and education sector on Communion, Agency, Toxicity and Trust study 1a.

|  | **Communion** | | | **Agency** | | | **Toxicity** | | | **Trust** | | |
| --- | --- | --- | --- | --- | --- | --- | --- | --- | --- | --- | --- | --- |
| *Predictors* | *Estimates* | *CI* | *p* | *Estimates* | *CI* | *p* | *Estimates* | *CI* | *p* | *Estimates* | *CI* | *p* |
| (Intercept) | 0.34 | 0.30 – 0.37 | **<0.001** | 0.33 | 0.29 – 0.36 | **<0.001** | 0.22 | 0.17 – 0.26 | **<0.001** | 0.46 | 0.40 – 0.52 | **<0.001** |
| Privately educated Politicians | -0.03 | -0.07 – 0.00 | 0.088 | 0.06 | 0.03 – 0.09 | **<0.001** | 0.07 | 0.04 – 0.10 | **<0.001** | -0.04 | -0.09 – -0.00 | **0.044** |
| Education Sector State | 0.07 | 0.03 – 0.11 | **<0.001** | 0.06 | 0.02 – 0.10 | **0.006** | -0.04 | -0.09 – 0.00 | 0.074 | 0.07 | 0.01 – 0.13 | **0.021** |
| IA (Politician * Education Sector) | -0.03 | -0.07 – 0.01 | 0.136 | -0.00 | -0.04 – 0.03 | 0.805 | 0.03 | -0.01 – 0.06 | 0.114 | -0.05 | -0.10 – -0.01 | **0.023** |
| Age (centred) | -0.00 | -0.00 – 0.00 | 0.393 | 0.00 | -0.00 – 0.00 | 0.925 | -0.00 | -0.00 – 0.00 | 0.090 | -0.00 | -0.00 – 0.00 | 0.250 |
| Male | -0.01 | -0.04 – 0.01 | 0.317 | -0.02 | -0.04 – 0.01 | 0.220 | 0.02 | -0.01 – 0.05 | 0.201 | -0.04 | -0.08 – -0.00 | **0.035** |
| LR (centred) | 0.01 | 0.01 – 0.02 | **<0.001** | 0.00 | -0.00 – 0.01 | 0.137 | -0.01 | -0.01 – 0.00 | 0.053 | 0.01 | 0.01 – 0.02 | **0.001** |
| σ^2^ | 0.01 | | | 0.01 | | | 0.01 | | | 0.02 | | |
| τ_00_ | 0.00 _id_ | | | 0.01 _id_ | | | 0.01 _id_ | | | 0.02 _id_ | | |
| ICC | 0.25 | | | 0.37 | | | 0.48 | | | 0.43 | | |
| N | 202 _id_ | | | 202 _id_ | | | 202 _id_ | | | 202 _id_ | | |
| Observations | 1211 | | | 1212 | | | 1212 | | | 1212 | | |
| Marginal R^2^ / Conditional R^2^ | 0.083 / 0.312 | | | 0.069 / 0.412 | | | 0.117 / 0.544 | | | 0.092 / 0.487 | | |

Study 2

Table S 43. IA Condition and education sector on Trust..

| Predictor | *b* | *b*  95% CI  [LL, UL] | *sr^2^* | *sr^2^*  95% CI  [LL, UL] | Fit |
| --- | --- | --- | --- | --- | --- |
| (Intercept) | 0.58** | [0.52, 0.65] |  |  |  |
| Private vs. Control | 0.10* | [0.01, 0.19] | .01 | [-.01, .02] |  |
| Private vs. State | -0.01 | [-0.10, 0.07] | .00 | [-.00, .00] |  |
| Education Sector | -0.02 | [-0.09, 0.05] | .00 | [-.00, .00] |  |
| Age | 0.00 | [-0.00, 0.00] | .00 | [-.01, .01] |  |
| Political Orientation | 0.00 | [-0.00, 0.01] | .00 | [-.01, .01] |  |
| Gender | 0.00 | [-0.03, 0.03] | .00 | [-.00, .00] |  |
| Private vs. Control * Education Sector | -0.05 | [-0.14, 0.05] | .00 | [-.00, .01] |  |
| Education Sector | 0.07 | [-0.02, 0.16] | .00 | [-.01, .01] |  |
|  |  |  |  |  | *R^2^*  = .040** |
|  |  |  |  |  | 95% CI[.01,.06] |
|  |  |  |  |  |  |

*Note.* A significant *b*-weight indicates the semi-partial correlation is also significant. *b* represents unstandardized regression weights. *sr^2^* represents the semi-partial correlation squared. *LL* and *UL* indicate the lower and upper limits of a confidence interval, respectively.
* indicates p < .05. ** indicates p < .01.

Table S 44. IA Condition and Education Sector on Representation.

| Predictor | *b* | *b*  95% CI  [LL, UL] | *sr^2^* | *sr^2^*  95% CI  [LL, UL] | Fit |
| --- | --- | --- | --- | --- | --- |
| (Intercept) | 0.51** | [0.45, 0.58] |  |  |  |
| Private vs. Control | 0.04 | [-0.05, 0.13] | .00 | [-.00, .01] |  |
| Private vs. State | 0.00 | [-0.08, 0.09] | .00 | [-.00, .00] |  |
| Education Sector | -0.05 | [-0.12, 0.02] | .00 | [-.01, .01] |  |
| Age | 0.00 | [-0.00, 0.00] | .00 | [-.00, .00] |  |
| Political Orientation | 0.01** | [0.01, 0.02] | .03 | [.00, .05] |  |
| Gender | -0.02 | [-0.04, 0.01] | .00 | [-.00, .01] |  |
| Private vs. Control * Education Sector | 0.04 | [-0.06, 0.14] | .00 | [-.00, .01] |  |
| Private vs. State * Education Sector | 0.08 | [-0.02, 0.17] | .00 | [-.01, .01] |  |
|  |  |  |  |  | *R^2^*  = .071** |
|  |  |  |  |  | 95% CI[.03,.10] |
|  |  |  |  |  | 95% CI[.05,.14] |
|  |  |  |  |  |  |

*Note.* A significant *b*-weight indicates the semi-partial correlation is also significant. *b* represents unstandardized regression weights. *sr^2^* represents the semi-partial correlation squared. *LL* and *UL* indicate the lower and upper limits of a confidence interval, respectively.
* indicates p < .05. ** indicates p < .01.

Table S 45. IA Condition and Education Sector on Agency.

| Predictor | *b* | *b*  95% CI  [LL, UL] | *sr^2^* | *sr^2^*  95% CI  [LL, UL] | Fit |
| --- | --- | --- | --- | --- | --- |
| (Intercept) | 0.65** | [0.60, 0.71] |  |  |  |
| Private vs. Control | 0.03 | [-0.05, 0.11] | .00 | [-.00, .01] |  |
| Private vs. State | -0.00 | [-0.08, 0.07] | .00 | [-.00, .00] |  |
| Education Sector | 0.00 | [-0.06, 0.06] | .00 | [-.00, .00] |  |
| Age | 0.00 | [-0.00, 0.00] | .00 | [-.00, .00] |  |
| Political Orientation | 0.01* | [0.00, 0.01] | .01 | [-.01, .02] |  |
| Gender | -0.02 | [-0.04, 0.01] | .00 | [-.01, .01] |  |
| Private vs. Control * Education Sector | -0.03 | [-0.11, 0.06] | .00 | [-.00, .00] |  |
| Private vs. State * Education Sector | 0.02 | [-0.06, 0.10] | .00 | [-.00, .00] |  |
|  |  |  |  |  | *R^2^*  = .016 |
|  |  |  |  |  | 95% CI[.00,.03] |
|  |  |  |  |  |  |

*Note.* A significant *b*-weight indicates the semi-partial correlation is also significant. *b* represents unstandardized regression weights. *sr^2^* represents the semi-partial correlation squared. *LL* and *UL* indicate the lower and upper limits of a confidence interval, respectively.
* indicates p < .05. ** indicates p < .01.

Table S 46. IA Condition and Education Sector on Communion.

| Predictor | *b* | *b*  95% CI  [LL, UL] | *sr^2^* | *sr^2^*  95% CI  [LL, UL] | Fit |
| --- | --- | --- | --- | --- | --- |
| (Intercept) | 0.51** | [0.44, 0.58] |  |  |  |
| Private vs. Control | 0.20** | [0.10, 0.29] | .02 | [.00, .05] |  |
| Private vs. State | 0.13** | [0.04, 0.22] | .01 | [-.00, .03] |  |
| Education Sector | 0.01 | [-0.06, 0.08] | .00 | [-.00, .00] |  |
| Age | 0.00 | [-0.00, 0.00] | .00 | [-.00, .00] |  |
| Political Orientation | 0.00 | [-0.00, 0.01] | .00 | [-.01, .01] |  |
| Gender | -0.02 | [-0.05, 0.01] | .00 | [-.00, .01] |  |
| Private vs. Control * Education Sector | -0.05 | [-0.15, 0.06] | .00 | [-.00, .01] |  |
| Private vs. State * Education Sector | 0.02 | [-0.08, 0.12] | .00 | [-.00, .00] |  |
|  |  |  |  |  | *R^2^*  = .137** |
|  |  |  |  |  | 95% CI[.08,.18] |
|  |  |  |  |  |  |

*Note.* A significant *b*-weight indicates the semi-partial correlation is also significant. *b* represents unstandardized regression weights. *sr^2^* represents the semi-partial correlation squared. *LL* and *UL* indicate the lower and upper limits of a confidence interval, respectively.
* indicates p < .05. ** indicates p < .01.

Study 3

Table S 47. IA condition and Education Sector Study 3.

|  | Predictor | Sum  of  Squares | *df* | Mean  Square | *F* | *p* | _partial_ η^2^ | _partial_ η^2^  90% CI  [LL, UL] |
| --- | --- | --- | --- | --- | --- | --- | --- | --- |
| Trust | (Intercept) | 393.74 | 1 | 393.74 | 180.99 | .000 |  |  |
|  | Private vs. Control | 1.62 | 1 | 1.62 | 0.75 | .388 | .00 | [.00, .02] |
|  | Private vs. State | 3.05 | 1 | 3.05 | 1.40 | .237 | .00 | [.00, .02] |
|  | Education Sector | 4.55 | 1 | 4.55 | 2.09 | .149 | .01 | [.00, .02] |
|  | Age | 0.59 | 1 | 0.59 | 0.27 | .604 | .00 | [.00, .01] |
|  | Political Orientation | 1.06 | 1 | 1.06 | 0.49 | .486 | .00 | [.00, .01] |
|  | Gender | 0.75 | 1 | 0.75 | 0.35 | .557 | .00 | [.00, .01] |
|  | Private vs. Control * Education Sector | 3.49 | 1 | 3.49 | 1.61 | .206 | .00 | [.00, .02] |
|  | Private vs. State * Education Sector | 5.34 | 1 | 5.34 | 2.45 | .118 | .01 | [.00, .03] |
|  | Error | 807.08 | 371 | 2.18 |  |  |  |  |
| Representation | (Intercept) | 279.56 | 1 | 279.56 | 90.26 | .000 |  |  |
|  | Private vs. Control | 49.72 | 1 | 49.72 | 16.05 | .000 | .04 | [.01, .08] |
|  | Private vs. State | 23.48 | 1 | 23.48 | 7.58 | .006 | .02 | [.00, .05] |
|  | Education Sector | 3.49 | 1 | 3.49 | 1.13 | .289 | .00 | [.00, .02] |
|  | Age | 1.76 | 1 | 1.76 | 0.57 | .452 | .00 | [.00, .02] |
|  | Political Orientation | 5.44 | 1 | 5.44 | 1.76 | .186 | .00 | [.00, .02] |
|  | Gender | 1.12 | 1 | 1.12 | 0.36 | .548 | .00 | [.00, .01] |
|  | Private vs. Control * Education Sector | 0.00 | 1 | 0.00 | 0.00 | .992 | .00 | [.00, 1.00] |
|  | Private vs. State * Education Sector | 5.49 | 1 | 5.49 | 1.77 | .184 | .00 | [.00, .02] |
|  | Error | 1149.05 | 371 | 3.10 |  |  |  |  |

*Note.* LL and UL represent the lower-limit and upper-limit of the partial η^2^ confidence interval, respectively.

Study 4

Table S 48. IA condition and BSM.

|  | Predictor | Sum  of  Squares | *df* | Mean  Square | *F* | *p* | _partial_ η^2^ | _partial_ η^2^  90% CI  [LL, UL] |
| --- | --- | --- | --- | --- | --- | --- | --- | --- |
| Trust | (Intercept) | 8.79 | 1 | 8.79 | 208.96 | .000 |  |  |
|  | Private vs. Merit | 0.00 | 1 | 0.00 | 0.11 | .737 | .00 | [.00, .01] |
|  | Private vs. State | 0.18 | 1 | 0.18 | 4.32 | .038 | .01 | [.00, .02] |
|  | Education Sector | 0.10 | 1 | 0.10 | 2.37 | .124 | .00 | [.00, .02] |
|  | Age | 0.01 | 1 | 0.01 | 0.28 | .600 | .00 | [.00, .01] |
|  | Political Orientation | 1.51 | 1 | 1.51 | 35.79 | .000 | .06 | [.03, .09] |
|  | Gender | 0.13 | 1 | 0.13 | 3.20 | .074 | .01 | [.00, .02] |
|  | Private vs. Merit * Education Sector | 0.00 | 1 | 0.00 | 0.10 | .748 | .00 | [.00, .01] |
|  | Private vs. State * Education Sector | 0.00 | 1 | 0.00 | 0.03 | .866 | .00 | [.00, .00] |
|  | Error | 25.15 | 598 | 0.04 |  |  |  |  |
| Representation Personal | (Intercept) | 4.02 | 1 | 4.02 | 80.09 | .000 |  |  |
|  | Private vs. Merit | 0.02 | 1 | 0.02 | 0.38 | .538 | .00 | [.00, .01] |
|  | Private vs. State | 0.24 | 1 | 0.24 | 4.83 | .028 | .01 | [.00, .02] |
|  | Education Sector | 0.20 | 1 | 0.20 | 4.00 | .046 | .01 | [.00, .02] |
|  | Age | 0.02 | 1 | 0.02 | 0.38 | .539 | .00 | [.00, .01] |
|  | Political Orientation | 1.78 | 1 | 1.78 | 35.42 | .000 | .06 | [.03, .09] |
|  | Gender | 0.01 | 1 | 0.01 | 0.21 | .647 | .00 | [.00, .01] |
|  | Private vs. Merit * Education Sector | 0.01 | 1 | 0.01 | 0.12 | .732 | .00 | [.00, .01] |
|  | Private vs. State * Education Sector | 0.01 | 1 | 0.01 | 0.14 | .704 | .00 | [.00, .01] |
|  | Error | 30.03 | 598 | 0.05 |  |  |  |  |

*Note.* LL and UL represent the lower-limit and upper-limit of the partial η^2^ confidence interval, respectively.

*Table S 48 continued.*

|  | Predictor | Sum  of  Squares | *df* | Mean  Square | *F* | *p* | _partial_ η^2^ | _partial_ η^2^  90% CI  [LL, UL] |
| --- | --- | --- | --- | --- | --- | --- | --- | --- |
| Representation Society | (Intercept) | 5.41 | 1 | 5.41 | 135.40 | .000 |  |  |
|  | Private vs. Merit | 0.11 | 1 | 0.11 | 2.82 | .093 | .00 | [.00, .02] |
|  | Private vs. State | 0.06 | 1 | 0.06 | 1.39 | .240 | .00 | [.00, .01] |
|  | Education Sector | 0.10 | 1 | 0.10 | 2.44 | .119 | .00 | [.00, .02] |
|  | Age | 0.00 | 1 | 0.00 | 0.06 | .812 | .00 | [.00, .00] |
|  | Political Orientation | 0.47 | 1 | 0.47 | 11.78 | .001 | .02 | [.01, .04] |
|  | Gender | 0.05 | 1 | 0.05 | 1.16 | .283 | .00 | [.00, .01] |
|  | Private vs. Merit * Education Sector | 0.04 | 1 | 0.04 | 1.05 | .306 | .00 | [.00, .01] |
|  | Private vs. State * Education Sector | 0.04 | 1 | 0.04 | 0.92 | .338 | .00 | [.00, .01] |
|  | Error | 23.89 | 598 | 0.04 |  |  |  |  |
| Representation Politicians | (Intercept) | 8.98 | 1 | 8.98 | 203.78 | .000 |  |  |
|  | Private vs. Merit | 0.00 | 1 | 0.00 | 0.03 | .859 | .00 | [.00, .00] |
|  | Private vs. State | 0.04 | 1 | 0.04 | 0.92 | .338 | .00 | [.00, .01] |
|  | Education Sector | 0.00 | 1 | 0.00 | 0.11 | .741 | .00 | [.00, .01] |
|  | Age | 0.07 | 1 | 0.07 | 1.61 | .205 | .00 | [.00, .01] |
|  | Political Orientation | 0.00 | 1 | 0.00 | 0.01 | .912 | .00 | [.00, .00] |
|  | Gender | 0.16 | 1 | 0.16 | 3.53 | .061 | .01 | [.00, .02] |
|  | Private vs. Merit * Education Sector | 0.00 | 1 | 0.00 | 0.00 | .982 | .00 | [.00, 1.00] |
|  | Private vs. State * Education Sector | 0.03 | 1 | 0.03 | 0.66 | .418 | .00 | [.00, .01] |
|  | Error | 26.30 | 597 | 0.04 |  |  |  |  |

*Note.* LL and UL represent the lower-limit and upper-limit of the partial η^2^ confidence interval, respectively.

*Table S 48 continued.*

|  | Predictor | Sum  of  Squares | *df* | Mean  Square | *F* | *p* | _partial_ η^2^ | _partial_ η^2^  90% CI  [LL, UL] |
| --- | --- | --- | --- | --- | --- | --- | --- | --- |
| Deservingness | (Intercept) | 8.06 | 1 | 8.06 | 191.26 | .000 |  |  |
|  | Private vs. Merit | 0.24 | 1 | 0.24 | 5.78 | .017 | .01 | [.00, .03] |
|  | Private vs. State | 0.17 | 1 | 0.17 | 3.98 | .046 | .01 | [.00, .02] |
|  | Education Sector | 0.06 | 1 | 0.06 | 1.35 | .245 | .00 | [.00, .01] |
|  | Age | 0.01 | 1 | 0.01 | 0.24 | .622 | .00 | [.00, .01] |
|  | Political Orientation | 1.10 | 1 | 1.10 | 26.14 | .000 | .04 | [.02, .07] |
|  | Gender | 0.29 | 1 | 0.29 | 6.76 | .010 | .01 | [.00, .03] |
|  | Private vs. Merit * Education Sector | 0.03 | 1 | 0.03 | 0.64 | .423 | .00 | [.00, .01] |
|  | Private vs. State * Education Sector | 0.01 | 1 | 0.01 | 0.24 | .628 | .00 | [.00, .01] |
|  | Error | 25.21 | 598 | 0.04 |  |  |  |  |

*Note.* LL and UL represent the lower-limit and upper-limit of the partial η^2^ confidence interval, respectively.
